# Supplementary material for: Syntheses and Glycosidase Inhibitory Activities, and in Silico Docking Studies of Pericosine E Analogs Methoxy-Substituted at C6
Source: Mar Drugs. 2020 Apr 20;18(4):221. doi: 10.3390/md18040221 (PMC7230162; doi:10.3390/md18040221)

Supplementary Materials

Syntheses, Glycosidase Inhibitory Activities, and *in silico* Docking Studies of Pericosine E Analogs Methoxy-Substituted at C6

Yoshihide Usami *, Megumi Higuchi, Koji Mizuki, Mizuki Yamamoto, Mao Kanki, Chika Nakasone, Yuya Sugimoto, Makio Shibano, Yoshihiro Uesawa, Junko Nagai, Hiroki Yoneyama and Shinya Harusawa

Osaka University of Pharmaceutical Sciences, Meiji Pharmaceutical University

Contents

1. Modified preparation of *anti*-epoxide (-)-**11** Page SM-3
2. Synthesis of new compound (-)-**15** Page SM-3
3. ^{1}H- and ^{13}C-NMR spectra of new compounds Page SM-4-SM-61

    Figure S1: ^{1}H- NMR Spectrum of (-)-**15** Page SM-4
    Figure S2: ^{13}C-NMR Spectrum of (-)-**15** Page SM-5
    Figure S3: ^{1}H- NMR Spectrum of (+)-**14** Page SM-6
    Figure S4: ^{13}C-NMR Spectrum of (+)-**14** Page SM-7
    Figure S5: ^{1}H- NMR Spectrum of (+)-**12** Page SM-8
    Figure S6: ^{13}C-NMR Spectrum of (+)-**12** Page SM-9
    Figure S7: ^{1}H- NMR Spectrum of (-)-**16** Page SM-10
    Figure S8: ^{13}C-NMR Spectrum of (-)-**16** Page SM-11
    Figure S9: ^{1}H- NMR Spectrum of (+)-**16** Page SM-12
    Figure S10: ^{13}C-NMR Spectrum of (+)-**16** Page SM-13
    Figure S11: ^{1}H- NMR Spectrum of (-)-**19** Page SM-14
    Figure S12: ^{13}C-NMR Spectrum of (-)-**19** Page SM-15
    Figure S13: ^{1}H- NMR Spectrum of (+)-**19**, Figure S14: ^{13}C-NMR Spectrum of (+)-**19** Page SM-16
    Figure S15: ^{1}H- NMR Spectrum of (-)-**22** Page SM-17
    Figure S16: ^{13}C-NMR Spectrum of (-)-**22** Page SM-18
    Figure S17: ^{1}H- NMR Spectrum of (+)-**22**, Figure S18: ^{13}C-NMR Spectrum of (+)-**22** Page SM-19
    Figure S19: ^{1}H- NMR Spectrum of (+)-**13** Page SM-20
    Figure S20: ^{13}C-NMR Spectrum of (+)-**13** Page SM-21
    Figure S21: ^{1}H- NMR Spectrum of (-)-**13** Page SM-22
    Figure S22: ^{13}C-NMR Spectrum of (-)-**13** Page SM-23
    Figure S23: ^{1}H- NMR Spectrum of (+)-**17** Page SM-24
    Figure S24: ^{13}C-NMR Spectrum of (+)-**17** Page SM-25
    Figure S25: ^{1}H- NMR Spectrum of (-)-**17** Page SM-26

Figure S26: ^{13}C-NMR Spectrum of (-)-**17** Page SM-27
Figure S27: ^{1}H- NMR Spectrum of (-)-**20** Page SM-28
Figure S28: ^{13}C-NMR Spectrum of (-)-**20** Page SM-29
Figure S29: ^{1}H- NMR Spectrum of (+)-**20**, Figure S30: ^{13}C-NMR Spectrum of (+)-**20** Page SM-30
Figure S31: ^{1}H- NMR Spectrum of (+)-**23** Page SM-31
Figure S32: ^{13}C-NMR Spectrum of (+)-**23** Page SM-32
Figure S33: ^{1}H- NMR Spectrum of (-)-**23**, Figure S34: ^{13}C-NMR Spectrum of (-)-**23** Page SM-33
Figure S35: ^{1}H- NMR Spectrum of (+)-**6** Page SM-34
Figure S36: ^{13}C-NMR Spectrum of (+)-**6** Page SM-35
Figure S37: ^{1}H- NMR Spectrum of (-)-**6** Page SM-36
Figure S38: ^{13}C-NMR Spectrum of (-)-**6** Page SM-37
Figure S39: ^{1}H- NMR Spectrum of (+)-**14** Page SM-38
Figure S40: ^{13}C-NMR Spectrum of (+)-**14** Page SM-39
Figure S41: ^{1}H- NMR Spectrum of (-)-**14** Page SM-40
Figure S42: ^{13}C-NMR Spectrum of (-)-**14** Page SM-41
Figure S43: ^{1}H- NMR Spectrum of (+)-**7** Page SM-42
Figure S44: ^{13}C-NMR Spectrum of (+)-**7** Page SM-43
Figure S45: ^{1}H- NMR Spectrum of (-)-**7** Page SM-44
Figure S46: ^{13}C-NMR Spectrum of (-)-**7** Page SM-45
Figure S47: ^{1}H- NMR Spectrum of (-)-**18** Page SM-46
Figure S48: ^{13}C-NMR Spectrum of (-)-**18** Page SM-47
Figure S49: ^{1}H- NMR Spectrum of (+)-**18** Page SM-48
Figure S50: ^{13}C-NMR Spectrum of (+)-**18** Page SM-49
Figure S51: ^{1}H- NMR Spectrum of (-)-**8** Page SM-50
Figure S52: ^{13}C-NMR Spectrum of (-)-**8** Page SM-51
Figure S53: ^{1}H- NMR Spectrum of (+)-**8**, Figure S54: ^{13}C-NMR Spectrum of (+)-**8** Page SM-52
Figure S55: ^{1}H- NMR Spectrum of (-)-**21** Page SM-53
Figure S56: ^{13}C-NMR Spectrum of (-)-**21** Page SM-54
Figure S57: ^{1}H- NMR Spectrum of (+)-**21**, Figure S58: ^{13}C-NMR Spectrum of (+)-**21** Page SM-55
Figure S59: ^{1}H- NMR Spectrum of (-)-**24** Page SM-56
Figure S60: ^{13}C-NMR Spectrum of (-)-**24** Page SM-57
Figure S61: ^{1}H- NMR Spectrum of (+)-**24**, Figure S62: ^{13}C-NMR Spectrum of (+)-**24** Page SM-58
Figure S63: ^{1}H- NMR Spectrum of (-)-**9** Page SM-59
Figure S64: ^{13}C-NMR Spectrum of (-)-**9** Page SM-60
Figure S65: ^{1}H- NMR Spectrum of (+)-**9**. Figure S66: ^{13}C-NMR Spectrum of (+)-**9** Page SM-61

1. Modified preparation of *anti*-epoxide (-)-11 by TFDO oxidation of diene (-)-SI-1

To a solution of diene (-)-**SI-1** (108.6 mg, 0.43 mmol) in CH_3CN (7.0 mL) and H_2O (7.0 mL) included in a micro-wave vial (10-20 mL), was added powdered $NaHCO_3$ (364 mg, 4.3 mmol) with stirring. The reaction vial was cooled at -15 °C, then 1,1,1-trifluoroacetone (0.31 mL, 3.4 mmol) and Oxone (320 mg, 0.52 mmol) were added successively without stirring. After sealing the vial, the reaction mixture was kept at -15 °C for 3 h with stirring vigorously. The reaction mixture was poured into a beaker including 20 mL of *tert*-butyl methyl ether (TBME) to make a suspension. After filtration over celite under reduced pressure, saturated aqueous $NaHCO_3$ (50 mL) was added to the filtrate. The mixture was extracted with TBME, then the organic layer was dried over $MgSO_4$, filtered, and evaporated to give a residue, which was purified by silica gel column chromatography (eluent; Hexane : AcOEt = 19:1) to afford (-)-**11** (83.9 mg, 0.32 mmol) in 74% yield.

*This procedure reduced cost of this step to 1/6 compared to the previous method [10, 11].

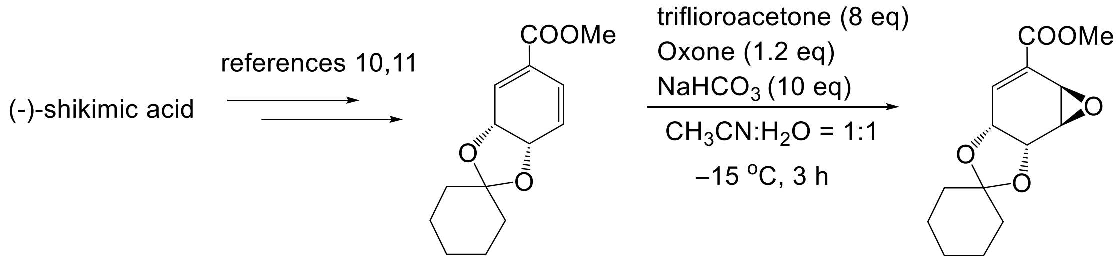

(−)-**SI-1** (−)-**11**

2. Synthesis of (-)-15

Anti-epoxide (+)-**11** (107.3 mg, 0.40 mmol) was converted to (-)-**15** (57.4 mg, 46% in 3 steps) by the same procedure in the literature [13]. (-)-**15**: Colorless oil; $[\alpha]_D^{20}$ -15.1 (*c* 0.49, $CHCl_3$); IR ν_{max} (film) 3497 (OH), 1724 (C=O), 1653 (C=C) cm^{-1}; ^{1}H-NMR (300 MHz; $CDCl_3$) δ 1.25-1.70 (10H, m), 3.17 (1H, *d*, J =11.5 Hz, 5-OH), 3.58 (3H, s, 6-OMe), 3.82 (3H, s, COOMe), 3.83 (1H, m, H-5), 4.28 (1H, d, *J* = 5.2 Hz, H-6), 4.48 (1H, dd, *J* = 5.6, 3.2 Hz, H-3), 4.66 (1H, ddd, *J* = 5.6, 3.5, 0.6 Hz, H-4), 6.83 (1H, dd, *J* = 3.2, 0.9 Hz, H-2); ^{13}C-NMR (75.5 MHz; $CDCl_3$) δ 23.7, 23.9, 24.9, 35.7, 37.2, 52.2, 61.2, 68.0, 72.1, 72.9, 74.2, 111.7, 130.0, 137.8, 166.7; HREIMS *m/z* calcd for $C_{15}H_{22}O_6$ $(M)^+$ 298.1416, found 298.1420.

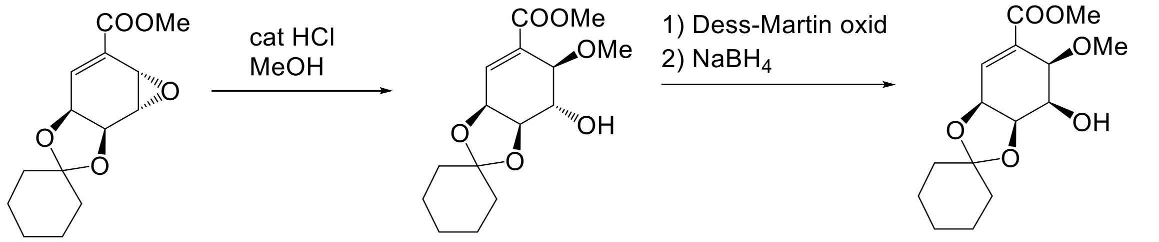

(+)-**11** (-)-**15**

3. ^{1}H- and ^{13}C-NMR spectra of new compounds

Figure S1: ^{1}H- NMR spectrum of (-)-**15** in $CDCl_3$ (300 MHz,)

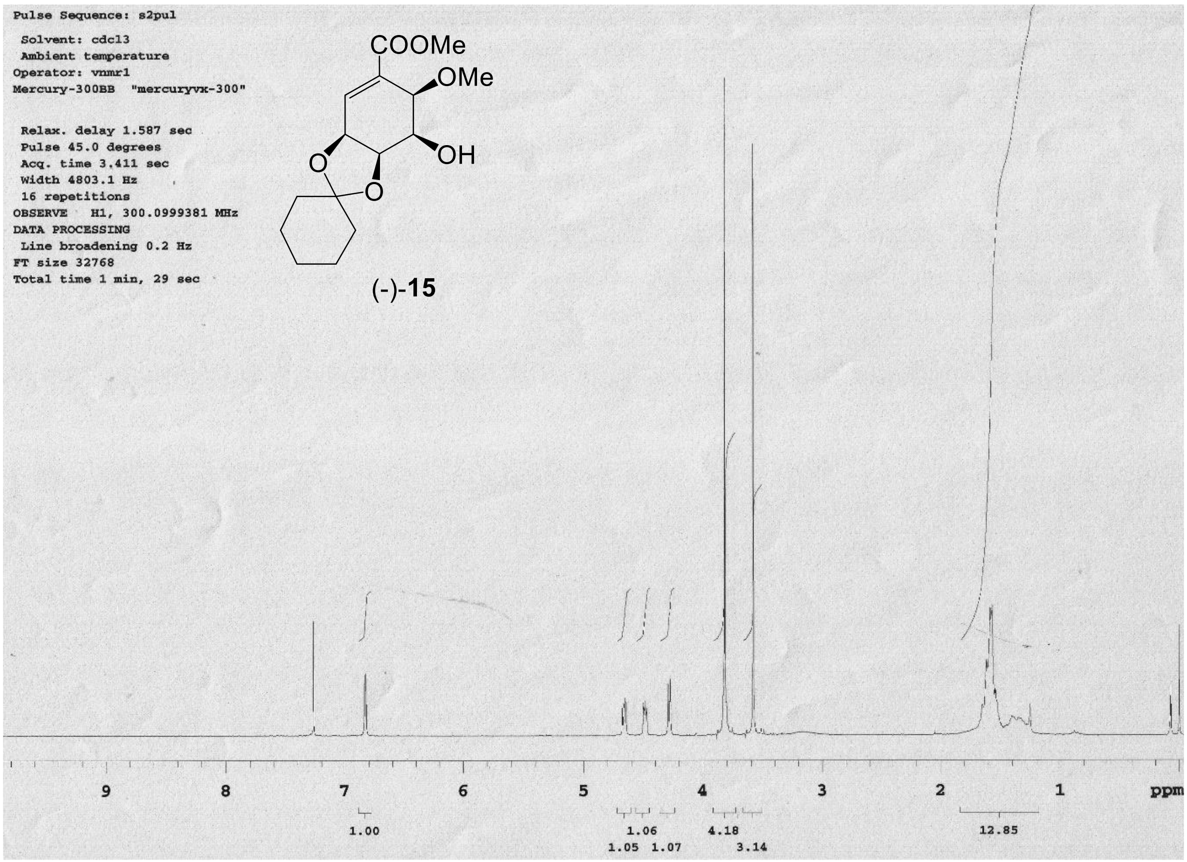

INDEX	FREQUENCY	PPM	HEIGHT
1	2181.1	7.268	134.4
2	2051.5	6.836	70.2
3	2050.6	6.833	71.7
4	2048.3	6.825	73.8
5	2047.4	6.822	67.7
6	1402.8	4.674	33.3
7	1402.2	4.672	33.9
8	1399.5	4.664	36.8
9	1398.6	4.661	38.2
10	1397.2	4.656	45.1
11	1396.3	4.653	42.8
12	1393.7	4.644	40.8
13	1393.1	4.642	39.9
14	1350.3	4.499	39.6
15	1347.1	4.489	41.7
16	1344.7	4.481	34.7
17	1341.5	4.470	33.2
18	1287.5	4.290	62.0
19	1282.3	4.273	66.9
20	1145.7	3.818	767.6
21	1141.0	3.802	41.0
22	1074.7	3.581	690.1
23	494.8	1.649	37.7
24	485.5	1.618	89.3
25	482.8	1.609	86.0
26	476.4	1.587	149.8
27	473.4	1.578	142.7
28	471.7	1.572	152.3
29	462.9	1.542	56.1
30	459.4	1.531	42.0
31	421.3	1.404	23.8
32	417.5	1.391	22.9
33	406.0	1.353	22.0
34	376.1	1.253	37.7
35	21.1	0.070	60.0
36	-0.0	-0.000	129.0

Figure S2: ^{13}C- NMR spectrum of (-)-**15** in $CDCl_3$ (75.5 MHz)

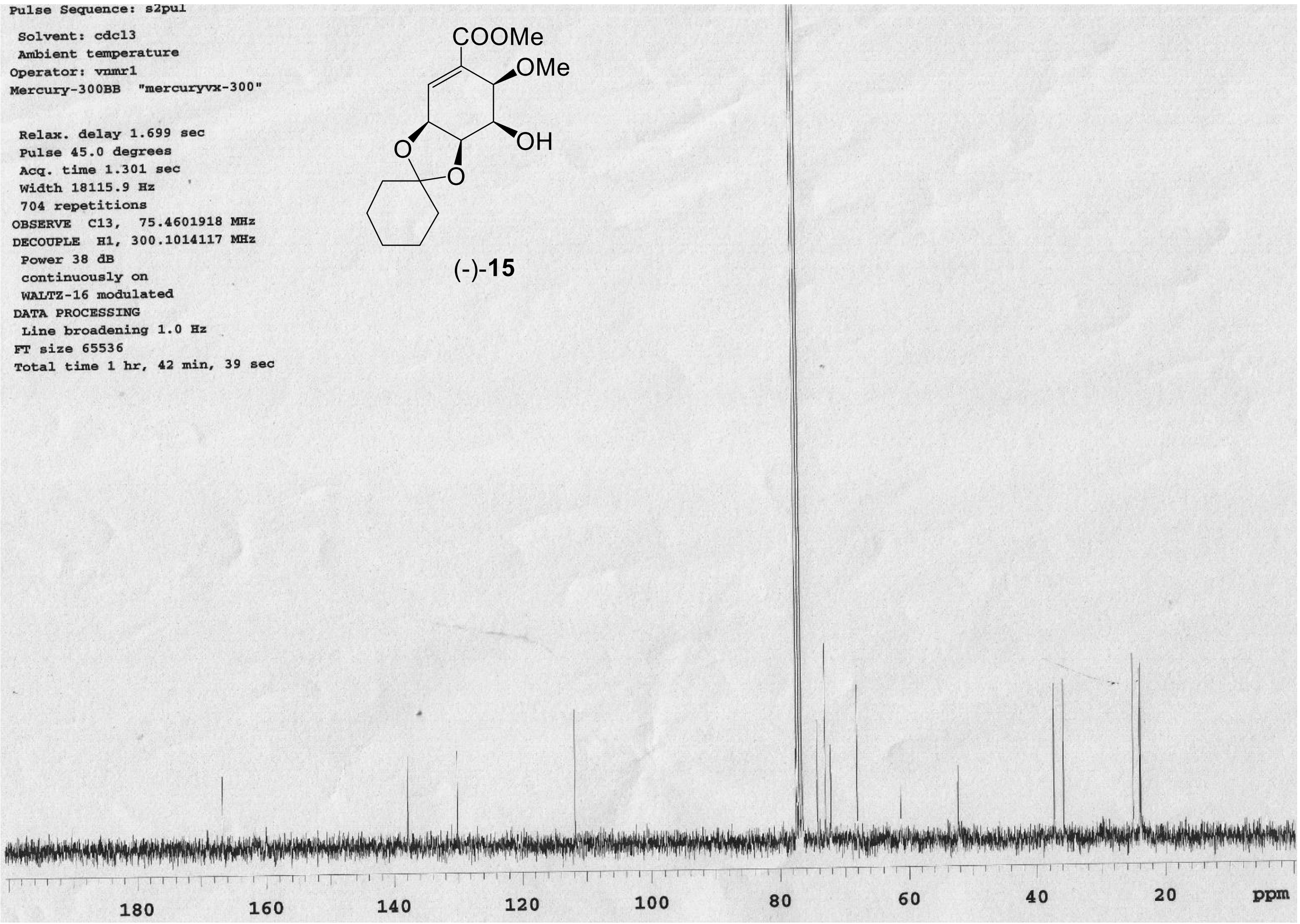

INDEX	FREQUENCY	PPM	HEIGHT
1	12576.3	166.661	14.6
2	10397.5	137.788	18.2
3	9812.6	130.036	19.1
4	8432.6	111.749	27.1
5	5842.5	77.425	168.5
6	5810.4	77.000	178.9
7	5778.4	76.575	177.9
8	5598.1	74.187	24.4
9	5503.6	72.934	39.5
10	5441.1	72.106	19.4
11	5130.4	67.989	31.1
12	4618.5	61.204	10.9
13	3937.4	52.178	14.8
14	2807.9	37.210	30.8
15	2694.0	35.701	31.7
16	1880.7	24.924	36.5
17	1801.7	23.876	33.6
18	1790.1	23.722	34.7

Figure S3: ^{1}H- NMR Spectrum of (-)-**12** in acetone-d_6 (400 MHz)

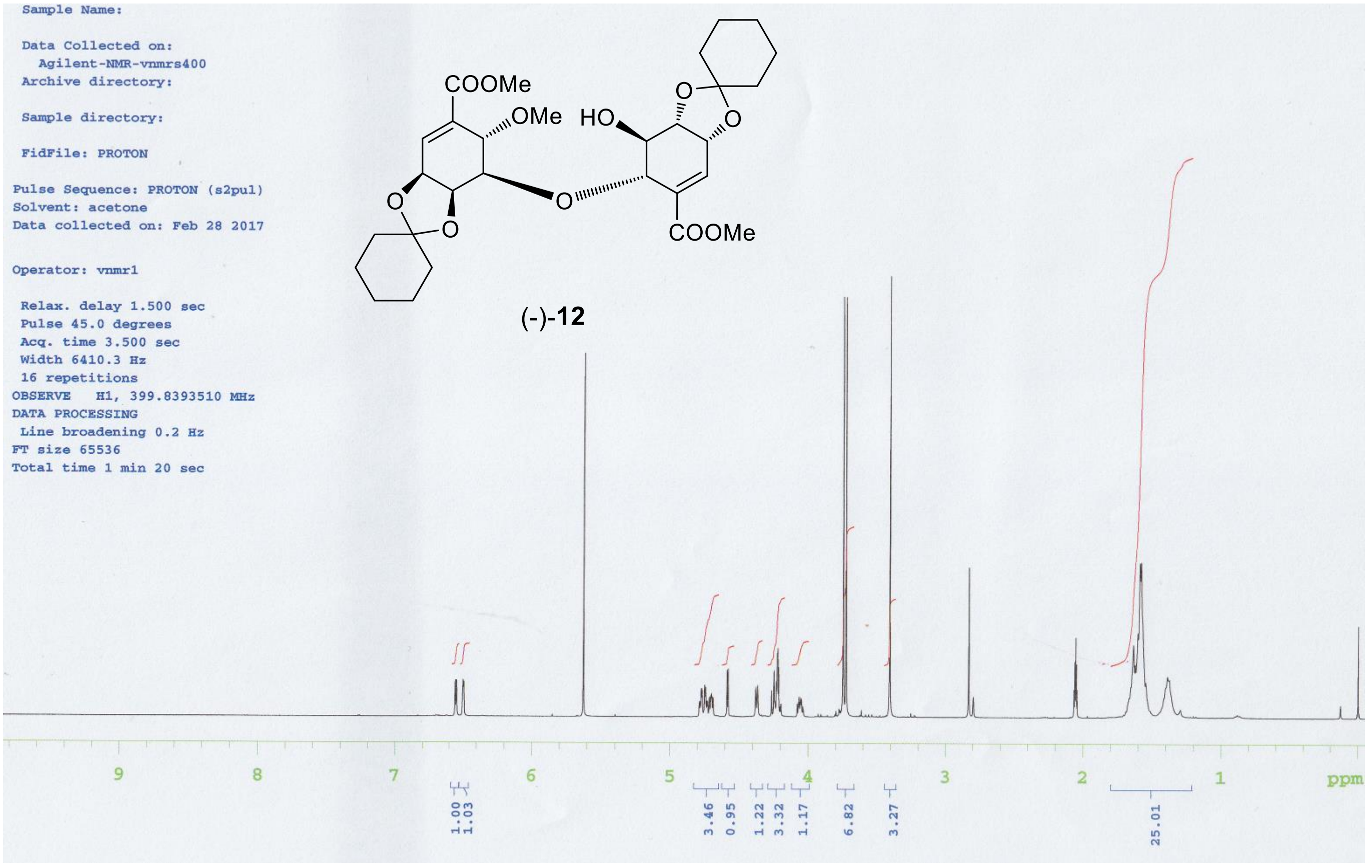

INDEX	FREQUENCY	PPM	HEIGHT	INDEX	FREQUENCY	PPM	HEIGHT	INDEX	FREQUENCY	PPM	HEIGHT
1	2622.0	6.558	20.6	36	1692.7	4.234	21.7	71	641.1	1.603	50.7
2	2620.8	6.555	21.9	37	1689.6	4.226	38.5	72	635.2	1.589	92.9
3	2618.4	6.549	21.8	38	1688.1	4.222	41.6	73	631.7	1.580	93.2
4	2617.1	6.545	21.3	39	1685.5	4.215	30.1	74	618.0	1.546	21.2
5	2598.5	6.499	22.2	40	1681.4	4.205	5.9	75	559.7	1.400	18.6
6	2597.5	6.496	19.1	41	1678.9	4.199	8.1	76	554.8	1.388	24.9
7	2596.0	6.492	21.1	42	1631.9	4.081	7.2	77	548.9	1.373	22.9
8	2249.7	5.626	218.3	43	1628.6	4.073	8.2	78	522.1	1.306	3.2
9	1914.8	4.789	7.8	44	1626.4	4.068	12.0	79	516.8	1.293	5.1
10	1913.8	4.786	8.5	45	1625.3	4.065	9.2	80	51.4	0.129	7.7
11	1912.0	4.782	8.5	46	1622.9	4.059	9.2	81	-0.0	-0.000	55.7
12	1911.1	4.780	8.9	47	1621.5	4.055	11.0				
13	1909.1	4.775	16.3	48	1619.8	4.051	10.2				
14	1907.9	4.772	17.5	49	1616.3	4.042	6.2				
15	1906.4	4.768	17.2	50	1614.3	4.037	4.3				
16	1905.2	4.765	16.1	51	1519.8	3.801	3.4				
17	1898.3	4.748	19.2	52	1510.6	3.778	4.7				
18	1896.0	4.742	17.8	53	1508.7	3.773	3.9				
19	1892.7	4.734	9.7	54	1507.1	3.769	3.7				
20	1890.7	4.729	9.4	55	1498.5	3.748	252.8				
21	1885.2	4.715	10.9	56	1490.3	3.727	252.4				
22	1884.3	4.713	11.4	57	1445.3	3.615	3.5				
23	1881.5	4.706	11.9	58	1363.5	3.410	265.2				
24	1880.4	4.703	13.0	59	1132.1	2.831	90.4				
25	1879.2	4.700	13.5	60	1118.6	2.798	12.3				
26	1878.2	4.697	12.4	61	825.5	2.065	17.1				
27	1875.3	4.690	11.7	62	823.4	2.059	33.8				
28	1874.5	4.688	11.3	63	821.2	2.054	48.4				
29	1834.4	4.588	28.4	64	819.1	2.049	32.5				
30	1831.1	4.579	29.2	65	816.7	2.043	17.0				
31	1751.6	4.381	17.1	66	674.3	1.686	4.2				
32	1746.2	4.367	18.5	67	665.3	1.664	12.2				
33	1705.9	4.266	15.7	68	660.6	1.652	18.8				
34	1699.2	4.250	27.2	69	653.8	1.635	43.7				
35	1696.3	4.242	9.0	70	648.7	1.622	32.1				

Figure S4: ^{13}C-NMR Spectrum of (-)-**12** in acetone-d$_6$ (100 MHz)

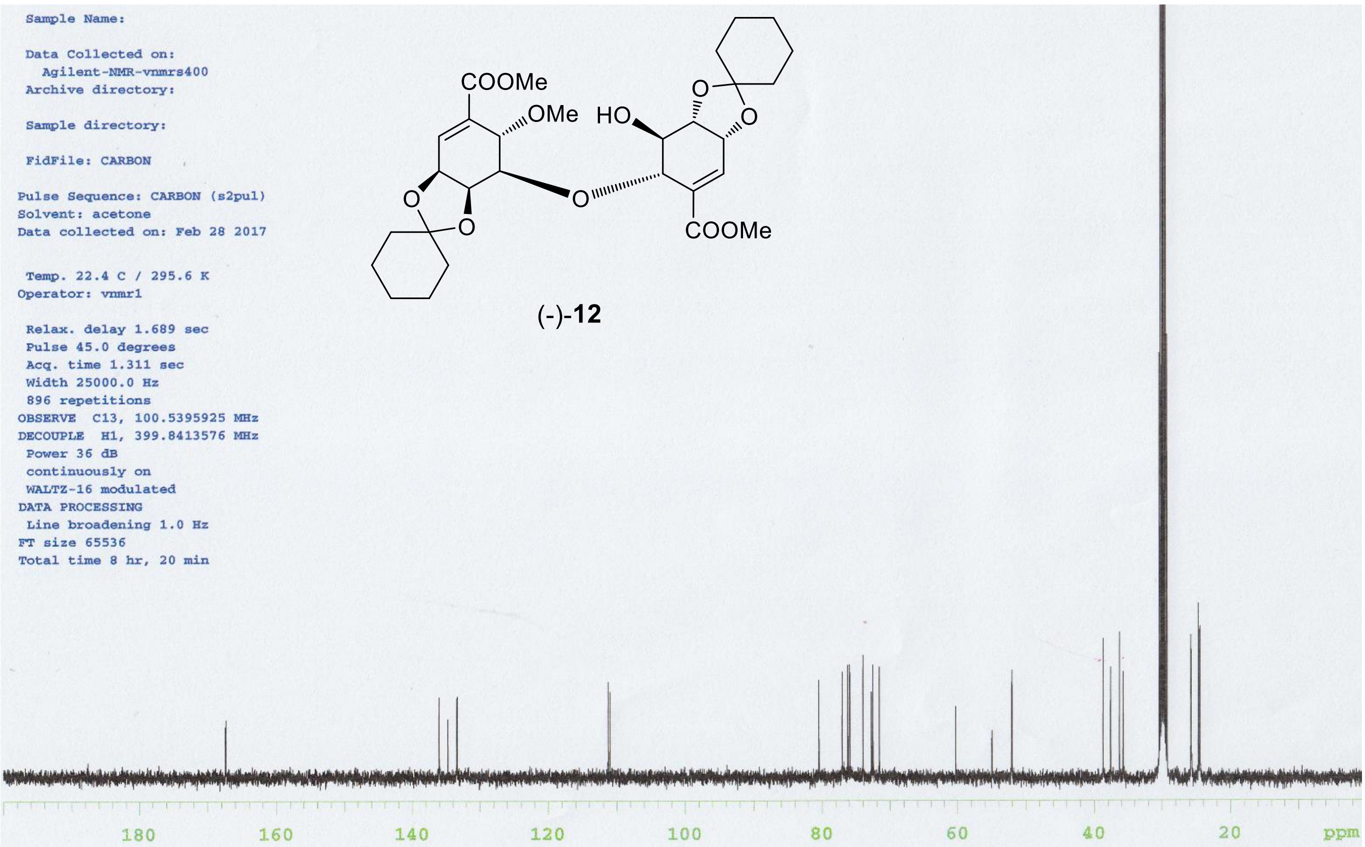

NDEX	FREQUENCY	PPM	HEIGHT	INDEX	FREQUENCY	PPM	HEIGHT
1	16833.6	167.432	9.3	36	2464.4	24.511	28.7
2	16829.8	167.394	10.9	37	2460.5	24.473	29.0
3	13681.1	136.077	15.2				
4	13549.1	134.764	11.2				
5	13414.1	133.421	15.0				
6	13410.3	133.383	15.3				
7	11194.7	111.346	18.5				
8	11169.5	111.096	16.3				
9	8091.0	80.476	18.7				
10	7746.2	77.046	20.1				
11	7661.5	76.204	21.4				
12	7632.5	75.916	21.7				
13	7437.2	73.973	23.4				
14	7315.9	72.766	16.5				
15	7292.2	72.531	21.7				
16	7197.6	71.590	21.1				
17	6066.2	60.336	13.3				
18	5529.1	54.994	8.9				
19	5239.9	52.118	18.3				
20	5232.3	52.042	21.0				
21	3894.9	38.740	26.5				
22	3783.5	37.632	21.2				
23	3649.2	36.296	27.8				
24	3594.3	35.750	20.1				
25	3060.2	30.438	82.5				
26	3041.1	30.248	264.7				
27	3022.1	30.059	484.7				
28	3002.2	29.861	587.1				
29	2983.2	29.672	523.4				
30	2964.1	29.482	230.4				
31	2944.3	29.285	86.6				
32	2593.3	25.794	27.1				
33	2584.9	25.710	24.8				
34	2485.0	24.716	33.4				
35	2482.7	24.694	32.1				

Figure S5: ^{1}H- NMR Spectrum of (+)-**12** in acetone-d$_6$ (600 MHz)

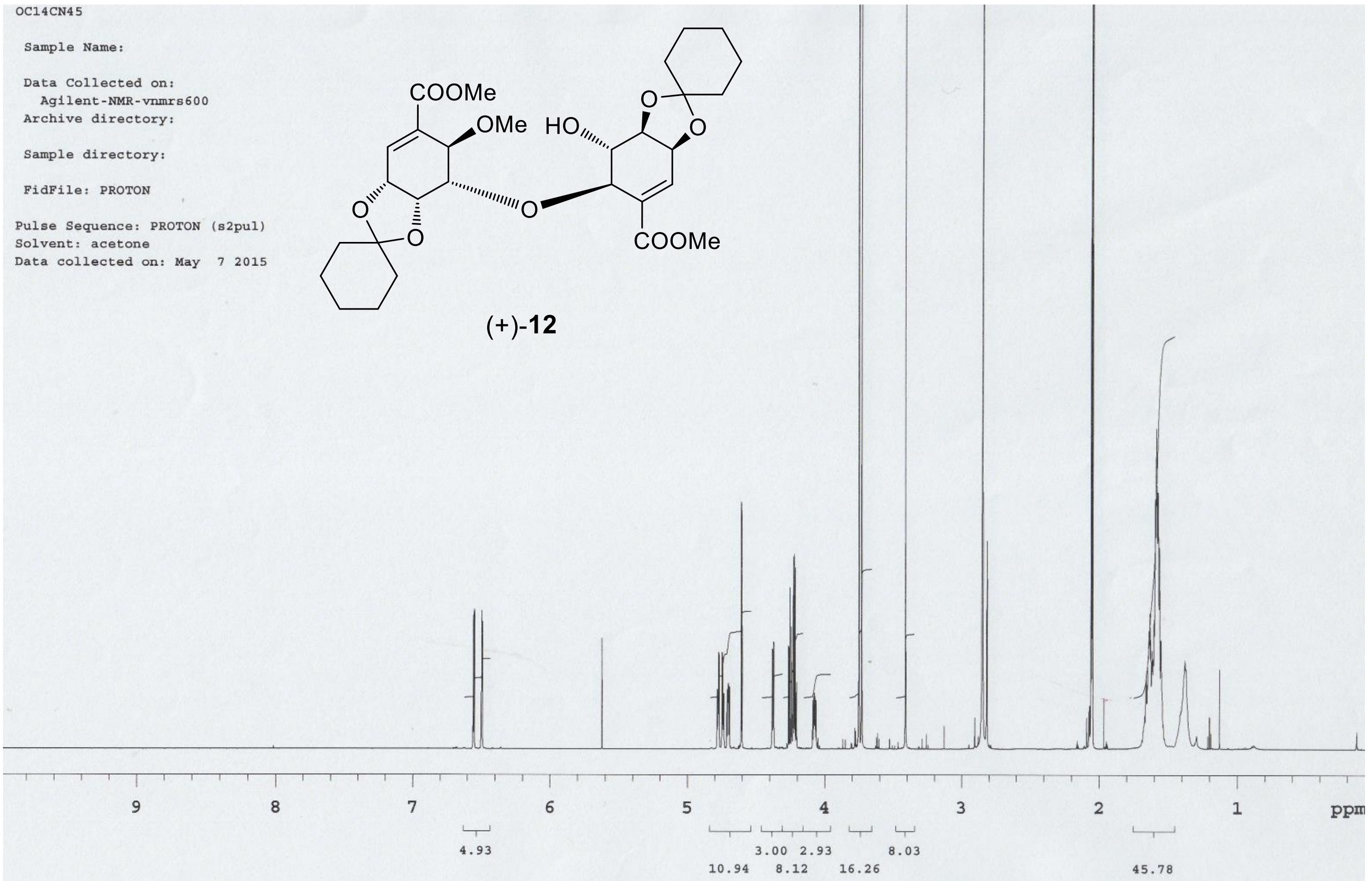

OC14CN45

INDEX	FREQUENCY	PPM	HEIGHT	INDEX	FREQUENCY	PPM	HEIGHT	INDEX	FREQUENCY	PPM	HEIGHT
1	3934.4	6.558	26.0	51	2531.2	4.219	38.0	101	956.6	1.595	48.7
2	3933.0	6.556	26.6	52	2528.3	4.214	35.4	102	951.9	1.587	62.4
3	3930.6	6.552	27.3	53	2524.4	4.208	11.1	103	949.6	1.583	49.5
4	3929.4	6.550	27.0	54	2521.8	4.204	13.0	104	945.2	1.576	50.0
5	3898.6	6.499	16.2	55	2452.0	4.087	9.6	105	939.3	1.566	39.3
6	3897.7	6.497	26.9	56	2448.4	4.081	10.2	106	932.0	1.554	21.3
7	3896.9	6.496	18.3	57	2446.4	4.078	10.7	107	927.0	1.545	11.1
8	3896.0	6.494	17.5	58	2445.2	4.076	11.0	108	921.4	1.536	5.0
9	3894.8	6.492	24.8	59	2442.9	4.072	10.4	109	846.9	1.412	7.0
10	3893.9	6.491	15.0	60	2441.7	4.070	10.1	110	844.2	1.407	7.5
11	3373.1	5.623	21.6	61	2439.6	4.067	9.7	111	827.5	1.379	17.4
12	2869.2	4.783	10.9	62	2436.4	4.061	9.6	112	823.4	1.373	15.7
13	2868.1	4.781	11.7	63	2266.5	3.778	4.2	113	775.6	1.293	2.6
14	2866.6	4.778	11.0	64	2263.6	3.773	3.6	114	727.1	1.212	2.6
15	2865.1	4.776	11.0	65	2250.7	3.752	13.3	115	720.1	1.200	6.4
16	2863.7	4.774	18.3	66	2248.3	3.748	354.0	116	713.1	1.189	3.1
17	2862.2	4.771	18.9	67	2236.6	3.728	418.9	117	677.0	1.128	15.6
18	2860.7	4.769	18.7	68	2167.6	3.613	3.1	118	77.2	0.129	3.5
19	2859.6	4.767	18.3	69	2045.9	3.410	368.3	119	0.0	0.000	91.3
20	2847.5	4.747	17.3	70	1954.9	3.259	3.0				
21	2846.9	4.746	18.9	71	1875.7	3.127	4.6				
22	2844.9	4.742	17.3	72	1741.0	2.902	6.2				
23	2844.0	4.741	18.0	73	1709.0	2.849	2887.4				
24	2841.9	4.737	10.9	74	1693.1	2.822	4.2				
25	2841.1	4.736	10.9	75	1689.0	2.816	40.6				
26	2839.0	4.732	10.9	76	1254.2	2.091	6.2				
27	2838.4	4.732	10.4	77	1253.3	2.089	3.4				
28	2825.5	4.710	11.1	78	1252.4	2.088	3.2				
29	2824.6	4.709	11.7	79	1245.6	2.076	3.0				
30	2822.0	4.704	11.7	80	1244.8	2.075	7.5				
31	2820.8	4.702	12.7	81	1242.4	2.071	8.5				
32	2819.6	4.700	13.0	82	1241.2	2.069	3.3				
33	2818.5	4.698	12.4	83	1240.4	2.068	7.9				
34	2815.8	4.694	12.2	84	1236.8	2.062	99.3				
35	2814.7	4.692	12.2	85	1234.8	2.058	182.3				
36	2763.0	4.606	48.1	86	1232.4	2.054	295.5				
37	2759.5	4.600	46.4	87	1230.4	2.051	180.6				
38	2627.7	4.380	19.6	88	1228.0	2.047	98.3				
39	2626.6	4.378	12.7	89	1180.2	1.967	10.0				
40	2623.3	4.373	12.4	90	1008.0	1.680	3.6				
41	2622.2	4.371	20.9	91	1001.8	1.670	6.6				
42	2621.0	4.369	12.9	92	996.5	1.661	13.6				
43	2557.3	4.263	20.1	93	988.6	1.648	17.3				
44	2550.9	4.252	31.5	94	987.1	1.645	17.8				
45	2544.4	4.241	23.9	95	984.2	1.641	23.0				
46	2542.6	4.238	13.2	96	980.1	1.634	26.4				
47	2541.5	4.236	6.8	97	976.6	1.628	21.3				
48	2537.1	4.229	22.6	98	970.7	1.618	19.2				
49	2535.9	4.227	37.5	99	965.1	1.609	17.6				
50	2534.7	4.225	22.5	100	961.9	1.603	25.3				

Figure S6: ^{13}C-NMR Spectrum of (+)-**12** in acetone-d_6 (125 MHz)

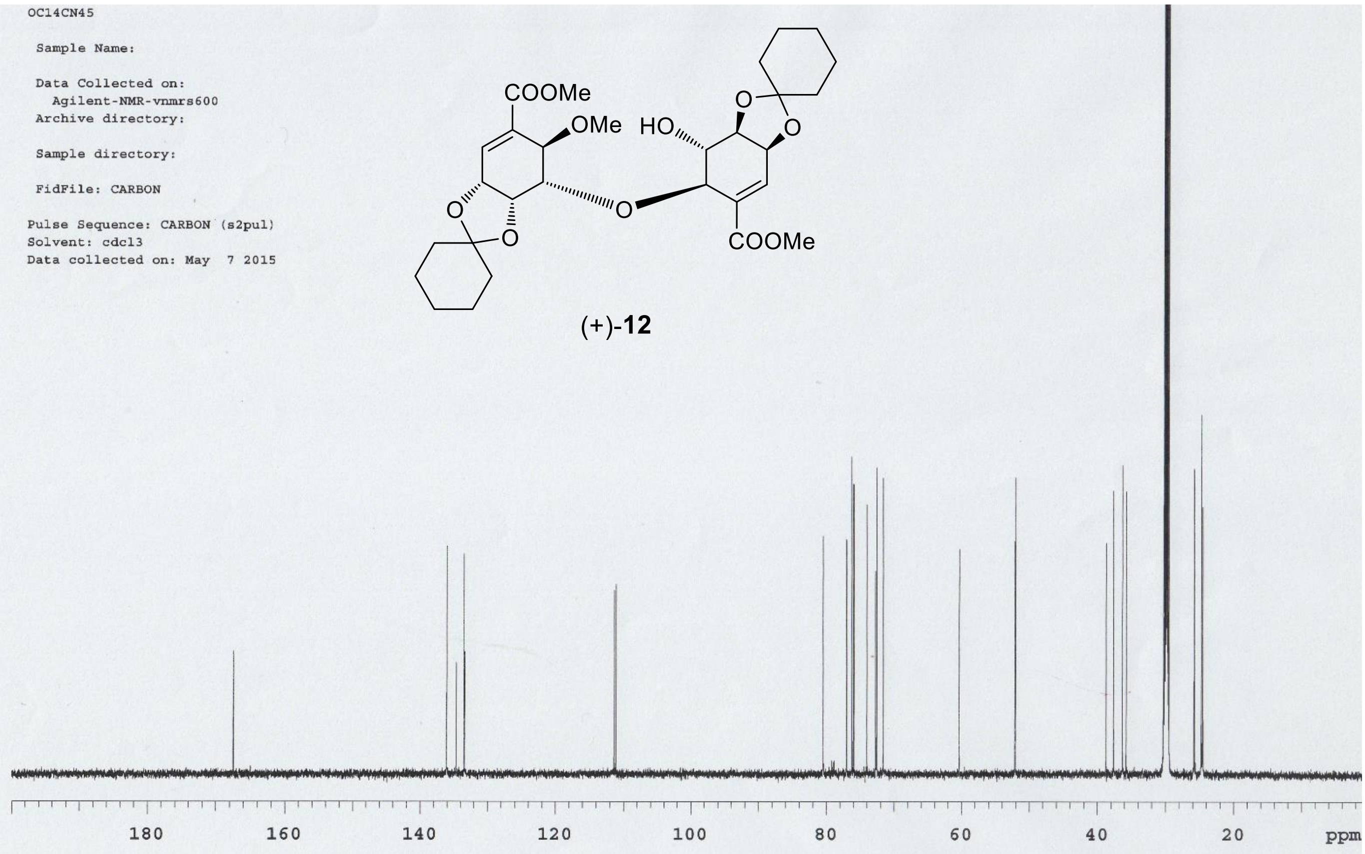

INDEX	FREQUENCY	PPM	HEIGHT
1	25257.9	167.443	24.7
2	25251.0	167.397	23.9
3	20526.5	136.077	45.3
4	20313.8	134.667	22.3
5	20135.8	133.487	43.8
6	20119.6	133.380	24.4
7	16796.2	111.348	36.6
8	16758.1	111.095	37.8
9	12137.7	80.465	47.4
10	11615.2	77.001	46.6
11	11492.6	76.189	62.9
12	11452.2	75.920	57.5
13	11158.6	73.974	53.5
14	10966.7	72.702	40.4
15	10941.2	72.533	60.7
16	10796.7	71.575	58.7
17	9097.5	60.310	44.7
18	7859.4	52.103	46.2
19	7847.9	52.026	58.8
20	5842.3	38.730	45.9
21	5675.8	37.627	56.1
22	5474.7	36.293	61.2
23	5391.4	35.742	56.1
24	4577.6	30.347	11.1
25	4559.1	30.224	243.5
26	4540.6	30.101	940.2
27	4521.0	29.971	2041.2
28	4501.3	29.841	2128.5
29	4482.8	29.718	1478.4
30	4463.2	29.588	959.3
31	4443.5	29.458	341.7
32	3889.8	25.787	60.6
33	3878.3	25.710	60.2
34	3725.7	24.699	71.2
35	3723.4	24.684	69.1
36	3696.8	24.507	53.1
37	3691.0	24.469	50.7

Figure S7: ^{1}H- NMR Spectrum of (-)-**16** in C_6D_6 (300 MHz)

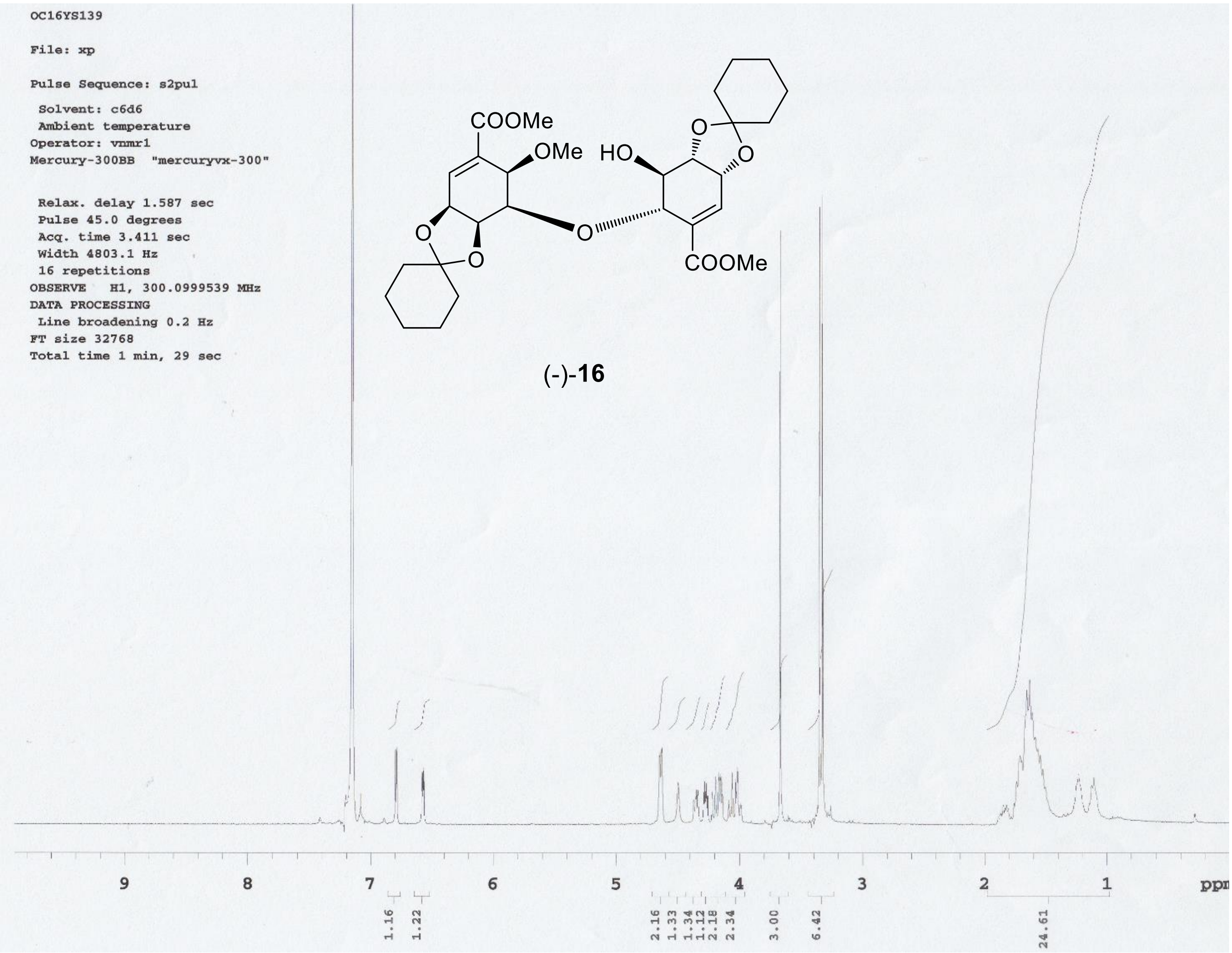

INDEX	FREQUENCY	PPM	HEIGHT	INDEX	FREQUENCY	PPM	HEIGHT
1	2161.8	7.204	17.5	42	554.5	1.848	12.1
2	2145.7	7.150	1278.3	43	549.8	1.832	10.7
3	2125.5	7.083	18.1	44	546.8	1.822	12.1
4	2039.3	6.795	41.4	45	542.4	1.808	10.4
5	2036.4	6.786	42.6	46	527.2	1.757	13.2
6	1975.1	6.581	29.4	47	521.6	1.738	24.7
7	1973.6	6.577	29.7	48	513.1	1.710	38.3
8	1971.6	6.570	31.6	49	508.7	1.695	35.7
9	1969.8	6.564	28.0	50	497.0	1.656	72.5
10	1394.3	4.646	38.6	51	489.4	1.631	77.9
11	1392.9	4.641	39.9	52	483.5	1.611	60.7
12	1389.7	4.631	41.4	53	476.8	1.589	47.9
13	1388.5	4.627	42.6	54	469.1	1.563	41.6
14	1348.9	4.495	24.2	55	463.6	1.545	38.7
15	1309.9	4.365	15.1	56	456.8	1.522	31.0
16	1306.1	4.352	16.6	57	450.7	1.502	21.6
17	1304.1	4.345	20.3	58	370.1	1.233	28.4
18	1299.7	4.331	20.0	59	337.5	1.125	22.6
19	1300.2	4.333	20.0	60	332.2	1.107	26.6
20	1286.8	4.288	21.4				
21	1283.2	4.276	25.7				
22	1280.3	4.266	23.8				
23	1276.8	4.255	20.0				
24	1265.7	4.217	19.3				
25	1257.2	4.189	27.1				
26	1250.1	4.166	26.5				
27	1248.9	4.162	29.6				
28	1245.7	4.151	28.8				
29	1244.5	4.147	27.4				
30	1241.3	4.136	24.3				
31	1225.8	4.085	15.1				
32	1217.6	4.057	29.3				
33	1209.1	4.029	26.1				
34	1204.7	4.014	32.1				
35	1196.8	3.988	13.0				
36	1101.2	3.669	314.4				
37	1010.3	3.367	12.5				
38	1005.3	3.350	326.6				
39	998.3	3.327	333.3				
40	985.1	3.283	8.7				
41	977.8	3.258	12.1				

Figure S8: ^{13}C-NMR Spectrum of (-)-**16** in C_6D_6 (75.5 MHz)

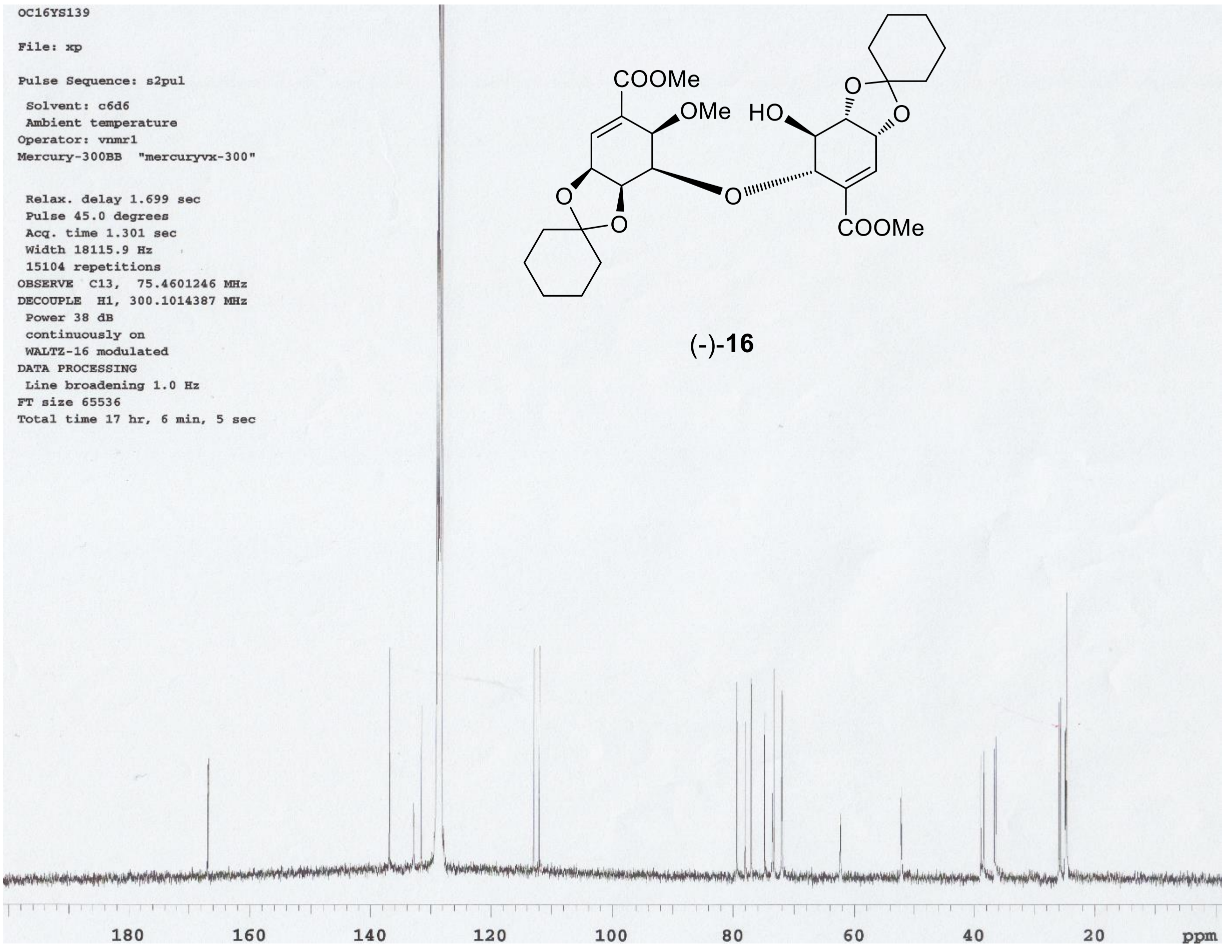

INDEX	FREQUENCY	PPM	HEIGHT
1	12594.9	166.908	23.6
2	12586.1	166.791	24.9
3	10328.7	136.877	47.6
4	10027.4	132.884	15.7
5	9930.1	131.595	35.6
6	9745.5	129.148	133.0
7	9729.5	128.935	2049.1
8	9705.7	128.620	2051.6
9	9694.6	128.473	77.1
10	9681.4	128.298	2060.4
11	9650.4	127.887	11.2
12	8523.1	112.949	47.4
13	8453.5	112.026	48.0
14	5991.6	79.401	40.5
15	5890.4	78.060	32.5
16	5815.8	77.071	41.4
17	5649.4	74.866	34.2
18	5554.3	73.606	17.9
19	5533.8	73.335	43.3
20	5437.6	72.060	21.2
21	5432.1	71.987	38.8
22	4701.8	62.308	13.7
23	3931.1	52.095	19.0
24	2936.5	38.915	25.6
25	2898.9	38.417	26.4
26	2765.1	36.644	26.9
27	2746.3	36.395	29.4
28	1961.8	25.998	36.5
29	1939.7	25.705	37.4
30	1883.3	24.958	30.6
31	1873.9	24.834	31.3
32	1864.5	24.709	58.9

Figure S9: ^{1}H- NMR Spectrum of (+)-**16** in C_6D_6 (600 MHz)

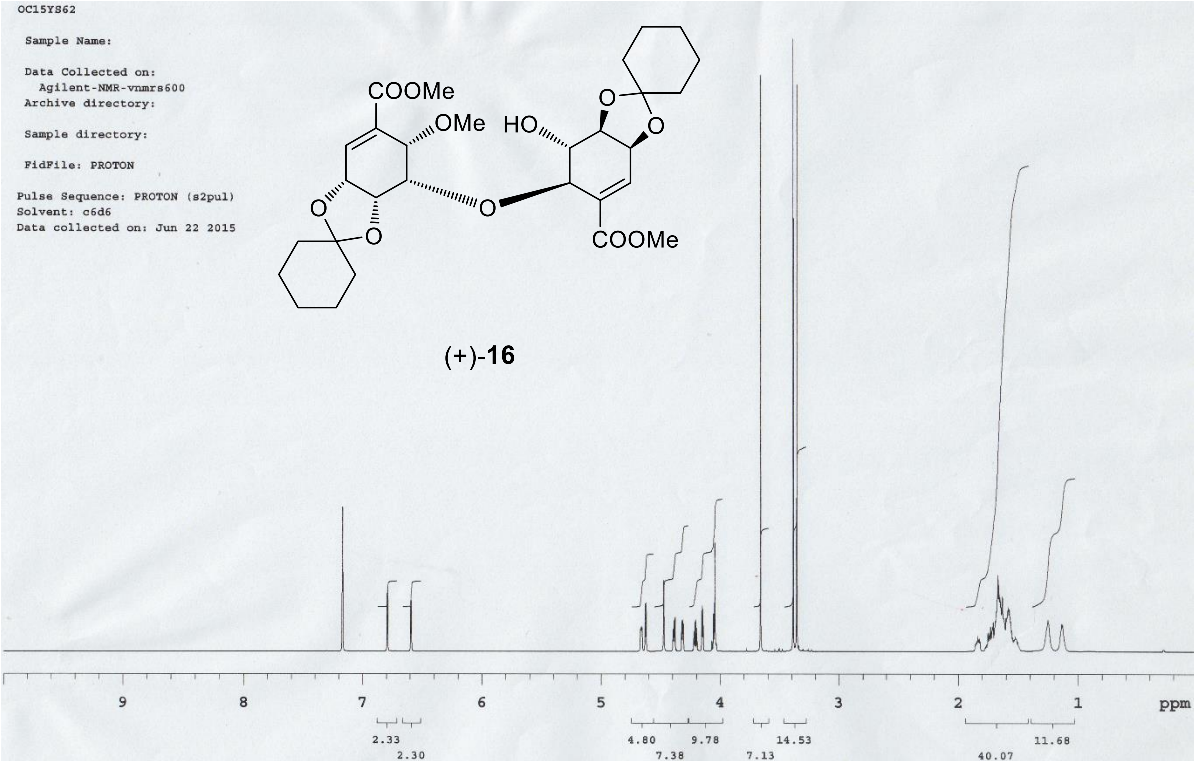

NDEX	FREQUENCY	PPM	HEIGHT	INDEX	FREQUENCY	PPM	HEIGHT
1	4300.9	7.169	125.6	51	1103.6	1.840	14.0
2	4076.4	6.795	50.5	52	1099.2	1.832	10.5
3	4075.9	6.794	54.7	53	1095.4	1.826	11.4
4	4073.2	6.790	55.3	54	1091.6	1.820	9.3
5	4072.6	6.789	52.0	55	1051.1	1.752	16.7
6	3956.4	6.595	39.2	56	1046.7	1.745	14.5
7	3955.0	6.593	37.0	57	1042.9	1.738	16.5
8	3952.9	6.589	44.5	58	1038.2	1.731	21.9
9	3951.4	6.587	35.8	59	1032.9	1.722	17.1
10	2799.7	4.667	20.0	60	1029.7	1.716	21.6
11	2798.8	4.666	18.8	61	1025.3	1.709	26.0
12	2797.1	4.663	21.6	62	1020.0	1.700	17.4
13	2793.8	4.657	22.5	63	1015.6	1.693	27.4
14	2792.1	4.654	19.4	64	1010.3	1.684	35.1
15	2790.9	4.652	21.2	65	1002.7	1.671	66.4
16	2775.6	4.627	41.8	66	998.0	1.664	58.1
17	2770.9	4.619	42.7	67	991.8	1.653	47.2
18	2682.9	4.472	62.0	68	987.7	1.646	39.2
19	2636.5	4.395	19.3	69	980.1	1.634	48.0
20	2635.7	4.394	20.2	70	975.1	1.625	29.3
21	2632.1	4.388	21.9	71	972.7	1.622	28.9
22	2629.5	4.383	29.7	72	961.9	1.603	29.5
23	2625.7	4.377	30.6	73	957.5	1.596	30.0
24	2592.8	4.322	25.5	74	953.1	1.589	35.8
25	2592.2	4.321	26.3	75	949.6	1.583	38.9
26	2589.6	4.317	27.8	76	945.2	1.576	37.0
27	2589.0	4.316	28.0	77	941.1	1.569	30.3
28	2587.0	4.312	27.1	78	936.9	1.562	26.2
29	2586.4	4.311	26.2	79	932.3	1.554	18.9
30	2583.7	4.307	24.4	80	927.9	1.547	12.6
31	2531.8	4.220	18.0	81	922.3	1.537	10.1
32	2529.7	4.217	12.5	82	918.5	1.531	10.1
33	2525.0	4.209	25.9	83	914.9	1.525	13.3
34	2523.0	4.206	27.9	84	910.5	1.518	10.8
35	2516.2	4.194	20.8	85	908.2	1.514	11.6
36	2492.8	4.155	35.5	86	902.9	1.505	8.7
37	2489.8	4.150	40.1	87	750.9	1.252	27.8
38	2488.4	4.148	37.4	88	747.7	1.246	25.4
39	2485.1	4.143	32.0	89	682.0	1.137	24.5
40	2441.7	4.070	9.4	90	677.5	1.129	23.2
41	2440.8	4.069	9.0	91	0.0	0.000	74.3
42	2433.5	4.057	32.7				
43	2432.6	4.055	33.3				
44	2426.1	4.044	94.6				
45	2419.4	4.033	7.9				
46	2195.2	3.659	500.7				
47	2031.5	3.386	532.5				
48	2013.3	3.356	492.6				
49	1111.8	1.853	8.0				
50	1108.3	1.848	8.7				

Figure S10: ^{13}C-NMR Spectrum of (+)-**16** in C_6D_6 (150 MHz)

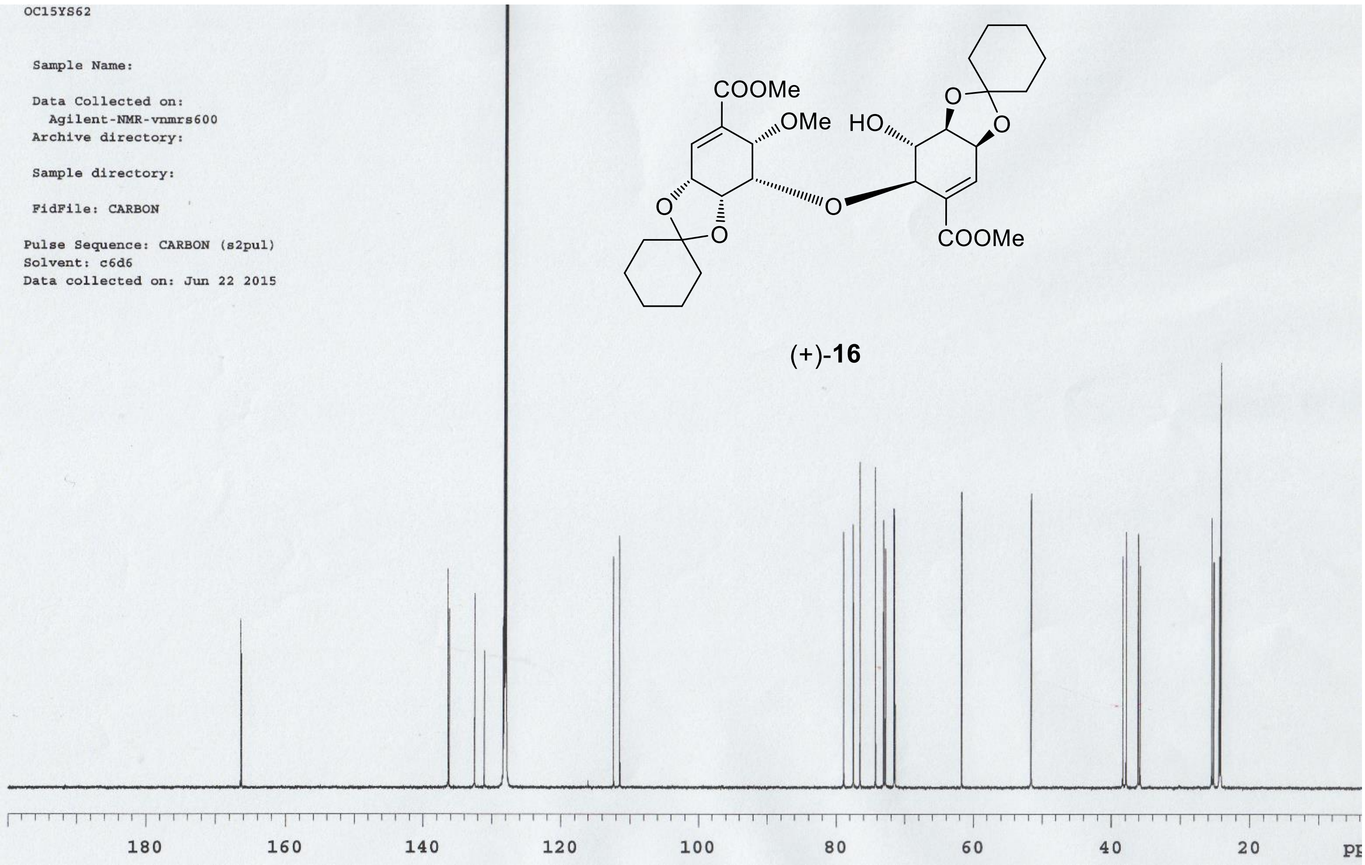

OC15YS62

INDEX	FREQUENCY	PPM	HEIGHT
1	25094.9	166.364	32.6
2	25075.3	166.233	26.0
3	20569.3	136.362	42.4
4	20542.7	136.185	34.7
5	19980.9	132.461	37.6
6	19768.2	131.051	26.5
7	19357.9	128.330	33.1
8	19339.4	128.208	701.4
9	19326.6	128.124	21.4
10	19315.1	128.047	729.3
11	19302.4	127.963	26.0
12	19290.8	127.886	738.7
13	16947.7	112.352	44.8
14	16811.3	111.448	48.8
15	11897.2	78.871	49.5
16	11683.4	77.453	51.1
17	11535.4	76.473	63.1
18	11195.6	74.220	62.1
19	11019.9	73.055	51.8
20	10980.5	72.794	46.3
21	10791.0	71.537	54.1
22	10772.5	71.415	52.7
23	9305.5	61.690	57.4
24	7783.1	51.597	55.7
25	7777.4	51.559	57.0
26	5782.2	38.332	44.9
27	5708.2	37.842	49.6
28	5443.5	36.087	49.2
29	5399.5	35.795	43.0
30	3836.7	25.435	52.3
31	3795.0	25.159	43.8
32	3677.1	24.377	44.8
33	3658.6	24.254	45.0
34	3640.1	24.132	82.3
35	0.0	0.000	10.6

Figure S11: ^{1}H- NMR Spectrum of (-)-**19** in $CDCl_3$ (600 MHz)

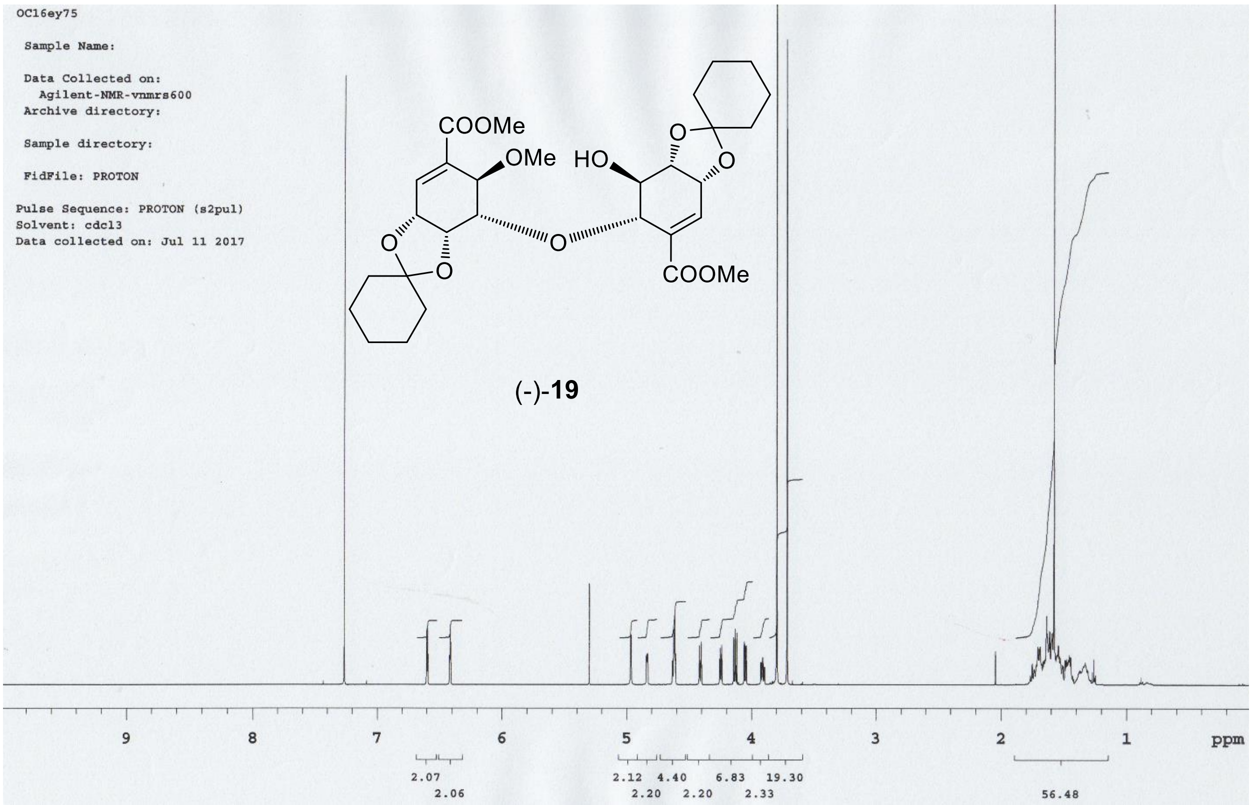

OC16ey75

INDEX	FREQUENCY	PPM	HEIGHT	INDEX	FREQUENCY	PPM	HEIGHT	INDEX	FREQUENCY	PPM	HEIGHT
1	4357.3	7.263	397.1	51	2355.1	3.926	14.6	101	854.2	1.424	4.2
2	3960.5	6.602	35.3	52	2352.5	3.922	14.4	102	829.5	1.383	7.7
3	3958.8	6.599	37.1	53	2349.6	3.917	5.5	103	826.3	1.377	8.9
4	3956.4	6.595	38.0	54	2347.5	3.913	15.4	104	822.5	1.371	9.6
5	3954.4	6.592	35.4	55	2345.7	3.910	17.9	105	818.7	1.365	9.0
6	3852.0	6.421	19.4	56	2345.2	3.909	17.9	106	805.5	1.343	12.4
7	3850.5	6.419	29.0	57	2343.1	3.906	14.1	107	801.4	1.336	13.3
8	3848.7	6.416	39.2	58	2338.4	3.898	11.7	108	799.6	1.333	13.5
9	3847.6	6.414	28.6	59	2335.8	3.894	11.7	109	797.9	1.330	14.7
10	3845.8	6.411	19.3	60	2277.4	3.796	584.0	110	794.3	1.324	12.3
11	3180.3	5.301	65.9	61	2276.5	3.795	545.9	111	794.9	1.325	12.3
12	2980.7	4.969	33.0	62	2227.8	3.714	421.1	112	788.5	1.314	9.9
13	2978.1	4.964	32.4	63	1227.5	2.046	22.1	113	784.9	1.308	9.4
14	2905.3	4.843	16.0	64	1060.2	1.767	6.4	114	762.9	1.272	8.6
15	2903.9	4.841	19.9	65	1050.8	1.752	14.0	115	755.9	1.260	16.8
16	2901.8	4.837	17.8	66	1042.3	1.737	10.3	116	753.0	1.255	4.8
17	2900.3	4.835	18.7	67	1039.9	1.734	9.7	117	748.6	1.248	6.5
18	2898.3	4.831	20.7	68	1032.0	1.720	12.9	118	530.2	0.884	4.9
19	2897.1	4.829	16.4	69	1029.1	1.715	13.6	119	3.2	0.005	11.4
20	2779.4	4.633	15.1	70	1023.8	1.707	25.1	120	-0.0	-0.000	409.2
21	2778.3	4.631	15.7	71	1019.7	1.700	16.5	121	-3.2	-0.005	9.5
22	2775.0	4.626	17.2	72	1013.5	1.690	25.2				
23	2772.7	4.622	37.0	73	1006.5	1.678	14.1				
24	2770.9	4.619	30.8	74	999.7	1.667	15.5				
25	2769.8	4.617	28.1	75	995.6	1.660	15.3				
26	2767.7	4.614	49.5	76	992.4	1.654	16.5				
27	2765.9	4.611	22.9	77	988.9	1.648	16.2				
28	2764.8	4.609	20.5	78	983.0	1.639	44.9				
29	2763.0	4.606	16.6	79	968.6	1.615	35.5				
30	2651.5	4.420	14.3	80	959.2	1.599	29.6				
31	2649.7	4.417	25.7	81	956.3	1.594	34.0				
32	2648.0	4.414	14.1	82	947.8	1.580	920.6				
33	2643.0	4.406	16.1	83	941.3	1.569	20.6				
34	2641.2	4.403	27.8	84	936.4	1.561	18.0				
35	2639.5	4.400	14.5	85	933.1	1.555	19.6				
36	2552.3	4.255	16.2	86	932.0	1.554	19.6				
37	2550.9	4.252	23.9	87	926.1	1.544	25.8				
38	2549.1	4.249	16.0	88	922.0	1.537	18.7				
39	2544.7	4.242	17.7	89	918.2	1.531	17.5				
40	2543.2	4.239	26.1	90	915.5	1.526	15.5				
41	2541.8	4.237	16.5	91	905.8	1.510	13.2				
42	2486.6	4.145	31.1	92	894.4	1.491	16.6				
43	2480.1	4.134	29.9	93	890.3	1.484	15.9				
44	2477.5	4.130	36.4	94	886.8	1.478	16.7				
45	2471.0	4.119	33.9	95	882.7	1.471	15.0				
46	2469.3	4.116	7.1	96	878.6	1.465	15.4				
47	2435.0	4.059	28.1	97	875.3	1.459	19.7				
48	2432.9	4.056	26.7	98	871.8	1.453	18.2				
49	2426.7	4.045	25.4	99	867.1	1.445	18.3				
50	2424.4	4.041	25.7	100	858.9	1.432	5.8				

Figure S12: ^{13}C-NMR Spectrum of (-)-**19** in $CDCl_3$ (150 MHz)

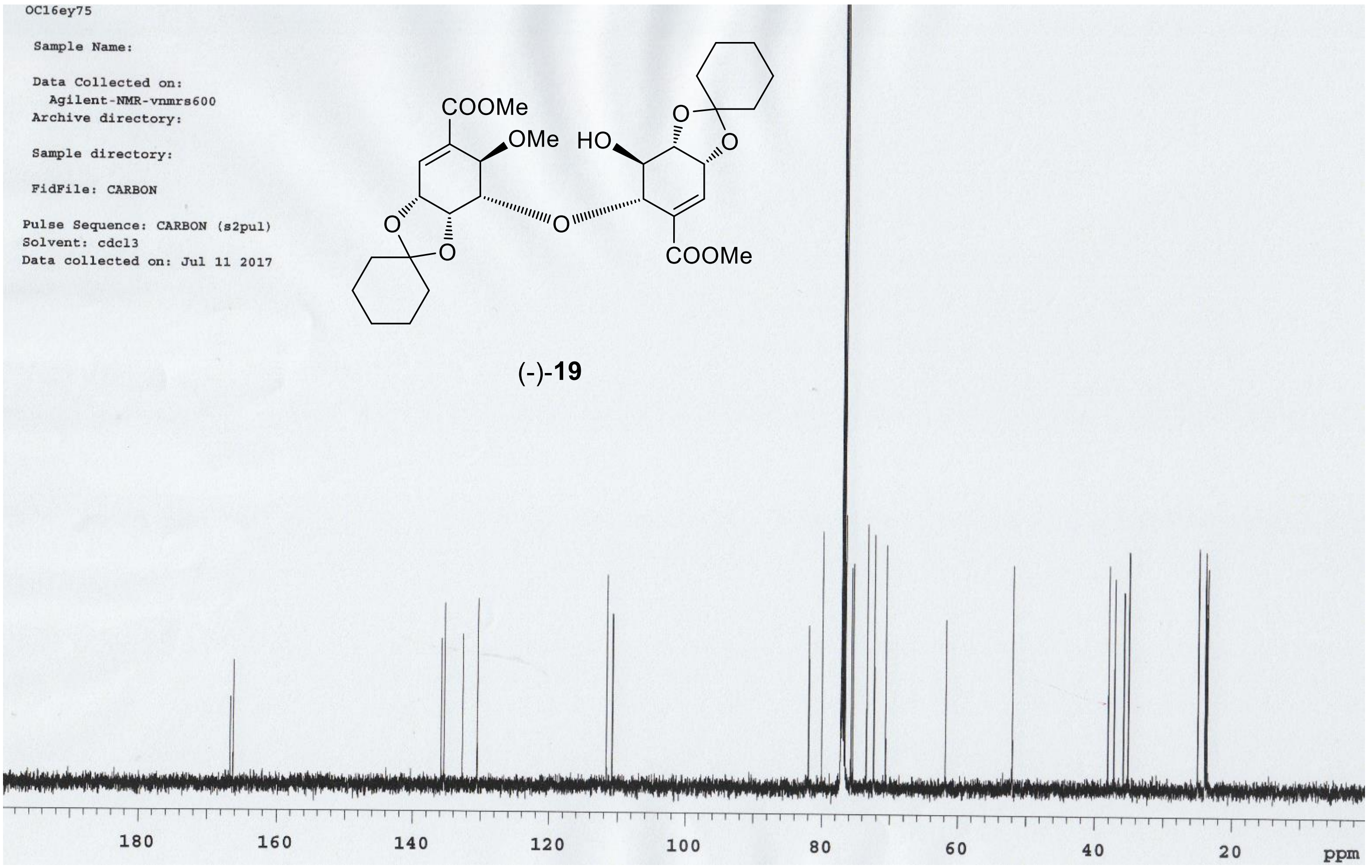

OC16ey75

INDEX	FREQUENCY	PPM	HEIGHT
1	25141.0	166.669	16.9
2	25078.5	166.255	24.0
3	20494.0	135.862	28.6
4	20425.8	135.410	35.5
5	20013.1	132.674	29.4
6	19687.1	130.513	36.4
7	16844.6	111.669	41.3
8	16712.8	110.795	33.6
9	12362.9	81.958	31.6
10	12070.4	80.019	50.0
11	11646.2	77.207	784.7
12	11613.8	76.992	708.7
13	11582.6	76.785	780.7
14	11554.9	76.602	53.3
15	11420.8	75.713	43.1
16	11380.3	75.444	43.8
17	11077.5	73.437	51.5
18	10916.8	72.371	49.4
19	10659.0	70.662	47.5
20	9329.6	61.850	33.0
21	7855.8	52.079	43.7
22	7851.1	52.048	41.5
23	5748.4	38.109	43.8
24	5614.3	37.220	41.2
25	5404.0	35.825	38.6
26	5298.8	35.127	46.5
27	3766.0	24.966	47.3
28	3761.3	24.935	39.6
29	3612.2	23.947	46.5
30	3607.6	23.916	43.1
31	3584.5	23.763	36.5
32	3562.5	23.617	43.3

Figure S13: ^{1}H- NMR Spectrum of (+)-**19** in $CDCl_3$ (400 MHz)

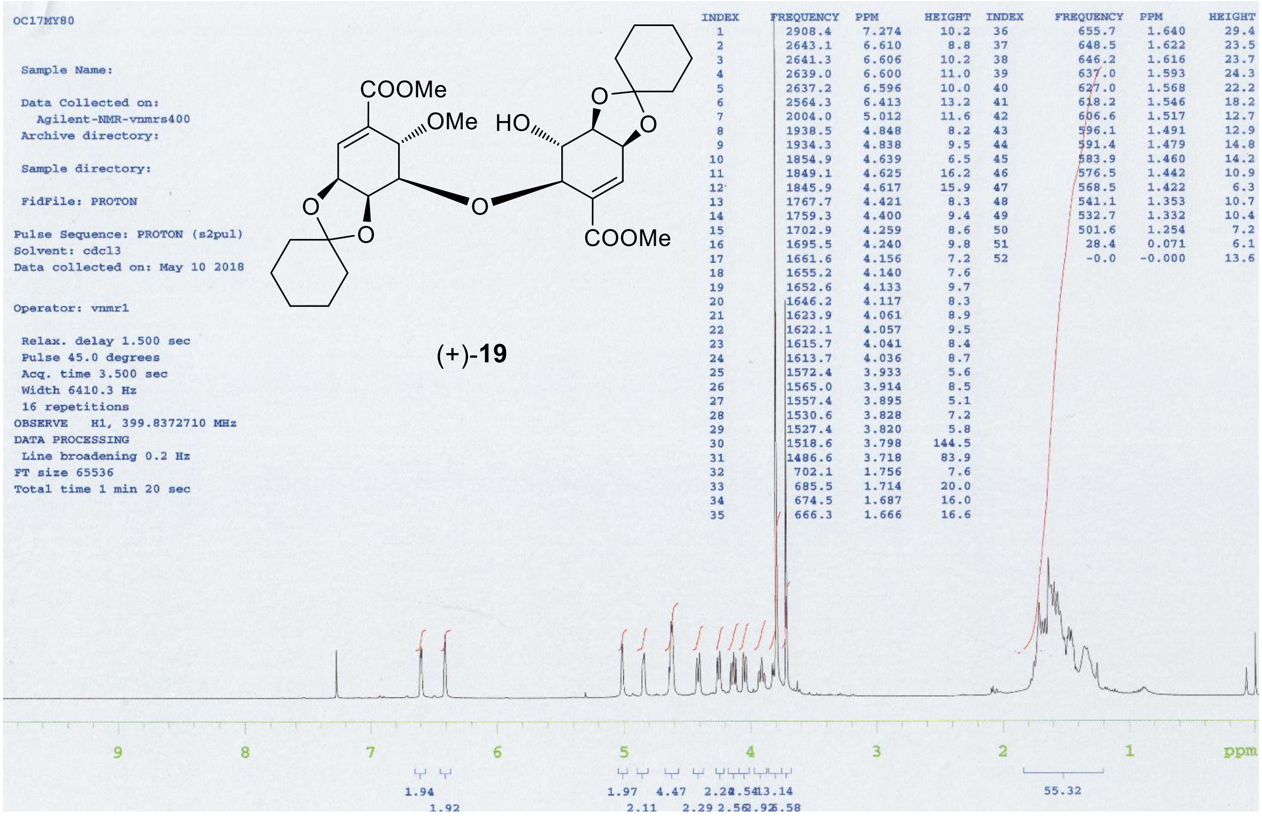

Figure S14: ^{13}C-NMR Spectrum of (+)-**19** in $CDCl_3$ (100 MHz)

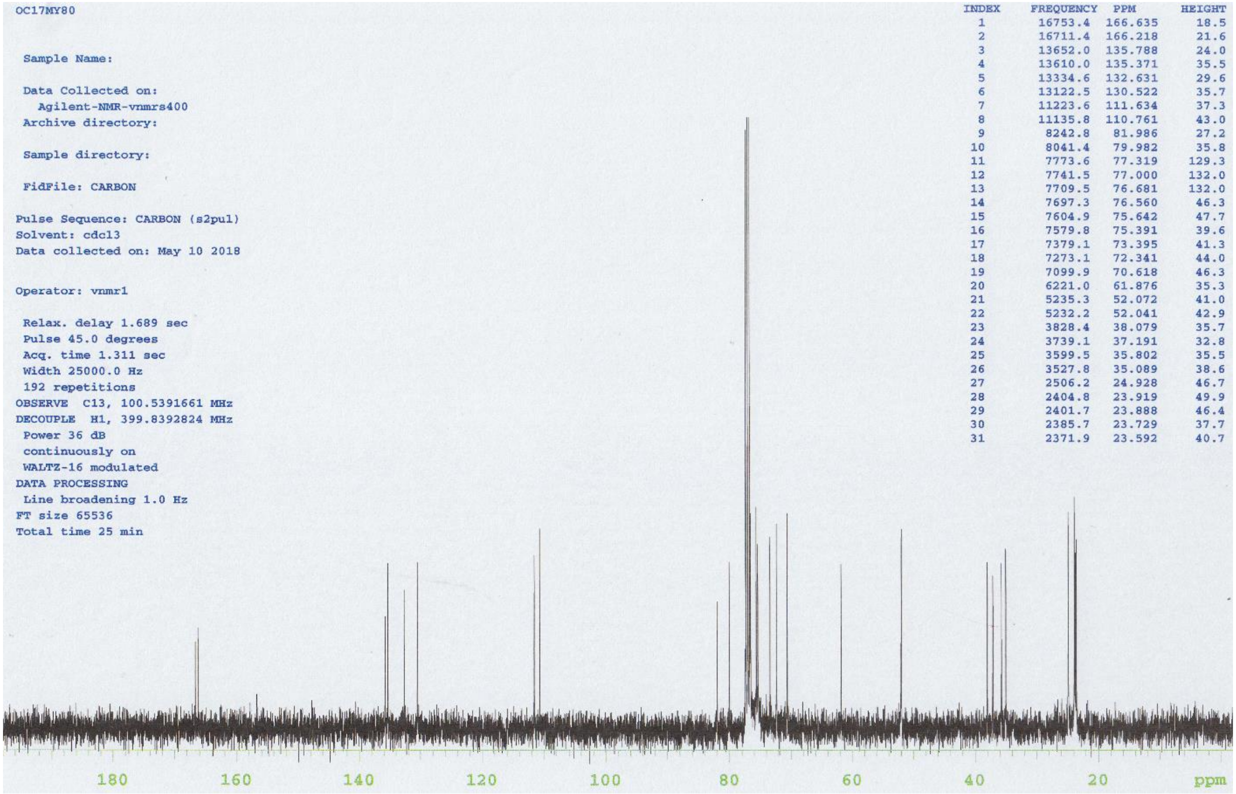

Figure S15: ^{1}H- NMR Spectrum of (-)-**22** in $CDCl_3$ (600 MHz)

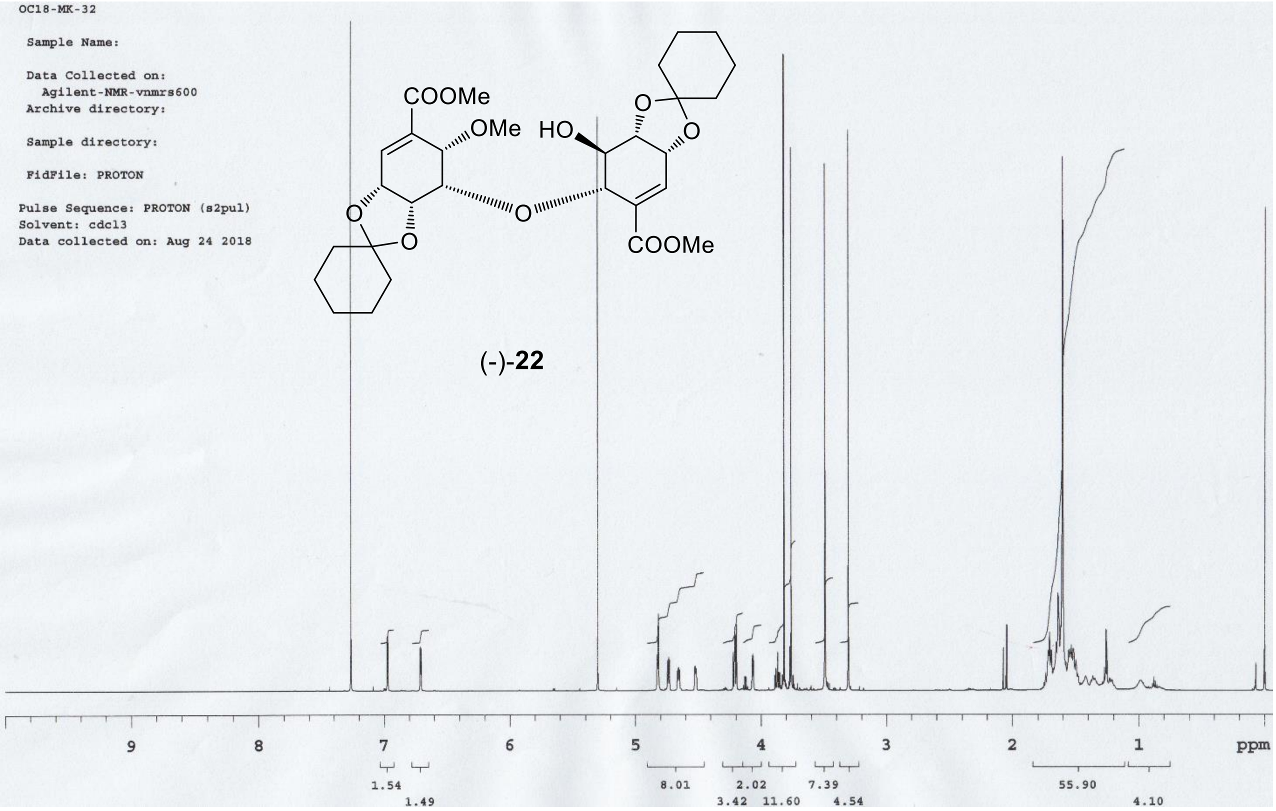

OC18-MK-32

INDEX	FREQUENCY	PPM	HEIGHT	INDEX	FREQUENCY	PPM	HEIGHT
1	4357.9	7.264	454.2	51	2256.0	3.761	368.6
2	4185.9	6.978	36.4	52	2245.7	3.743	10.8
3	4185.0	6.976	36.9	53	2094.3	3.491	357.7
4	4182.1	6.971	37.7	54	1983.1	3.306	380.3
5	4181.5	6.970	33.1	55	1244.2	2.074	29.4
6	4028.3	6.715	29.4	56	1231.9	2.053	10.1
7	4026.3	6.712	30.2	57	1227.5	2.046	45.0
8	4024.5	6.709	29.6	58	1041.7	1.736	12.2
9	4022.5	6.705	28.9	59	1034.4	1.724	18.3
10	3180.3	5.301	389.7	60	1031.4	1.719	27.5
11	2894.8	4.825	24.8	61	1029.1	1.715	26.8
12	2889.5	4.817	52.6	62	1024.4	1.708	39.7
13	2845.5	4.743	20.9	63	1015.9	1.693	27.9
14	2841.7	4.737	21.3	64	1003.9	1.673	15.2
15	2839.6	4.733	23.1	65	991.8	1.653	33.1
16	2835.8	4.727	21.0	66	983.6	1.640	66.2
17	2799.1	4.666	13.4	67	970.7	1.618	44.6
18	2797.9	4.664	13.6	68	962.5	1.604	362.0
19	2794.1	4.658	15.1	69	946.3	1.578	19.7
20	2792.4	4.655	16.0	70	932.3	1.554	28.2
21	2791.5	4.653	14.9	71	928.7	1.548	25.6
22	2788.5	4.648	14.9	72	923.7	1.540	27.7
23	2787.4	4.646	14.1	73	920.5	1.534	31.1
24	2715.2	4.526	16.7	74	916.1	1.527	22.8
25	2714.0	4.524	14.5	75	911.1	1.519	28.8
26	2712.2	4.521	17.0	76	908.5	1.514	28.5
27	2709.3	4.516	15.6	77	898.8	1.498	21.0
28	2708.1	4.514	13.1	78	890.0	1.484	11.3
29	2707.6	4.513	13.3	79	853.3	1.422	10.1
30	2706.4	4.511	14.8	80	826.0	1.377	7.0
31	2532.1	4.221	26.4	81	817.5	1.363	10.2
32	2525.3	4.210	41.0	82	808.1	1.347	8.3
33	2523.0	4.206	39.1	83	804.9	1.342	8.0
34	2515.9	4.194	44.8	84	763.2	1.272	15.8
35	2476.6	4.128	9.7	85	755.9	1.260	41.6
36	2469.6	4.117	9.5	86	752.7	1.255	33.3
37	2442.6	4.072	21.7	87	748.9	1.248	19.9
38	2439.6	4.067	25.3	88	738.0	1.230	8.6
39	2438.5	4.065	24.5	89	735.1	1.225	7.8
40	2435.5	4.060	20.5	90	727.1	1.212	7.5
41	2334.6	3.892	10.5	91	724.8	1.208	6.8
42	2328.7	3.882	15.3	92	591.3	0.986	7.1
43	2320.2	3.868	26.3	93	528.2	0.880	9.6
44	2311.7	3.854	12.6	94	521.1	0.869	6.6
45	2311.1	3.853	12.5	95	42.0	0.070	18.8
46	2297.6	3.830	11.0	96	3.2	0.005	10.2
47	2296.4	3.828	7.4	97	-0.0	-0.000	327.1
48	2290.0	3.817	431.9	98	-3.2	-0.005	12.6
49	2282.9	3.806	7.5				
50	2261.8	3.770	30.0				

Figure S16: ^{13}C-NMR Spectrum of (-)-**22** in $CDCl_3$ (150 MHz)

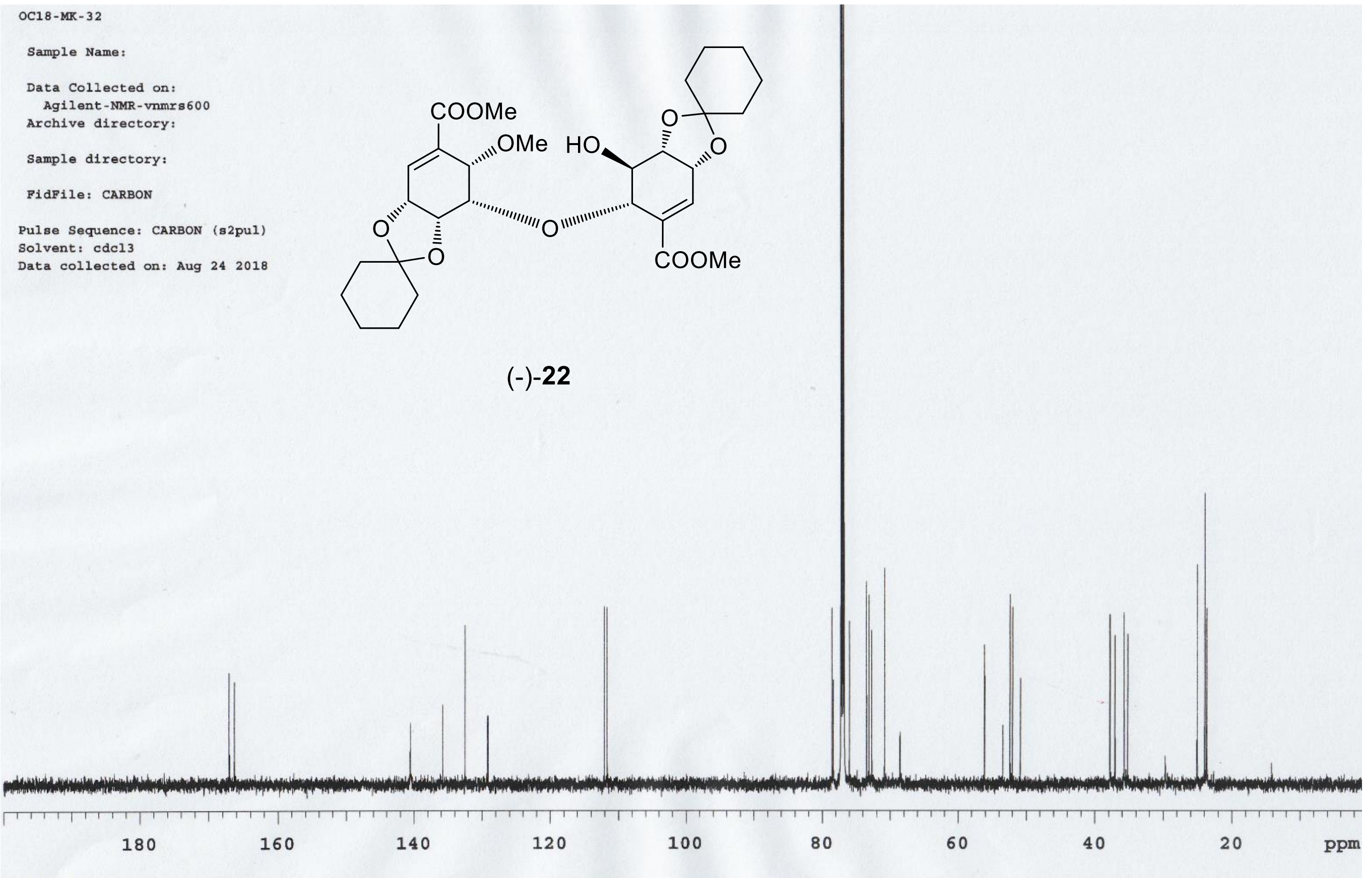

OC18-MK-32

INDEX	FREQUENCY	PPM	HEIGHT
1	25186.0	166.968	21.9
2	25078.5	166.255	20.1
3	21195.6	140.514	12.0
4	20475.5	135.739	15.5
5	19978.4	132.444	31.1
6	19473.2	129.095	13.4
7	16900.0	112.037	34.9
8	16839.9	111.638	34.7
9	11848.5	78.548	34.6
10	11820.7	78.364	20.4
11	11646.2	77.207	963.6
12	11615.0	77.000	962.6
13	11582.6	76.785	1009.2
14	11457.7	75.958	31.9
15	11079.7	73.452	39.7
16	11026.6	73.099	37.0
17	10965.3	72.693	30.2
18	10682.1	70.816	42.2
19	10337.6	68.532	10.2
20	8462.6	56.102	27.3
21	8055.7	53.404	11.6
22	7898.5	52.362	37.2
23	7843.0	51.994	34.7
24	7674.3	50.876	20.8
25	5701.0	37.794	33.2
26	5588.9	37.051	29.1
27	5386.6	35.710	33.6
28	5306.8	35.181	29.4
29	3770.6	24.996	43.0
30	3608.7	23.924	56.8
31	3568.3	23.655	32.2
32	3557.9	23.586	34.4

Figure S17: ^{1}H- NMR Spectrum of (+)-**22** in $CDCl_3$ (400 MHz)

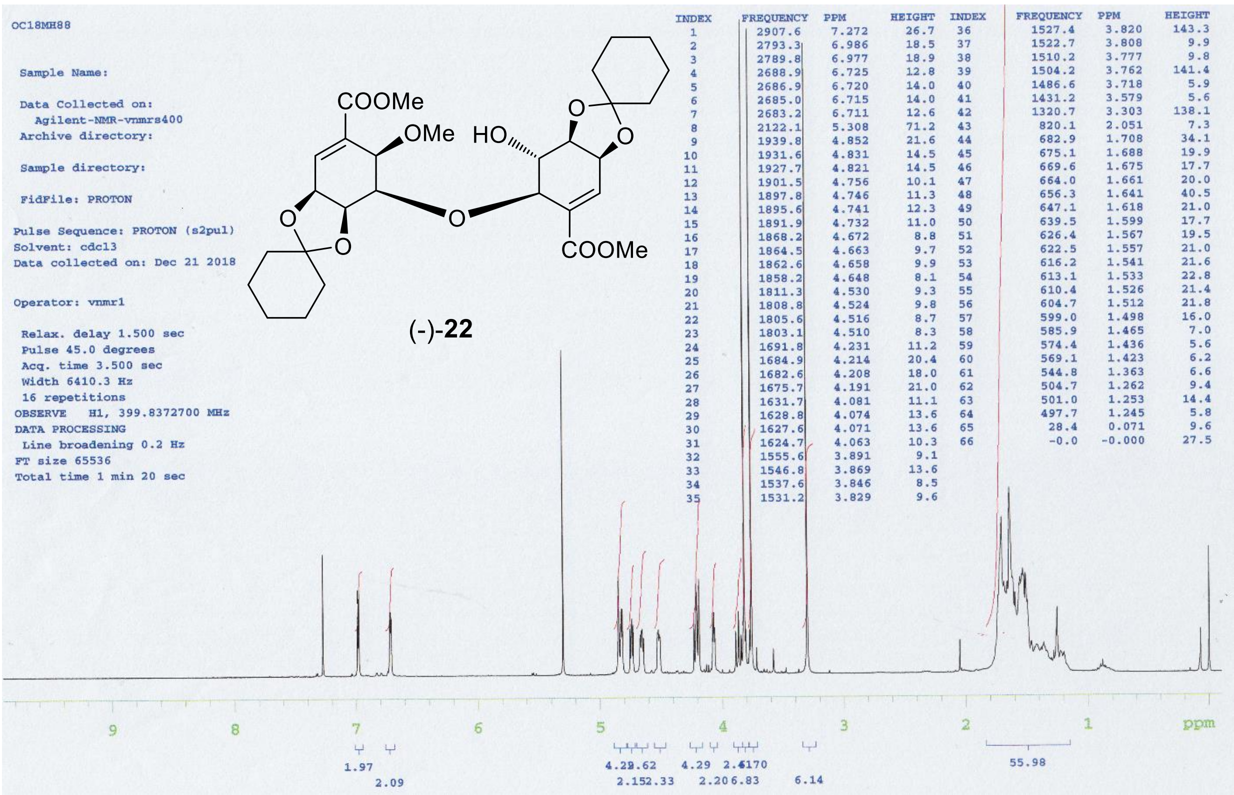

Figure S18: ^{13}C-NMR Spectrum of (+)-**22** in $CDCl_3$ (100 MHz)

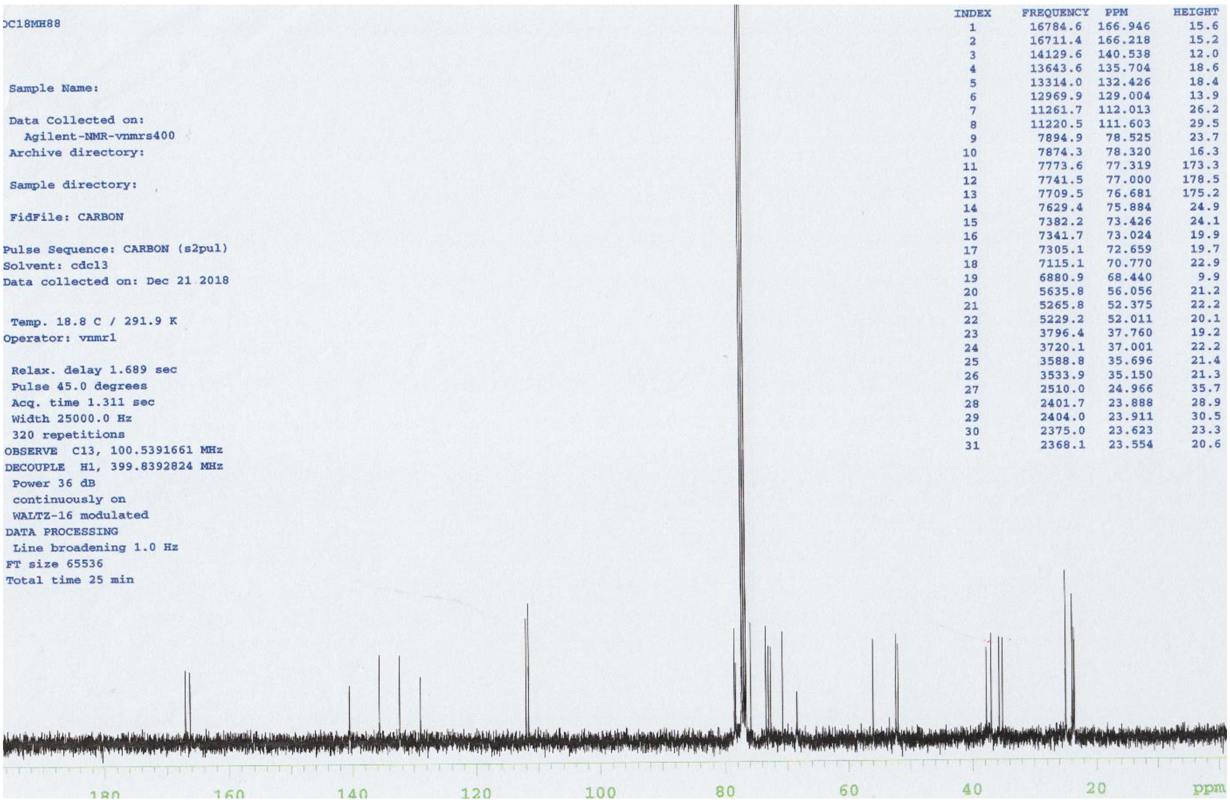

Figure S19: ^{1}H- NMR Spectrum of (+)-**13** in $CDCl_3$ (300 MHz)

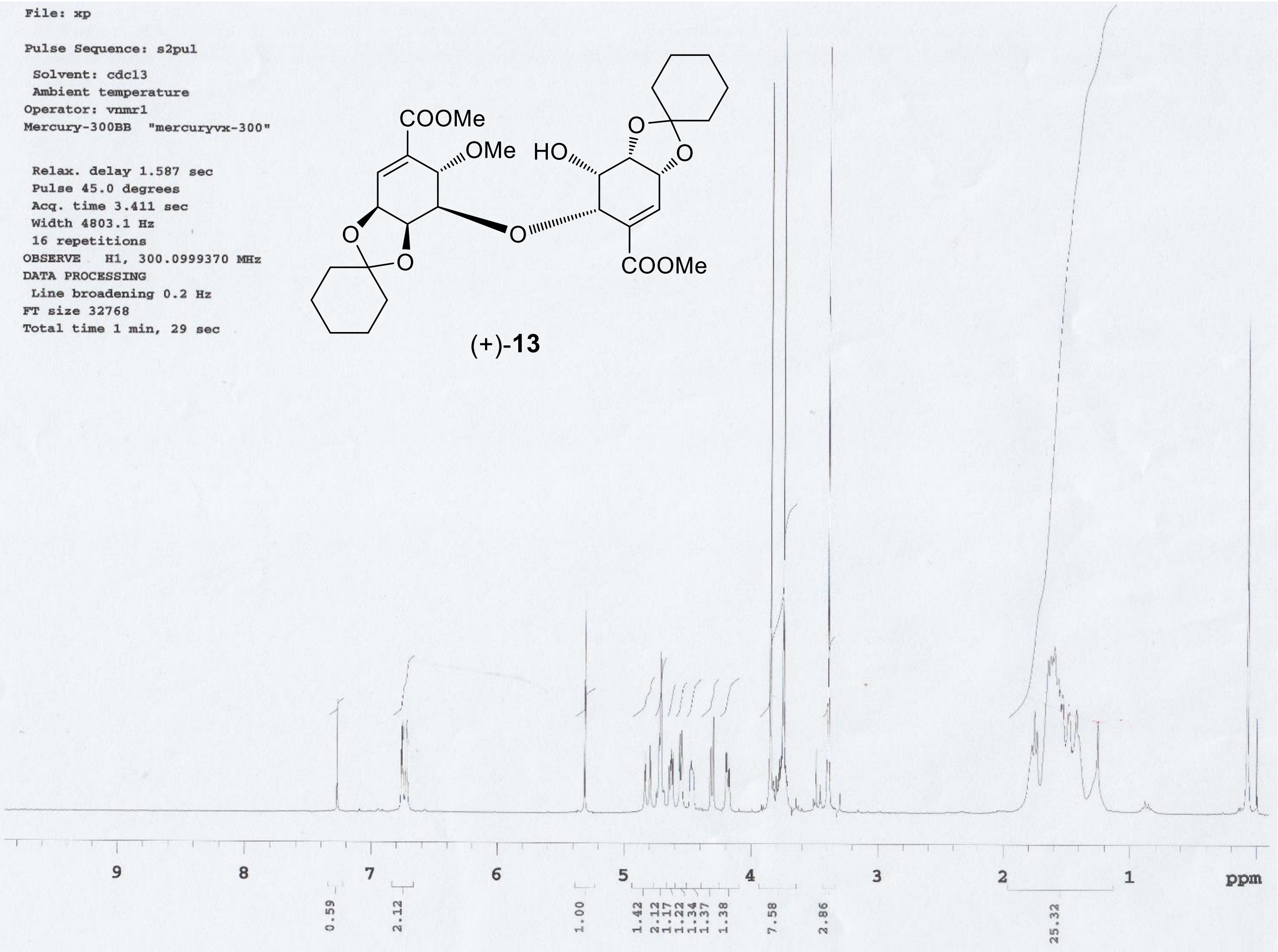

INDEX	FREQUENCY	PPM	HEIGHT	INDEX	FREQUENCY	PPM	HEIGHT
1	2182.3	7.272	23.2	42	1018.1	3.393	153.0
2	2028.9	6.761	17.2	43	531.8	1.772	14.9
3	2028.3	6.759	18.0	44	525.6	1.752	21.4
4	2025.7	6.750	18.9	45	519.8	1.732	17.7
5	2025.1	6.748	17.7	46	494.3	1.647	31.7
6	2016.3	6.719	19.1	47	487.2	1.624	32.3
7	1592.1	5.305	44.7	48	483.1	1.610	32.3
8	1450.5	4.834	13.2	49	479.0	1.596	34.2
9	1438.8	4.794	14.5	50	469.3	1.564	28.0
10	1417.1	4.722	16.8	51	465.8	1.552	25.8
11	1413.6	4.710	32.9	52	459.4	1.531	24.7
12	1393.4	4.643	10.3	53	445.6	1.485	22.8
13	1390.1	4.632	11.7	54	435.3	1.451	13.8
14	1388.1	4.625	13.7	55	426.5	1.421	21.7
15	1384.6	4.614	13.1	56	376.4	1.254	19.4
16	1368.7	4.561	16.7	57	21.4	0.071	100.2
17	1363.8	4.544	17.4	58	0.0	0.000	20.2
18	1344.1	4.479	10.6				
19	1341.2	4.469	11.7				
20	1338.6	4.460	10.2				
21	1335.6	4.451	9.3				
22	1295.5	4.317	14.3				
23	1289.6	4.297	20.2				
24	1260.0	4.199	13.3				
25	1256.8	4.188	13.2				
26	1254.1	4.179	10.9				
27	1250.6	4.167	10.2				
28	1155.3	3.850	145.9				
29	1148.6	3.827	7.8				
30	1147.1	3.822	7.5				
31	1140.7	3.801	8.5				
32	1138.0	3.792	8.1				
33	1133.9	3.779	12.4				
34	1131.0	3.769	10.3				
35	1128.9	3.762	12.4				
36	1124.5	3.747	156.6				
37	1119.6	3.731	10.7				
38	1117.5	3.724	8.8				
39	1114.6	3.714	7.4				
40	1046.9	3.488	13.9				
41	1036.9	3.455	6.7				

Figure S20: ^{13}C-NMR Spectrum of (+)-**13** in $CDCl_3$ (75.5 MHz)

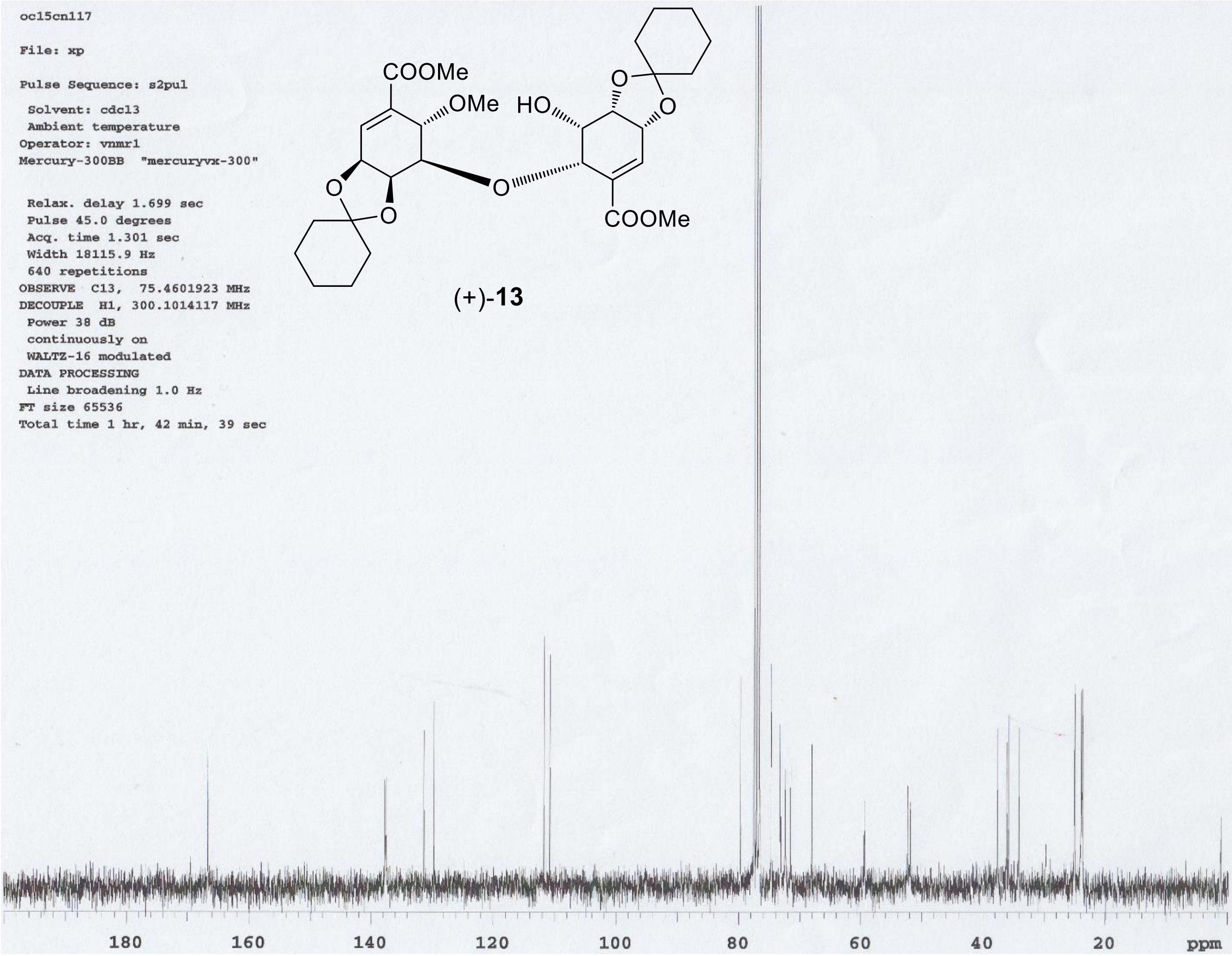

INDEX	FREQUENCY	PPM	HEIGHT
1	12577.4	166.676	29.0
2	12574.1	166.632	30.8
3	10389.2	137.678	22.0
4	10375.9	137.502	22.5
5	9907.6	131.296	32.0
6	9785.5	129.677	37.8
7	8431.0	111.727	51.1
8	8358.5	110.768	47.4
9	6011.1	79.660	43.0
10	5842.5	77.425	212.4
11	5810.4	77.000	224.5
12	5778.4	76.575	217.2
13	5635.7	74.685	35.8
14	5631.9	74.634	45.7
15	5524.1	73.205	33.3
16	5459.9	72.355	24.1
17	5393.6	71.476	24.9
18	5132.6	68.018	29.2
19	4477.5	59.336	17.7
20	3946.2	52.295	20.7
21	3914.7	51.878	17.5
22	2835.0	37.569	32.5
23	2719.4	36.038	29.7
24	2694.5	35.708	35.2
25	2568.5	34.038	32.6
26	1886.3	24.997	41.6
27	1879.6	24.909	39.5
28	1802.2	23.883	39.9
29	1796.2	23.803	40.4
30	1787.9	23.693	40.6
31	1782.9	23.627	35.1
32	75.1	0.996	14.4

Figure S21: ^{1}H- NMR Spectrum of (-)-**13** in $CDCl_3$ (600 MHz)

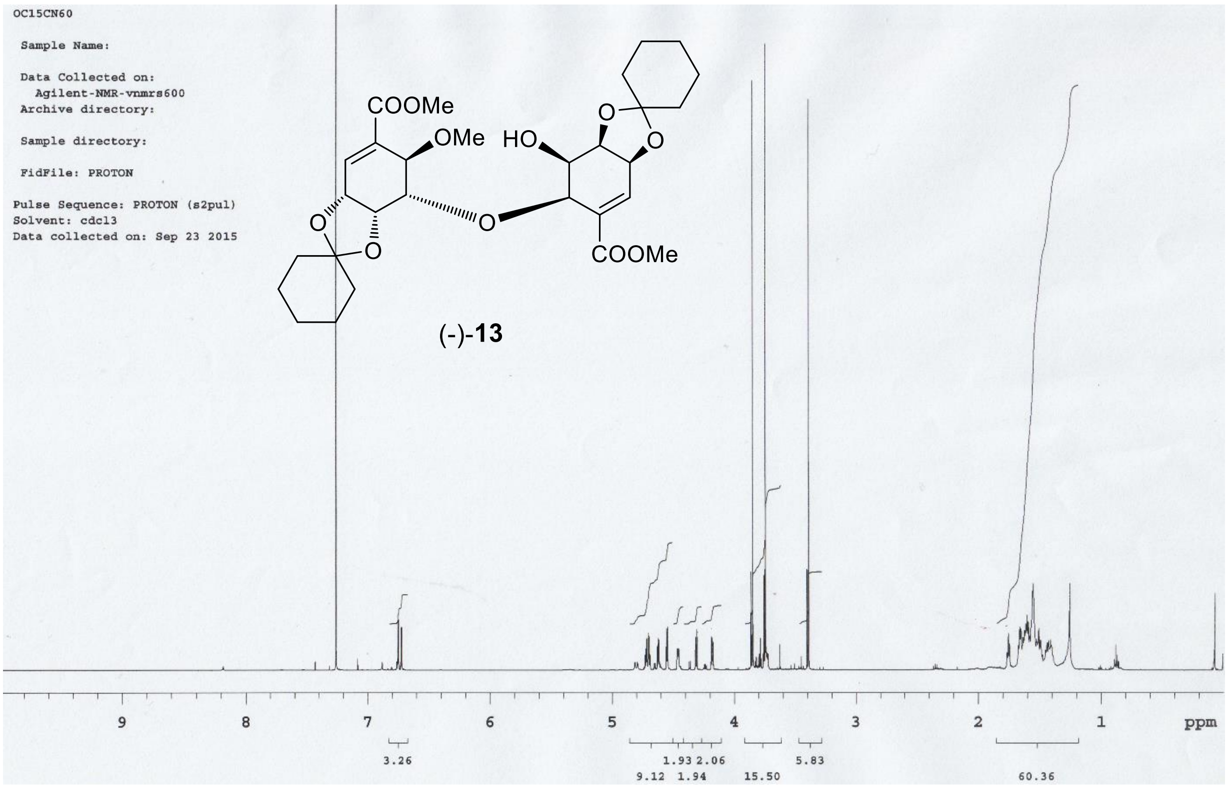

OC15CN60

INDEX	FREQUENCY	PPM	HEIGHT	INDEX	FREQUENCY	PPM	HEIGHT	INDEX	FREQUENCY	PPM	HEIGHT
1	4458.8	7.433	7.1	51	2621.0	4.369	6.7	101	904.4	1.508	34.5
2	4355.8	7.261	1458.3	52	2615.1	4.359	7.5	102	898.2	1.497	22.7
3	4249.9	7.084	9.2	53	2588.7	4.315	27.4	103	895.9	1.493	24.8
4	4129.8	6.884	6.6	54	2582.6	4.305	33.7	104	884.7	1.475	12.5
5	4058.0	6.764	6.7	55	2546.2	4.244	4.9	105	870.6	1.451	16.3
6	4057.1	6.763	6.7	56	2512.4	4.188	27.5	106	864.2	1.441	22.4
7	4054.7	6.759	8.4	57	2508.9	4.182	26.6	107	855.7	1.426	24.4
8	4053.6	6.757	9.1	58	2506.5	4.178	24.2	108	850.4	1.418	18.1
9	4052.7	6.756	38.5	59	2502.7	4.172	23.3	109	845.7	1.410	25.1
10	4051.5	6.754	46.1	60	2317.0	3.862	81.3	110	841.3	1.402	20.4
11	4049.2	6.750	40.9	61	2314.9	3.859	28.3	111	779.1	1.299	8.0
12	4048.3	6.748	41.8	62	2308.5	3.848	488.1	112	752.7	1.255	71.6
13	4039.5	6.734	6.8	63	2297.3	3.830	7.2	113	535.2	0.892	8.7
14	4035.9	6.728	35.3	64	2290.6	3.818	10.6	114	528.2	0.880	20.9
15	4033.6	6.724	35.3	65	2289.4	3.816	7.0	115	520.9	0.868	12.3
16	2886.6	4.812	6.5	66	2276.8	3.795	13.5	116	513.5	0.856	6.7
17	2874.8	4.792	6.8	67	2273.3	3.789	4.9	117	47.2	0.079	7.9
18	2837.5	4.730	12.4	68	2272.1	3.787	13.3	118	46.4	0.077	7.3
19	2833.4	4.723	11.8	69	2269.5	3.783	26.4	119	45.5	0.076	9.1
20	2831.1	4.719	25.8	70	2267.7	3.780	9.5	120	44.9	0.075	13.5
21	2827.6	4.713	26.6	71	2256.2	3.761	12.8	121	41.4	0.069	63.3
22	2821.4	4.703	29.4	72	2253.6	3.757	78.3	122	3.2	0.005	13.2
23	2820.5	4.702	30.7	73	2246.0	3.744	518.6	123	0.0	0.000	566.8
24	2819.1	4.699	30.1	74	2242.2	3.738	19.4	124	-3.2	-0.005	12.1
25	2818.2	4.698	27.7	75	2238.1	3.731	13.8				
26	2815.0	4.692	13.2	76	2236.3	3.728	13.7				
27	2814.1	4.691	13.2	77	2233.4	3.723	13.4				
28	2812.6	4.688	12.4	78	2174.7	3.625	21.2				
29	2811.4	4.687	11.3	79	2103.1	3.506	5.0				
30	2791.8	4.654	5.4	80	2070.8	3.452	11.1				
31	2787.7	4.647	5.7	81	2040.9	3.402	83.3				
32	2777.1	4.629	19.2	82	2033.5	3.390	472.7				
33	2776.2	4.628	20.0	83	1058.7	1.765	14.5				
34	2773.9	4.624	20.0	84	1052.9	1.755	30.2				
35	2773.0	4.622	21.6	85	1046.7	1.745	21.5				
36	2771.5	4.620	26.2	86	1003.9	1.673	20.3				
37	2770.9	4.619	25.5	87	997.7	1.663	35.7				
38	2768.3	4.615	25.0	88	991.5	1.653	34.1				
39	2767.4	4.613	24.7	89	981.3	1.636	27.0				
40	2766.2	4.611	5.8	90	975.1	1.625	32.7				
41	2762.1	4.604	5.1	91	967.8	1.613	38.2				
42	2731.6	4.553	34.3	92	961.6	1.603	45.4				
43	2726.6	4.545	36.0	93	955.4	1.593	39.6				
44	2680.0	4.467	17.6	94	948.1	1.580	28.0				
45	2678.8	4.465	12.9	95	942.5	1.571	37.4				
46	2677.9	4.464	13.2	96	935.2	1.559	65.4				
47	2677.0	4.463	17.9	97	931.4	1.553	71.3				
48	2675.9	4.461	13.4	98	915.8	1.527	23.5				
49	2674.4	4.458	16.4	99	913.5	1.523	25.3				
50	2671.5	4.453	17.7	100	909.7	1.516	22.9				

Figure S22: ^{13}C-NMR Spectrum of (-)-**13** in $CDCl_3$ (150 MHz)

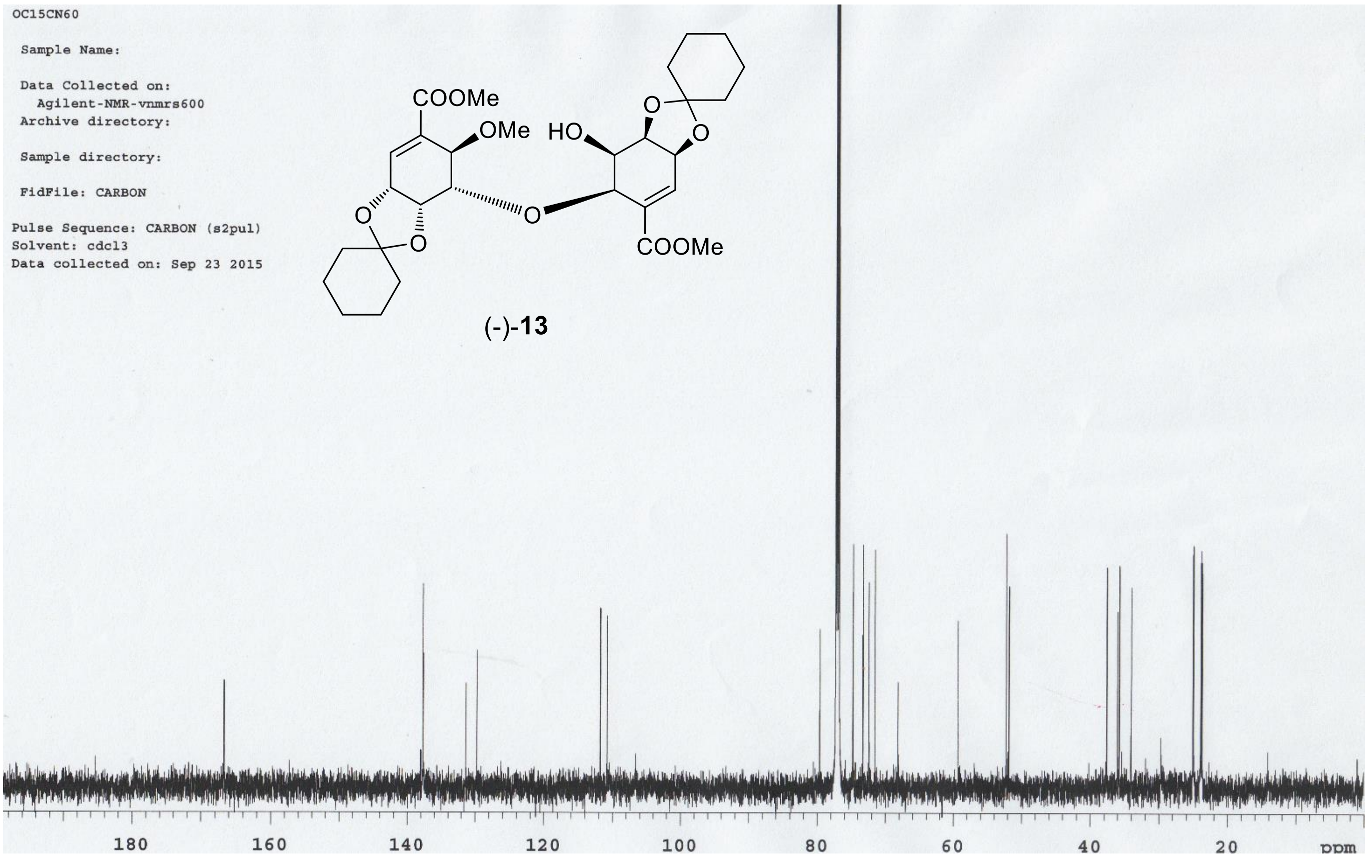

OC15CN60

INDEX	FREQUENCY	PPM	HEIGHT
1	25145.6	166.699	20.6
2	25137.5	166.646	20.7
3	20771.4	137.701	39.5
4	20763.3	137.648	25.9
5	19810.8	131.333	20.0
6	19573.8	129.762	26.5
7	16857.3	111.753	34.8
8	16711.6	110.788	33.2
9	12006.9	79.598	30.8
10	11646.2	77.207	2305.3
11	11615.0	77.000	2467.8
12	11582.6	76.785	2471.3
13	11270.5	74.716	37.9
14	11263.6	74.670	47.3
15	11054.3	73.283	29.6
16	11046.2	73.230	47.2
17	10920.2	72.394	39.9
18	10783.8	71.490	46.3
19	10275.2	68.118	20.4
20	8942.4	59.282	32.5
21	7884.7	52.270	49.4
22	7819.9	51.841	39.1
23	5673.3	37.610	42.9
24	5436.3	36.039	34.3
25	5394.7	35.764	43.2
26	5133.5	34.032	39.0
27	3779.8	25.058	45.3
28	3766.0	24.966	47.1
29	3608.7	23.924	43.8
30	3597.2	23.847	46.2
31	3579.8	23.732	43.8
32	3570.6	23.671	41.7

Figure S23: ^{1}H- NMR Spectrum of (+)-**17** in acetone-d6 (300 MHz)

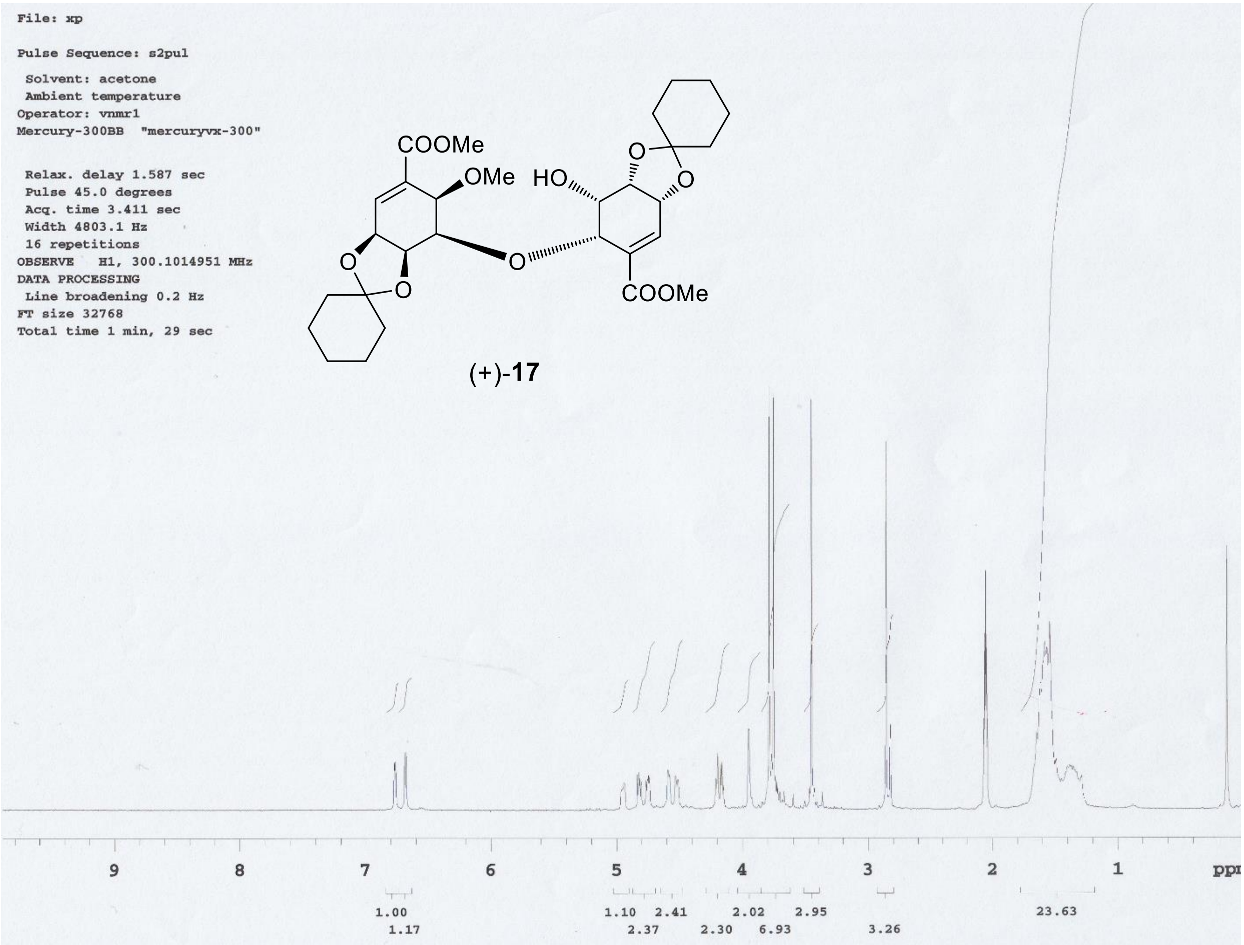

INDEX	FREQUENCY	PPM	HEIGHT	INDEX	FREQUENCY	PPM	HEIGHT
1	2032.2	6.772	18.2	42	616.8	2.055	87.3
2	2029.5	6.763	18.6	43	614.5	2.047	64.0
3	2007.5	6.690	21.7	44	612.4	2.041	33.8
4	2004.0	6.678	22.1	45	494.0	1.646	29.3
5	1482.5	4.940	10.8	46	485.2	1.617	46.7
6	1450.2	4.833	14.0	47	474.9	1.583	61.6
7	1446.7	4.821	14.7	48	469.9	1.566	59.5
8	1444.1	4.812	12.5	49	463.2	1.543	68.9
9	1440.6	4.800	11.4	50	447.9	1.493	19.5
10	1429.1	4.762	11.7	51	435.3	1.451	12.2
11	1426.2	4.752	12.5	52	420.7	1.402	16.7
12	1423.9	4.745	13.9	53	415.4	1.384	17.3
13	1420.9	4.735	12.9	54	411.0	1.370	17.0
14	1377.0	4.588	15.7	55	406.6	1.355	16.8
15	1373.1	4.576	14.1	56	387.8	1.292	13.4
16	1358.8	4.528	13.5	57	39.0	0.130	96.1
17	1353.5	4.510	12.2	58	-0.0	-0.000	60.3
18	1262.0	4.205	12.6				
19	1258.2	4.193	21.5				
20	1250.9	4.168	17.8				
21	1248.0	4.158	18.4				
22	1243.9	4.145	10.0				
23	1184.4	3.947	30.5				
24	1176.7	3.921	5.6				
25	1136.0	3.785	153.9				
26	1130.4	3.767	21.2				
27	1125.7	3.751	151.5				
28	1121.0	3.736	12.8				
29	1117.5	3.724	11.0				
30	1115.2	3.716	9.2				
31	1110.5	3.700	7.7				
32	1100.8	3.668	7.7				
33	1079.4	3.597	6.7				
34	1040.4	3.467	8.7				
35	1035.1	3.449	149.2				
36	1028.7	3.428	8.9				
37	1009.9	3.365	7.5				
38	856.9	2.855	133.8				
39	846.6	2.821	31.9				
40	620.9	2.069	34.0				
41	618.9	2.062	64.5				

Figure S24: ^{13}C-NMR Spectrum of (+)-**17** in acetone-d_6 (75.5 MHz)

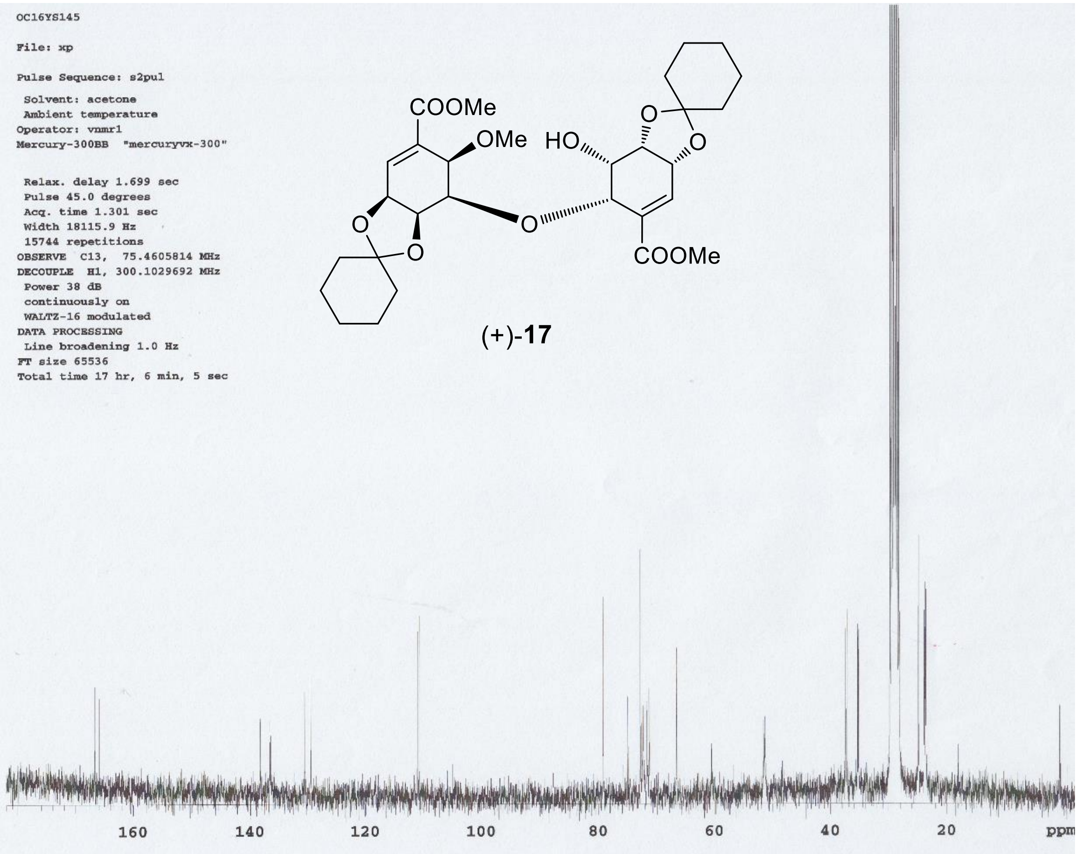

INDEX	FREQUENCY	PPM	HEIGHT	INDEX	FREQUENCY	PPM	HEIGHT
1	16267.6	215.578	7.9	42	4252.9	56.360	-8.4
2	15511.2	205.555	70.0	43	3888.6	51.532	14.4
3	15491.3	205.292	4691.5	44	3872.0	51.312	15.2
4	15470.9	205.021	73.5	45	3687.9	48.872	-6.8
5	12571.7	166.601	22.0	46	3341.3	44.278	-8.6
6	12520.3	165.919	19.4	47	3101.3	41.099	-7.8
7	12105.1	160.417	-6.9	48	2828.2	37.479	35.3
8	11540.6	152.937	-7.6	49	2809.4	37.230	39.7
9	11333.9	150.197	-7.9	50	2667.9	35.355	36.4
10	10564.3	139.998	-8.5	51	2654.1	35.172	35.3
11	10431.6	138.240	15.1	52	2241.6	29.706	585.4
12	10323.3	136.804	-7.5	53	2222.3	29.450	1765.7
13	10301.1	136.511	14.4	54	2202.9	29.193	3518.9
14	10004.3	132.577	-8.2	55	2183.6	28.937	4113.7
15	9850.6	130.540	21.0	56	2164.2	28.680	3523.5
16	9820.7	130.144	-7.5	57	2144.9	28.424	1766.1
17	9767.6	129.441	25.0	58	2125.5	28.168	586.8
18	9317.6	123.477	-9.0	59	1872.9	24.819	57.0
19	8793.0	116.524	-8.1	60	1799.9	23.852	39.5
20	8377.8	111.022	35.2	61	1789.4	23.713	46.1
21	8358.4	110.766	38.6	62	1783.3	23.633	48.8
22	8090.8	107.220	-8.1	63	1776.1	23.537	44.7
23	7967.5	105.586	-7.8	64	1393.0	18.460	-8.1
24	7324.6	97.065	-8.7	65	1357.1	17.984	8.7
25	6685.5	88.596	-9.4	66	1176.3	15.588	-7.3
26	6284.1	83.277	-8.9	67	839.0	11.119	-8.5
27	5975.6	79.189	42.9	68	267.9	3.551	-8.5
28	5800.4	76.866	-8.0	69	38.0	0.503	17.6
29	5652.7	74.910	19.9	70	-48.3	-0.640	-8.9
30	5588.6	74.060	-6.7				
31	5490.2	72.756	53.6				
32	5468.1	72.463	17.8				
33	5453.2	72.265	17.9				
34	5402.9	71.599	18.7				
35	5375.8	71.240	21.8				
36	5272.9	69.877	-7.6				
37	5019.2	66.514	31.0				
38	4883.7	64.719	-8.3				
39	4667.0	61.847	-8.4				
40	4564.2	60.485	8.8				
41	4344.1	57.569	-7.6				

Figure S25: ^{1}H- NMR Spectrum of (-)-**17** in acetone-d$_6$ (600 MHz)

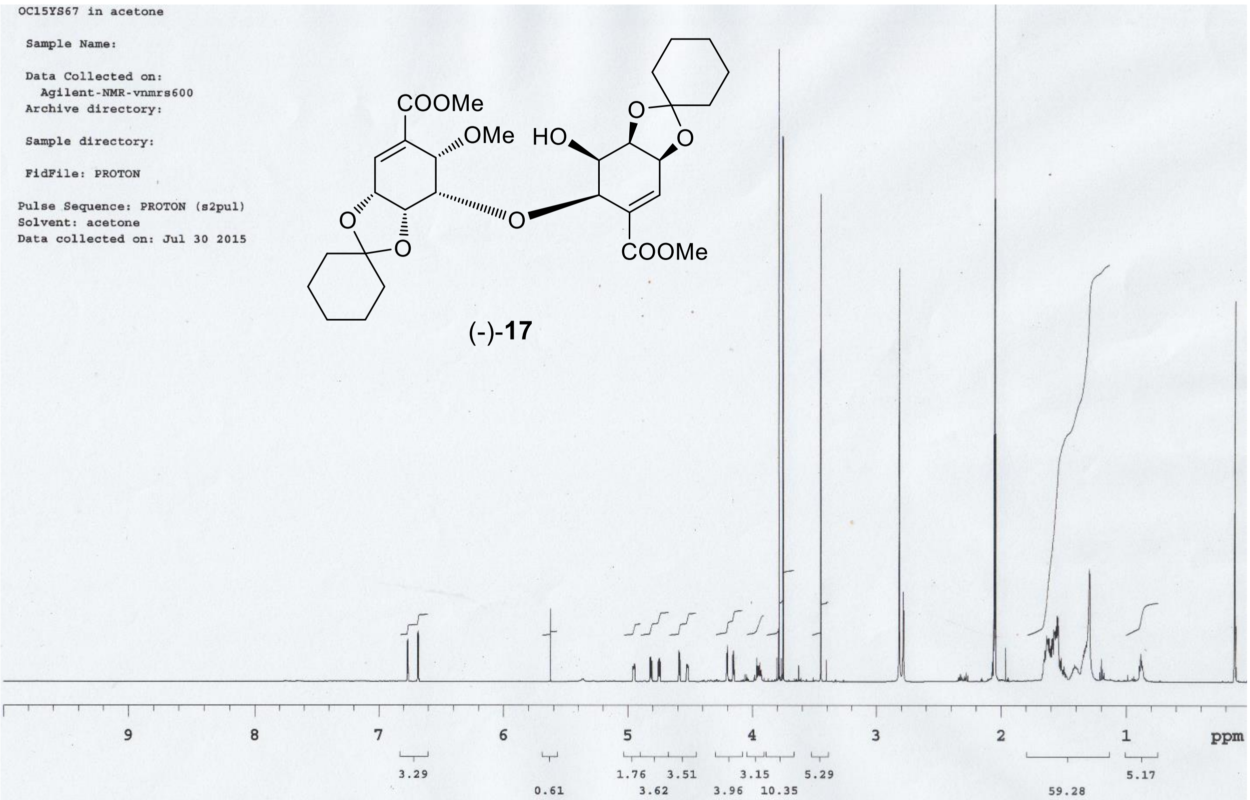

OC15YS67 in acetone

INDEX	FREQUENCY	PPM	HEIGHT	INDEX	FREQUENCY	PPM	HEIGHT	INDEX	FREQUENCY	PPM	HEIGHT
1	4061.8	6.771	36.8	51	2364.5	3.942	17.3	101	897.6	1.496	14.1
2	4060.6	6.769	38.1	52	2361.0	3.936	10.1	102	894.4	1.491	9.0
3	4058.5	6.765	36.3	53	2358.4	3.931	10.2	103	889.4	1.483	7.6
4	4057.7	6.764	34.8	54	2355.7	3.927	10.8	104	860.9	1.435	8.1
5	4011.9	6.688	43.7	55	2280.0	3.801	21.8	105	858.0	1.430	9.3
6	4011.0	6.686	44.6	56	2270.6	3.785	566.7	106	854.2	1.424	12.6
7	4008.4	6.682	45.6	57	2258.9	3.765	21.4	107	851.0	1.419	12.9
8	4007.5	6.680	42.2	58	2250.4	3.751	488.9	108	847.7	1.413	15.5
9	3373.7	5.624	65.9	59	2176.1	3.628	14.8	109	844.8	1.408	13.9
10	2974.6	4.958	13.8	60	2069.0	3.449	437.3	110	841.3	1.402	14.1
11	2973.1	4.956	12.6	61	2042.9	3.405	19.9	111	834.5	1.391	12.3
12	2971.6	4.954	14.3	62	1690.2	2.817	370.9	112	827.5	1.379	8.9
13	2969.6	4.950	12.4	63	1670.0	2.784	80.7	113	822.2	1.371	7.7
14	2968.4	4.948	16.3	64	1395.0	2.325	7.6	114	815.8	1.360	13.1
15	2967.0	4.946	14.5	65	1366.8	2.278	9.0	115	809.0	1.349	19.8
16	2965.5	4.943	16.2	66	1243.9	2.073	16.6	116	806.4	1.344	24.4
17	2893.0	4.822	21.7	67	1241.5	2.070	18.5	117	801.7	1.336	28.3
18	2892.4	4.822	22.1	68	1239.5	2.066	17.2	118	797.9	1.330	30.9
19	2889.5	4.817	22.4	69	1236.0	2.060	221.5	119	793.2	1.322	33.4
20	2888.9	4.816	22.6	70	1233.9	2.057	433.0	120	776.1	1.294	101.4
21	2886.8	4.812	19.5	71	1231.6	2.053	657.5	121	760.3	1.267	8.0
22	2886.3	4.811	19.5	72	1229.5	2.050	433.2	122	727.1	1.212	8.9
23	2882.7	4.805	19.0	73	1227.2	2.046	222.6	123	719.8	1.200	20.8
24	2852.5	4.755	18.0	74	1223.9	2.040	7.9	124	712.8	1.188	11.6
25	2851.6	4.754	19.3	75	1179.6	1.966	30.9	125	707.2	1.179	8.7
26	2849.6	4.750	19.6	76	1001.2	1.669	7.8	126	537.3	0.896	14.8
27	2848.7	4.749	20.0	77	995.6	1.660	20.7	127	535.2	0.892	12.7
28	2847.2	4.746	21.5	78	994.5	1.658	19.2	128	531.7	0.886	21.1
29	2846.4	4.745	20.8	79	989.8	1.650	28.2	129	530.5	0.884	25.6
30	2844.3	4.741	19.7	80	985.7	1.643	28.7	130	529.1	0.882	21.2
31	2843.4	4.740	18.8	81	982.1	1.637	41.6	131	528.2	0.880	24.0
32	2753.6	4.590	28.4	82	978.0	1.630	37.9	132	527.0	0.879	15.5
33	2748.6	4.582	26.3	83	973.6	1.623	39.2	133	525.0	0.875	19.6
34	2715.8	4.527	16.0	84	969.5	1.616	39.3	134	522.9	0.872	14.2
35	2713.4	4.523	16.1	85	965.4	1.609	30.1	135	520.9	0.868	14.9
36	2710.5	4.518	15.4	86	960.7	1.601	32.6	136	518.5	0.864	10.4
37	2708.1	4.514	14.7	87	956.6	1.595	38.8	137	77.5	0.129	341.5
38	2524.2	4.208	19.2	88	952.8	1.588	39.3	138	75.7	0.126	60.6
39	2523.0	4.206	25.8	89	947.2	1.579	47.3	139	74.8	0.125	59.0
40	2522.1	4.204	21.3	90	942.5	1.571	44.9	140	73.7	0.123	50.2
41	2519.8	4.200	25.9	91	939.0	1.565	46.5	141	69.8	0.116	14.0
42	2518.9	4.199	33.1	92	935.8	1.560	49.9	142	3.5	0.006	8.0
43	2494.8	4.159	24.5	93	931.4	1.553	59.9	143	0.0	0.000	385.6
44	2491.9	4.154	26.4	94	927.0	1.545	57.8	144	-3.2	-0.005	11.9
45	2490.7	4.152	27.9	95	924.0	1.540	34.9				
46	2487.8	4.147	18.9	96	919.0	1.532	20.6				
47	2377.1	3.963	21.6	97	914.9	1.525	19.0				
48	2371.9	3.954	14.2	98	910.5	1.518	21.5				
49	2369.5	3.950	13.9	99	906.4	1.511	11.1				
50	2367.2	3.946	14.5	100	901.4	1.503	11.5				

Figure S26: ^{13}C-NMR Spectrum of (-)-**17** in acetone-d$_6$ (150 MHz)

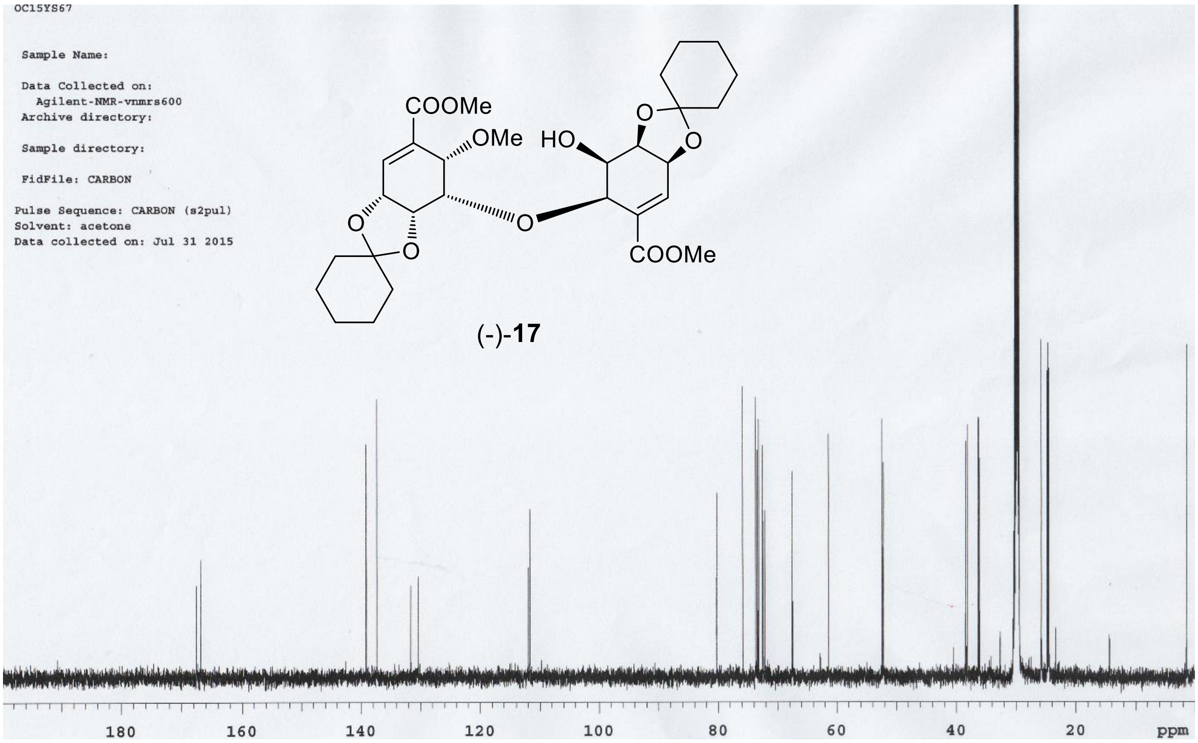

OC15YS67

INDEX	FREQUENCY	PPM	HEIGHT
1	31112.9	206.258	68.6
2	31093.3	206.128	5439.8
3	31072.4	205.990	77.0
4	25268.3	167.512	20.9
5	25163.1	166.815	26.8
6	20987.8	139.135	52.6
7	20715.0	137.327	62.7
8	19849.1	131.587	21.0
9	19668.8	130.391	23.1
10	16884.1	111.930	25.0
11	16844.8	111.670	38.1
12	12101.8	80.227	41.7
13	11434.8	75.805	65.5
14	11110.0	73.652	63.1
15	11069.6	73.384	51.3
16	11037.2	73.169	58.2
17	10935.5	72.495	52.3
18	10884.6	72.158	20.2
19	10881.1	72.135	37.8
20	10187.6	67.537	46.5
21	10171.4	67.429	17.4
22	9261.6	61.398	54.9
23	7910.3	52.440	58.3
24	7875.6	52.210	48.6
25	5796.0	38.424	53.1
26	5756.7	38.163	56.9
27	5471.2	36.270	58.5
28	5444.6	36.094	49.4
29	4924.4	32.646	10.6
30	4584.6	30.393	33.0
31	4577.6	30.347	32.7
32	4559.1	30.224	565.7
33	4540.6	30.101	1492.4
34	4521.0	29.971	3756.1
35	4501.3	29.841	4543.1
36	4481.7	29.711	3261.1
37	4463.2	29.588	1537.3
38	4443.5	29.458	638.3
39	3888.7	25.779	75.9
40	3884.1	25.749	66.0
41	3737.2	24.775	68.9
42	3717.6	24.645	75.2
43	3708.3	24.584	53.2
44	3693.3	24.484	69.6
45	3519.9	23.335	11.4
46	2165.1	14.353	8.9
47	2167.4	14.369	9.9
48	212.7	1.410	74.8
49	-0.0	-0.000	21.0

Figure S27: ^{1}H- NMR Spectrum of (-)-**20** in acetone-d_6 (600 MHz)

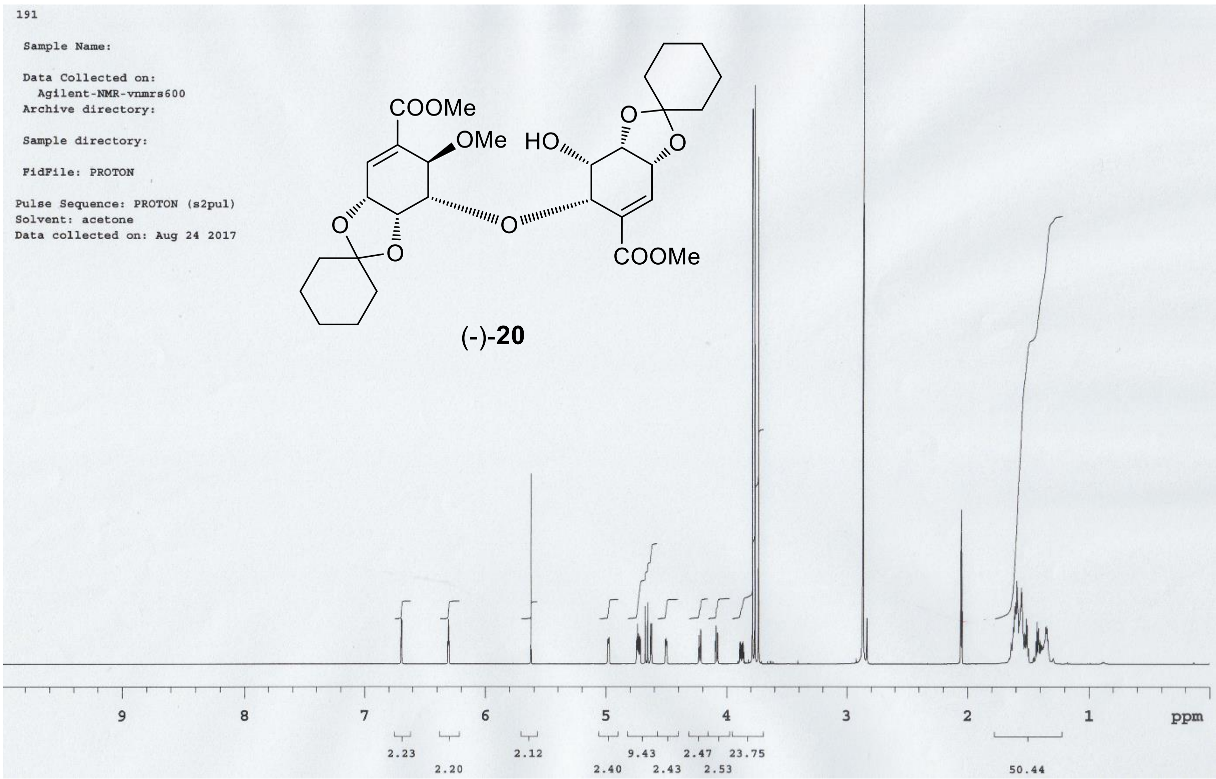

INDEX	FREQUENCY	PPM	HEIGHT	INDEX	FREQUENCY	PPM	HEIGHT	INDEX	FREQUENCY	PPM	HEIGHT
1	4018.3	6.698	45.6	51	2527.1	4.213	19.6	101	830.1	1.384	17.2
2	4017.5	6.697	48.5	52	2453.7	4.090	41.6	102	820.2	1.367	22.2
3	4015.4	6.693	47.9	53	2451.4	4.086	40.4	103	814.3	1.357	34.3
4	4014.2	6.692	46.4	54	2445.2	4.076	33.5	104	808.7	1.348	35.3
5	3786.5	6.312	25.7	55	2442.9	4.072	33.2	105	802.8	1.338	22.4
6	3785.1	6.309	38.6	56	2334.0	3.891	25.9	106	-0.0	-0.000	74.3
7	3783.6	6.307	49.1	57	2331.1	3.886	26.8				
8	3782.1	6.305	39.3	58	2329.0	3.882	24.8				
9	3780.4	6.302	23.9	59	2326.1	3.877	23.2				
10	3374.0	5.624	238.1	60	2321.7	3.870	24.9				
11	2991.0	4.986	19.3	61	2318.7	3.865	26.4				
12	2989.5	4.983	24.6	62	2316.7	3.862	24.5				
13	2987.5	4.980	21.2	63	2313.8	3.857	22.9				
14	2986.3	4.978	22.4	64	2269.5	3.783	754.1				
15	2984.3	4.975	25.7	65	2258.3	3.764	812.1				
16	2982.8	4.972	20.4	66	2255.1	3.759	8.1				
17	2847.5	4.747	27.2	67	2240.1	3.734	635.4				
18	2845.8	4.744	28.1	68	1719.5	2.866	2581.2				
19	2844.6	4.742	29.3	69	1699.6	2.833	44.4				
20	2842.5	4.738	45.5	70	1237.4	2.063	64.9				
21	2840.8	4.735	26.8	71	1235.1	2.059	151.6				
22	2839.6	4.733	27.2	72	1233.0	2.055	191.0				
23	2837.0	4.729	29.3	73	1230.7	2.051	150.6				
24	2836.1	4.728	28.2	74	1228.6	2.048	63.1				
25	2833.7	4.724	25.1	75	985.7	1.643	20.5				
26	2832.9	4.722	28.5	76	981.8	1.637	12.1				
27	2831.4	4.720	27.9	77	977.4	1.629	23.5				
28	2830.5	4.718	30.1	78	974.2	1.624	34.0				
29	2828.5	4.715	27.7	79	968.6	1.615	53.7				
30	2827.3	4.713	27.4	80	962.5	1.604	56.4				
31	2803.8	4.674	65.2	81	957.5	1.596	77.3				
32	2791.2	4.653	70.1	82	954.6	1.591	57.6				
33	2777.4	4.630	41.0	83	951.3	1.586	48.9				
34	2772.4	4.621	41.4	84	946.3	1.577	36.8				
35	2705.2	4.509	15.3	85	941.3	1.569	46.8				
36	2704.3	4.508	26.7	86	938.4	1.564	53.8				
37	2703.2	4.506	18.8	87	934.6	1.558	70.5				
38	2702.3	4.505	18.6	88	930.8	1.552	57.1				
39	2701.4	4.503	27.4	89	927.3	1.546	29.1				
40	2700.2	4.501	21.3	90	923.7	1.540	27.5				
41	2698.8	4.499	25.8	91	916.7	1.528	29.7				
42	2697.6	4.497	16.5	92	910.8	1.518	44.1				
43	2697.0	4.496	16.9	93	905.0	1.509	38.2				
44	2695.8	4.494	24.9	94	899.1	1.499	15.5				
45	2694.9	4.492	13.4	95	869.8	1.450	7.8				
46	2539.1	4.233	16.0	96	862.7	1.438	22.3				
47	2537.4	4.230	30.1	97	856.5	1.428	40.8				
48	2535.6	4.227	16.7	98	849.5	1.416	34.1				
49	2530.6	4.218	20.2	99	842.8	1.405	23.4				
50	2528.9	4.215	36.5	100	836.3	1.394	18.1				

Figure S28: ^{13}C-NMR Spectrum of (-)-**20** in acetone-d_6 (150 MHz)

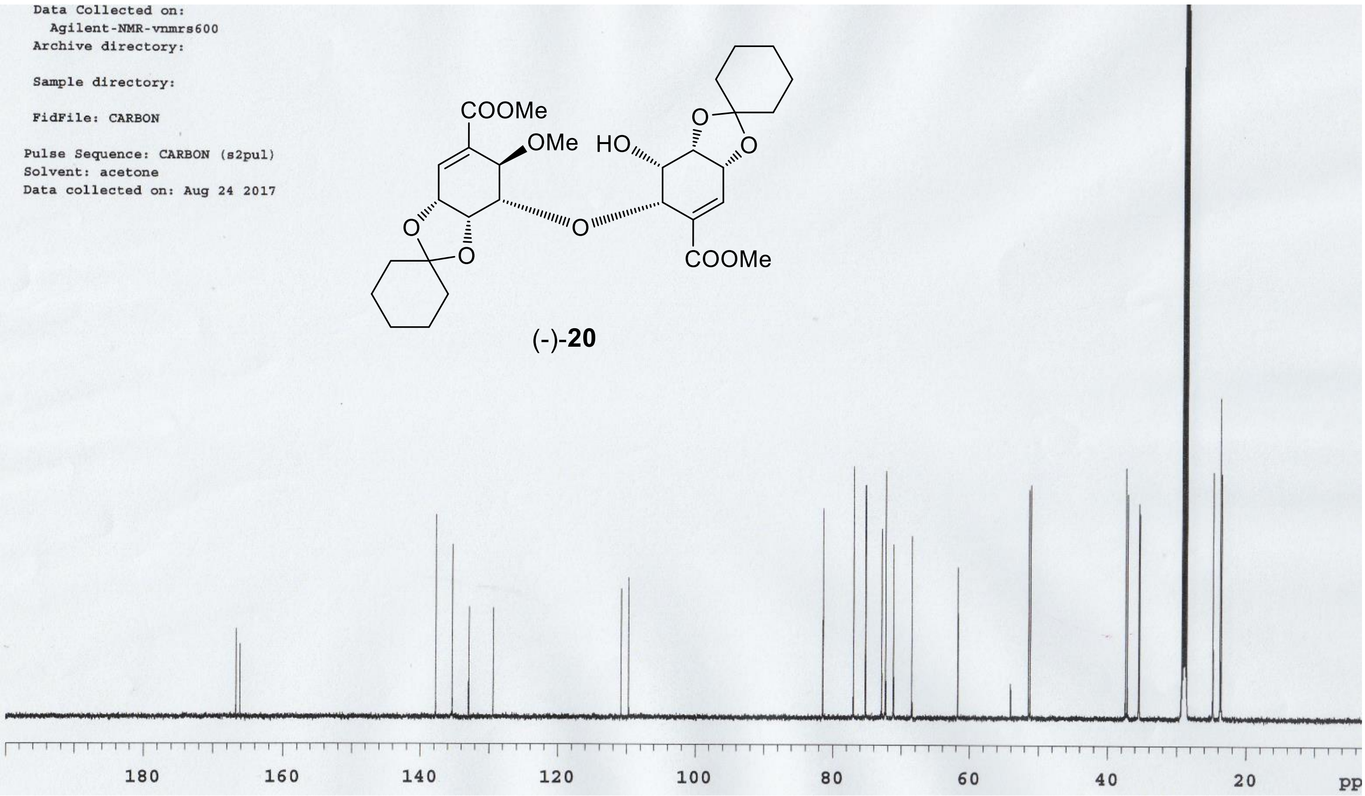

INDEX	FREQUENCY	PPM	HEIGHT
1	25143.6	166.685	17.1
2	25052.3	166.080	14.2
3	20779.8	137.756	39.2
4	20415.7	135.342	33.5
5	20046.9	132.898	21.5
6	19518.6	129.396	21.2
7	16718.9	110.835	25.0
8	16552.4	109.732	27.2
9	12283.4	81.431	40.6
10	11611.8	76.979	48.8
11	11345.9	75.216	45.2
12	10992.2	72.871	36.8
13	10911.3	72.335	47.9
14	10906.7	72.304	42.5
15	10734.4	71.162	33.7
16	10334.5	68.511	35.4
17	9314.9	61.752	29.4
18	8155.5	54.065	6.9
19	7763.6	51.468	44.4
20	7726.6	51.222	45.3
21	5649.3	37.451	48.6
22	5607.7	37.175	43.6
23	5359.2	35.528	41.8
24	5346.5	35.444	39.8
25	4422.8	29.321	126.2
26	4404.4	29.198	313.8
27	4384.7	29.068	808.9
28	4365.1	28.937	1004.1
29	4345.4	28.807	741.2
30	4326.9	28.685	323.3
31	4307.3	28.554	137.2
32	3742.0	24.807	47.8
33	3737.4	24.776	45.3
34	3584.8	23.765	62.3
35	3561.7	23.611	47.5
36	3559.3	23.596	43.5

Figure S29: ^{1}H- NMR Spectrum of (+)-**20** in acetone-d$_6$ (400 MHz)

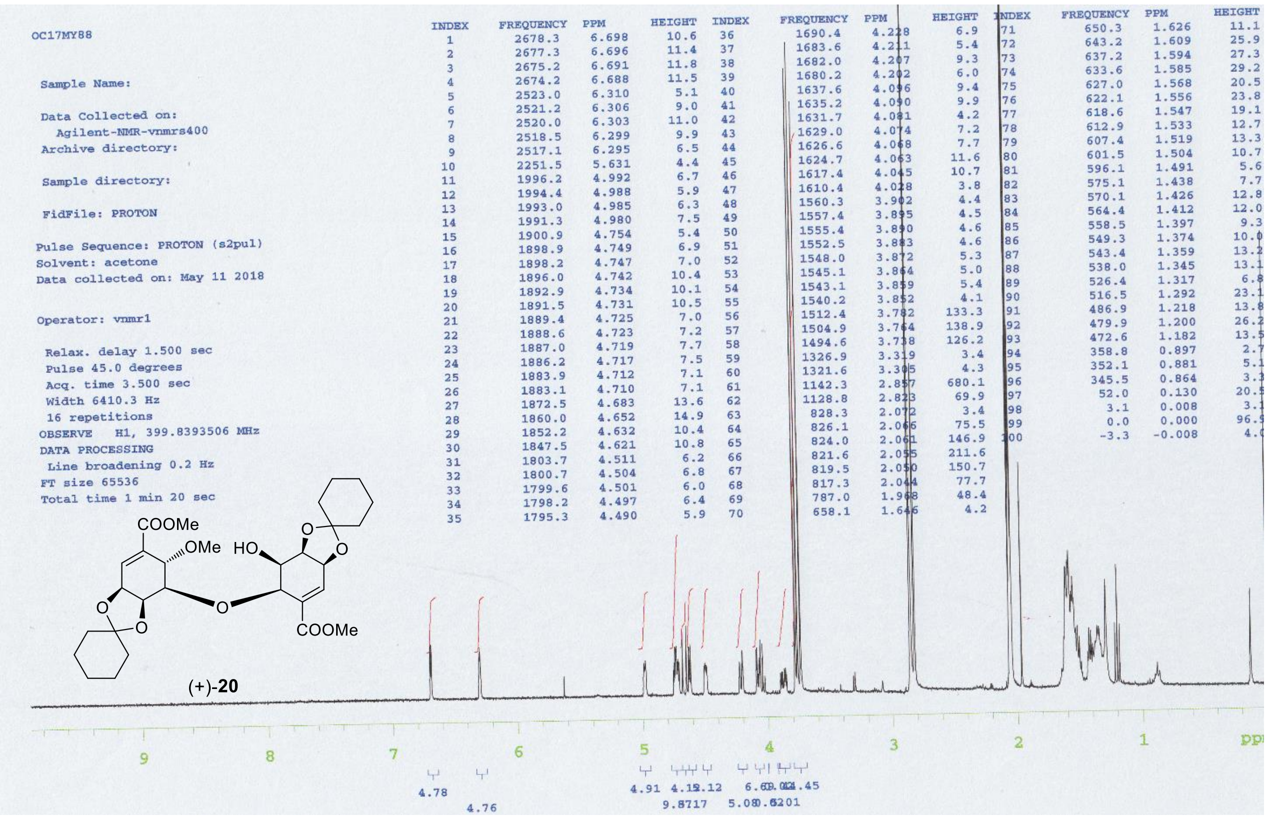

Figure S29: ^{13}C-NMR Spectrum of (+)-**20** in acetone-d$_6$ (100 MHz)

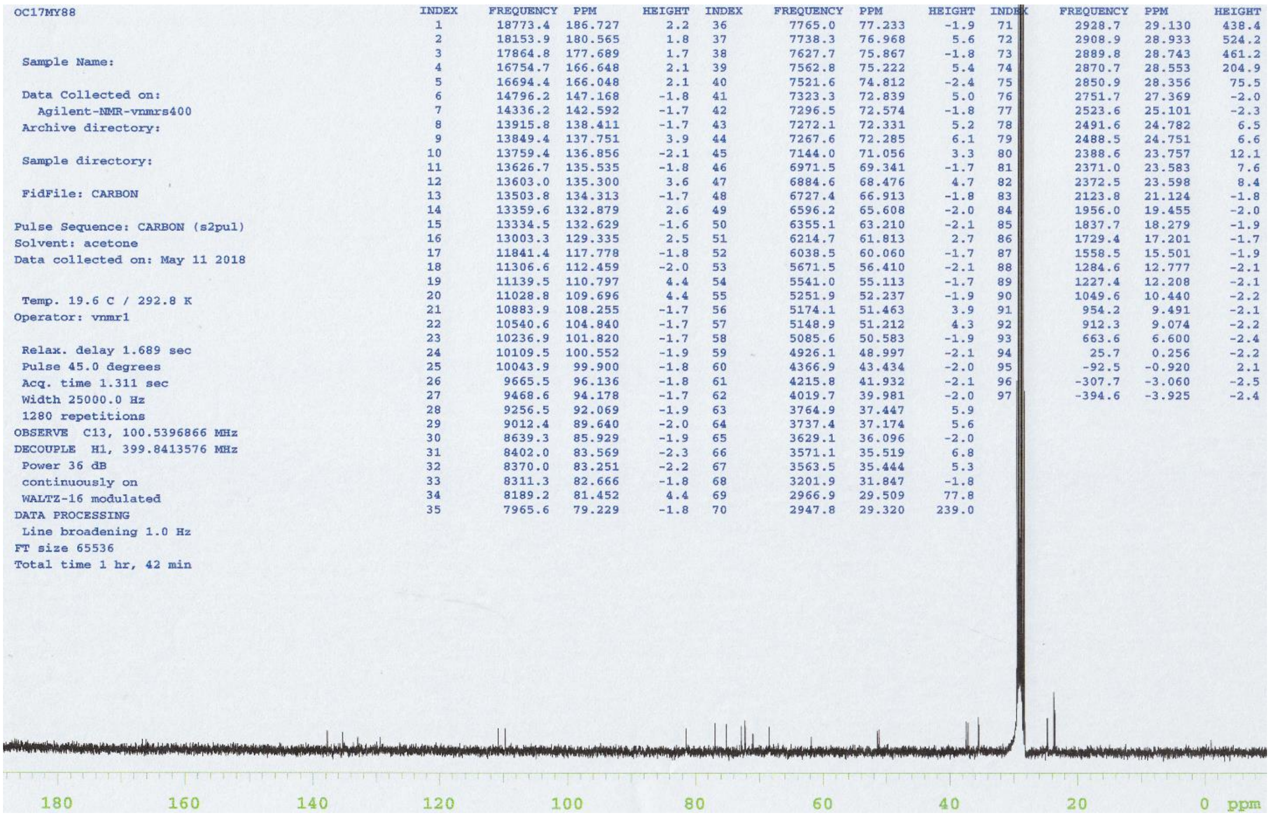

Figure S31: ^{1}H- NMR Spectrum of (+)-**23** in $CDCl_3$ (600 MHz)

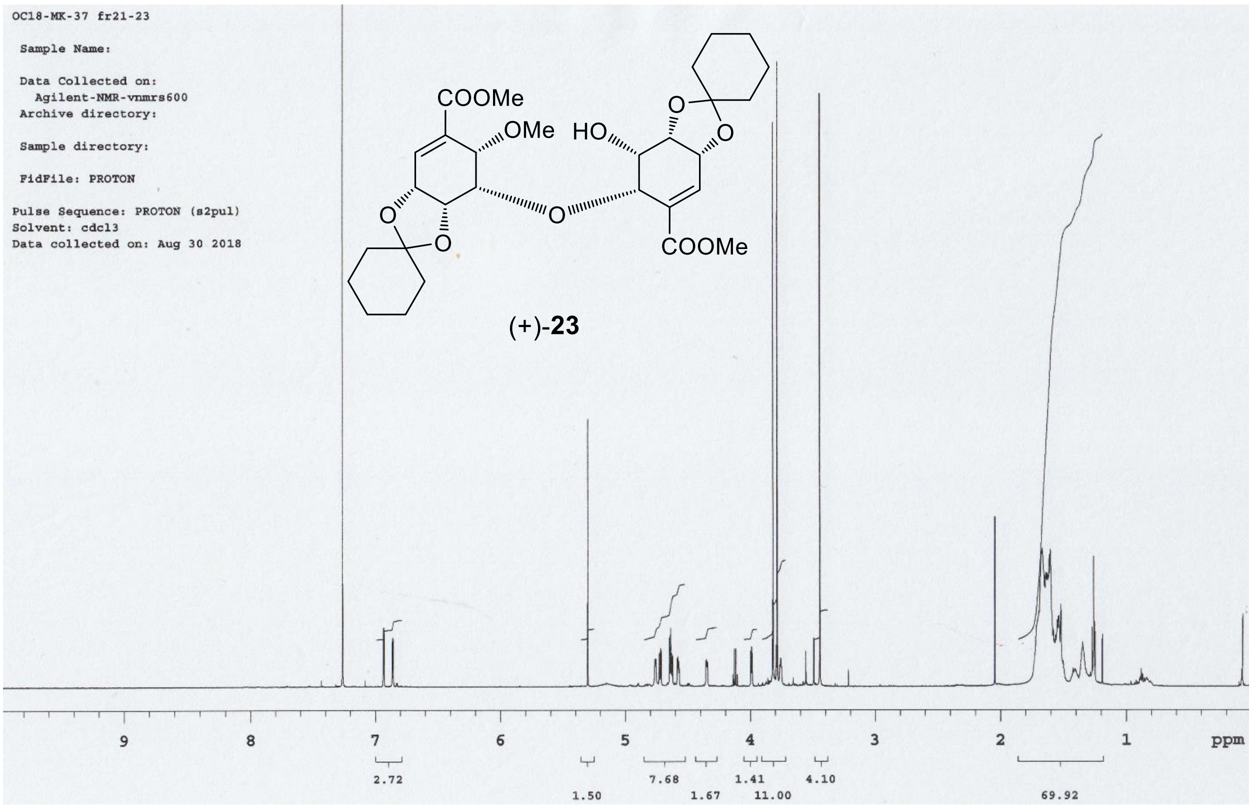

OC18-MK-37 fr21-23

INDEX	FREQUENCY	PPM	HEIGHT	INDEX	FREQUENCY	PPM	HEIGHT
1	4356.7	7.262	748.4	51	1227.2	2.046	101.8
2	4162.7	6.939	32.1	52	1025.0	1.709	39.5
3	4161.8	6.938	35.2	53	1017.4	1.696	45.1
4	4158.9	6.933	35.7	54	1005.0	1.675	80.8
5	4158.0	6.931	32.6	55	1001.5	1.669	83.1
6	4118.4	6.865	27.2	56	988.9	1.648	63.5
7	4117.8	6.864	29.2	57	984.5	1.641	68.2
8	4115.2	6.860	29.3	58	980.7	1.635	67.9
9	4114.3	6.858	28.2	59	976.9	1.628	68.2
10	3179.7	5.300	160.6	60	971.0	1.619	66.9
11	2857.2	4.763	16.7	61	967.5	1.613	78.9
12	2852.8	4.756	16.8	62	962.5	1.604	82.1
13	2835.8	4.727	19.7	63	948.4	1.581	34.3
14	2832.3	4.721	20.6	64	941.1	1.569	28.1
15	2830.2	4.718	23.1	65	936.7	1.561	31.3
16	2826.4	4.711	21.9	66	930.2	1.551	40.0
17	2786.5	4.645	29.5	67	923.7	1.540	42.0
18	2781.2	4.636	35.2	68	921.1	1.535	42.7
19	2776.8	4.629	19.3	69	917.9	1.530	36.2
20	2775.0	4.626	20.0	70	911.7	1.520	49.4
21	2771.5	4.620	18.5	71	900.6	1.501	15.2
22	2749.8	4.584	17.1	72	853.0	1.422	11.2
23	2746.6	4.578	18.0	73	845.1	1.409	10.5
24	2743.6	4.574	15.5	74	841.3	1.402	10.6
25	2740.7	4.569	14.8	75	838.4	1.397	9.4
26	2612.5	4.355	15.7	76	808.4	1.348	26.6
27	2609.5	4.350	16.7	77	791.7	1.320	10.6
28	2606.6	4.345	15.3	78	770.6	1.285	8.6
29	2603.7	4.340	14.4	79	762.6	1.271	35.5
30	2483.7	4.140	7.4	80	755.6	1.260	78.2
31	2476.3	4.128	22.9	81	752.7	1.255	39.0
32	2469.3	4.116	22.8	82	748.6	1.248	32.8
33	2461.9	4.104	7.3	83	714.2	1.191	31.2
34	2397.7	3.997	21.4	84	527.9	0.880	10.7
35	2394.7	3.992	23.9	85	520.9	0.868	7.6
36	2393.0	3.989	24.0	86	41.4	0.069	43.2
37	2390.3	3.985	20.0	87	3.2	0.005	20.5
38	2292.9	3.822	28.3	88	-0.3	-0.000	596.1
39	2289.4	3.816	338.2	89	-3.5	-0.006	20.7
40	2283.5	3.807	7.4				
41	2273.3	3.789	25.2				
42	2269.2	3.783	375.1				
43	2258.6	3.765	8.5				
44	2255.7	3.760	13.4				
45	2252.4	3.755	17.2				
46	2248.9	3.749	12.2				
47	2133.0	3.556	21.3				
48	2094.3	3.491	29.2				
49	2065.5	3.443	355.8				
50	1928.5	3.215	9.9				

Figure S32: ^{13}C-NMR Spectrum of (+)-**23** in $CDCl_3$ (150 MHz)

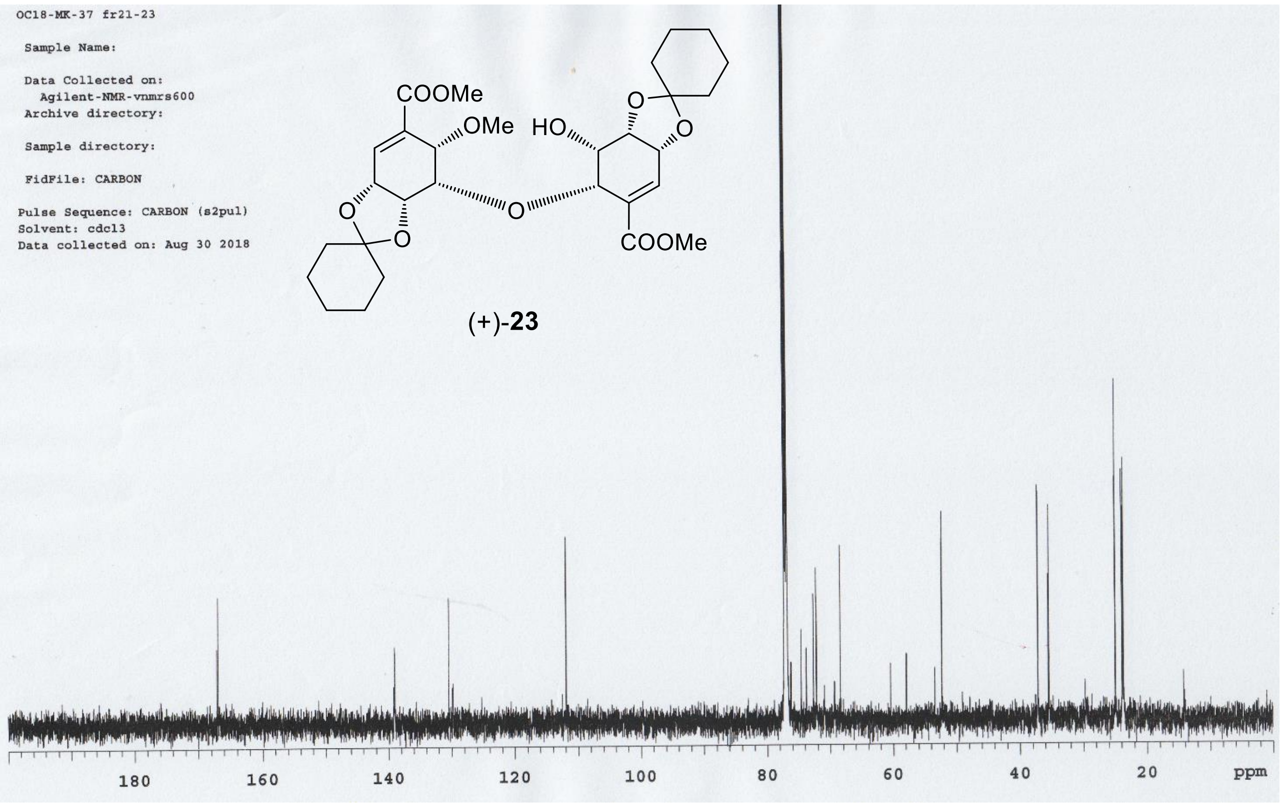

OC18-MK-37 fr21-23

INDEX	FREQUENCY	PPM	HEIGHT
1	25202.2	167.075	15.9
2	25173.3	166.883	26.8
3	20969.1	139.012	16.1
4	19667.5	130.383	26.4
5	19577.3	129.785	8.4
6	16875.8	111.876	39.2
7	16872.3	111.853	20.6
8	11647.3	77.215	2319.7
9	11615.0	77.000	2418.9
10	11582.6	76.785	2144.9
11	11490.1	76.172	12.4
12	11247.4	74.563	19.3
13	11124.8	73.751	15.3
14	10958.4	72.647	26.7
15	10887.9	72.180	32.3
16	10696.0	70.908	7.4
17	10455.5	69.314	8.2
18	10314.5	68.379	37.0
19	9110.0	60.394	12.0
20	8729.7	57.872	14.0
21	8056.9	53.412	11.0
22	7890.4	52.309	38.2
23	7881.2	52.247	44.0
24	5607.4	37.174	49.4
25	5356.6	35.511	30.5
26	5345.0	35.434	45.2
27	4479.2	29.694	8.2
28	3764.8	24.958	71.6
29	3605.3	23.901	52.5
30	3589.1	23.793	44.8
31	3579.8	23.732	41.4
32	3568.3	23.655	55.0
33	2139.5	14.184	10.2

Figure S33: ^{1}H- NMR Spectrum of (-)-**23** in $CDCl_3$ (400 MHz)

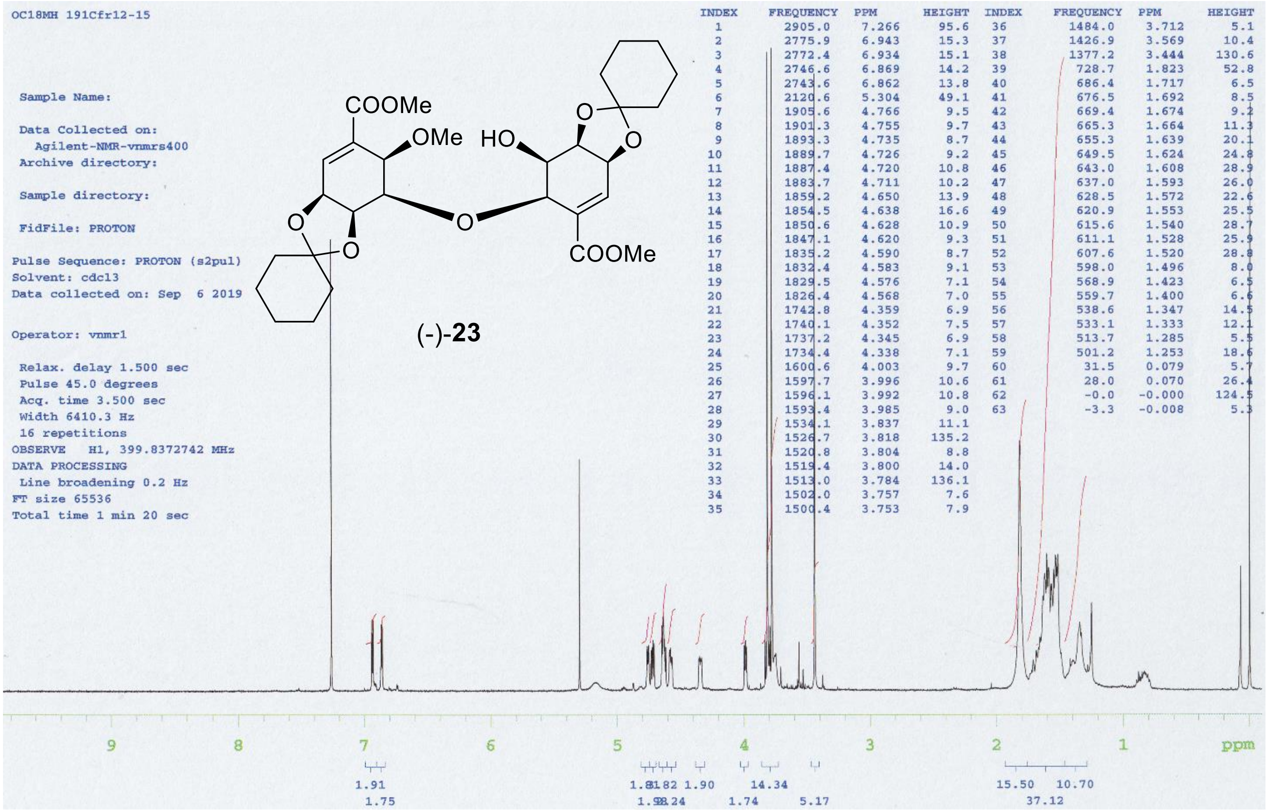

Figure S34: ^{13}C-NMR Spectrum of (-)-**23** in $CDCl_3$ (100 MHz)

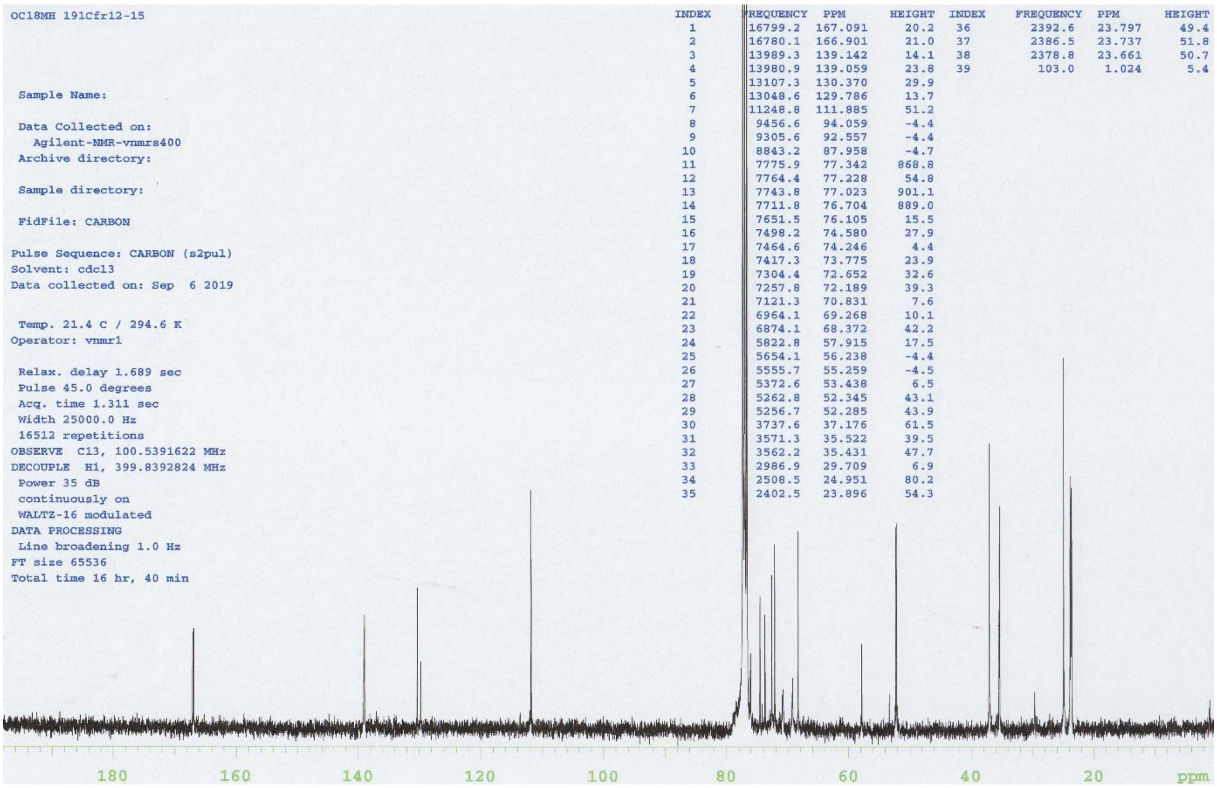

Figure S35: ^{1}H- NMR Spectrum of (+)-**6** in acetone-d$_6$ (400 MHz)

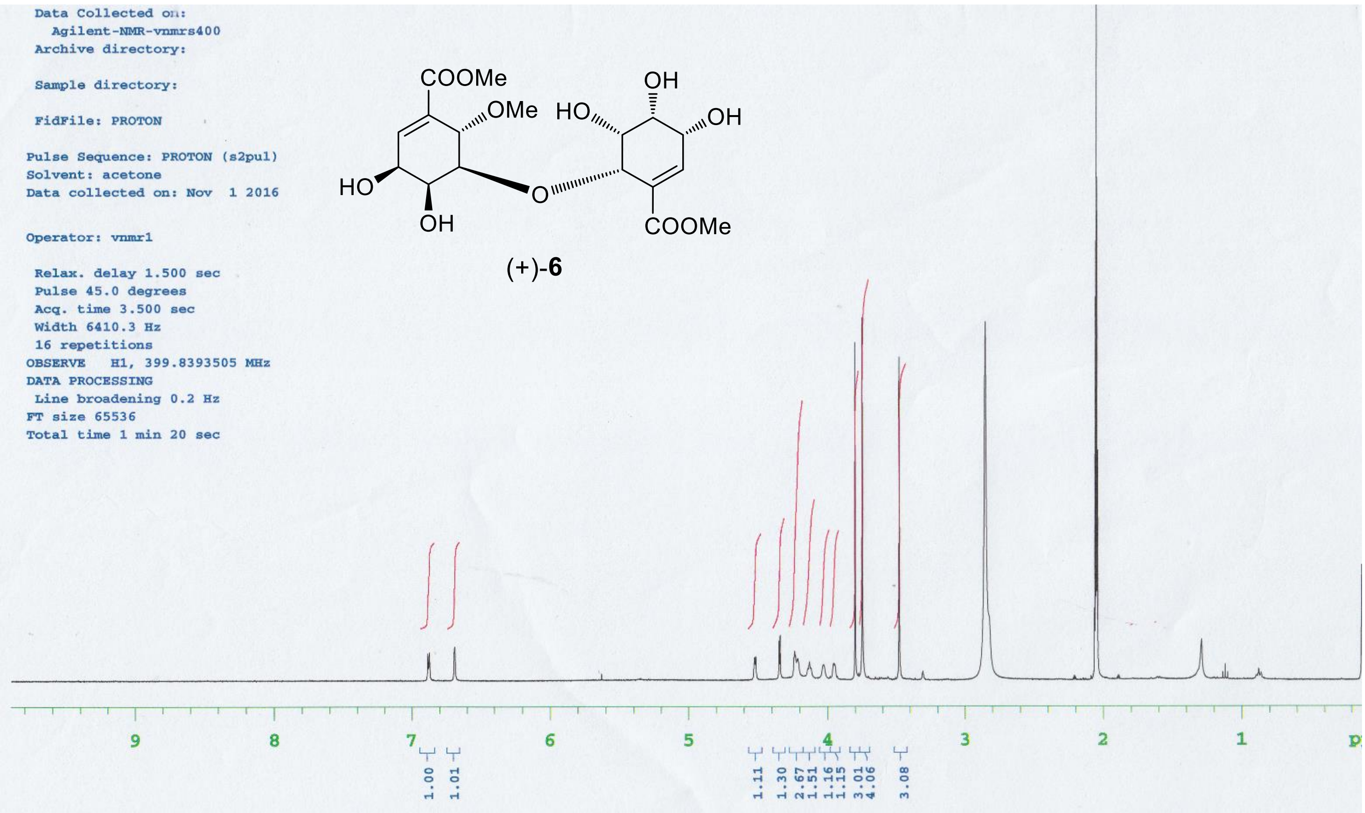

INDEX	FREQUENCY	PPM	HEIGHT
1	2754.2	6.888	30.0
2	2749.7	6.877	30.9
3	2676.4	6.694	39.2
4	1811.3	4.530	26.3
5	1807.4	4.520	27.7
6	1740.3	4.352	46.0
7	1737.2	4.345	52.4
8	1695.1	4.239	34.0
9	1692.7	4.234	29.0
10	1686.7	4.218	25.4
11	1657.3	4.145	13.8
12	1652.6	4.133	21.6
13	1612.7	4.033	18.4
14	1583.4	3.960	20.0
15	1580.3	3.952	19.0
16	1521.0	3.804	390.4
17	1500.1	3.752	419.2
18	1392.3	3.482	373.6
19	1323.6	3.310	10.9
20	1142.8	2.858	414.2
21	828.3	2.072	12.3
22	826.1	2.066	260.6
23	823.8	2.060	507.6
24	821.6	2.055	785.3
25	819.5	2.050	542.2
26	817.3	2.044	265.6
27	516.5	1.292	46.4
28	455.2	1.139	9.9
29	448.2	1.121	19.1
30	441.1	1.103	9.9
31	352.3	0.881	12.9
32	52.6	0.132	133.1
33	49.9	0.125	21.1
34	0.0	0.000	330.6
35	-3.3	-0.008	11.2

Figure S36: ^{13}C-NMR Spectrum of (+)-**6** in acetone-d_6 (100 MHz)

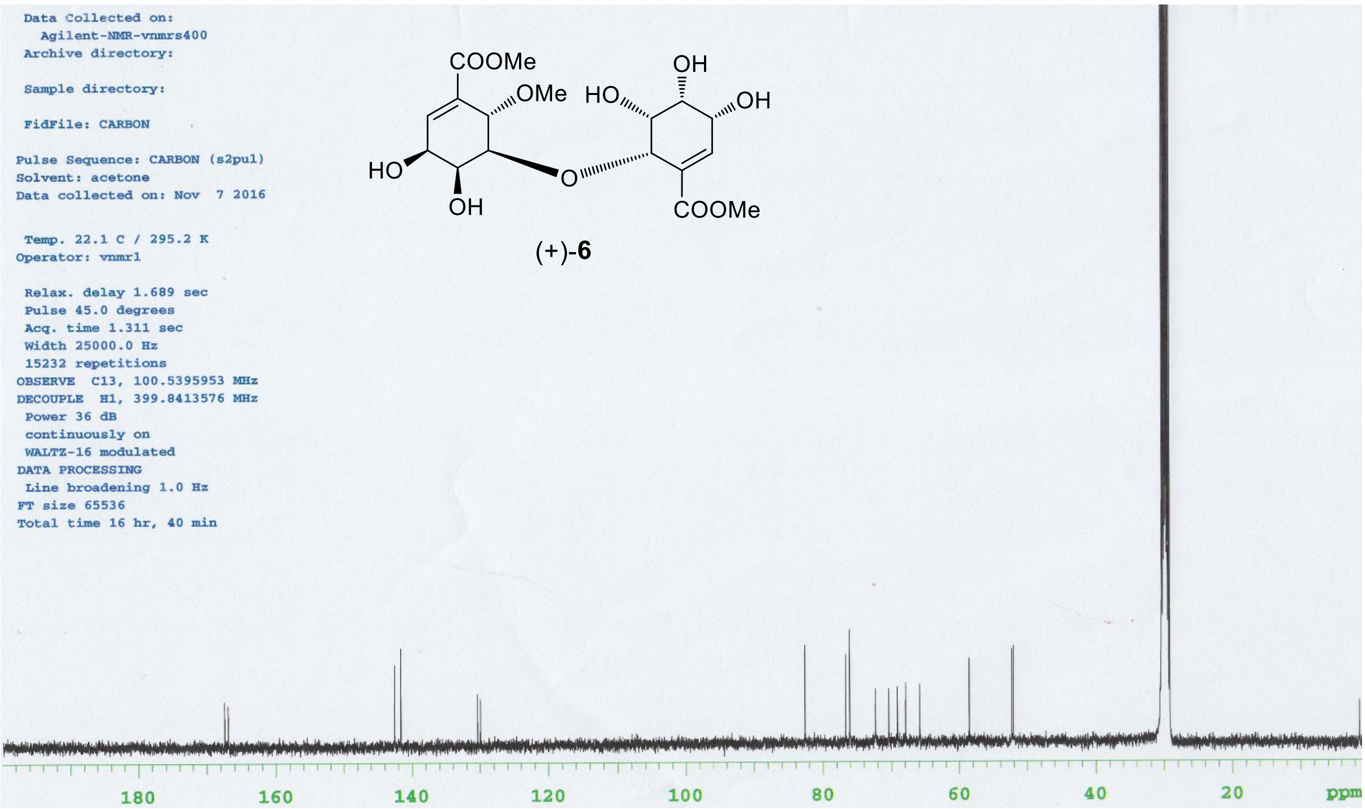

INDEX	FREQUENCY	PPM	HEIGHT
1	16838.4	167.480	9.2
2	16787.3	166.972	8.3
3	14343.6	142.666	19.1
4	14249.7	141.732	23.9
5	13115.2	130.448	11.4
6	13074.0	130.039	9.9
7	8314.1	82.694	23.8
8	7717.4	76.760	21.7
9	7658.7	76.176	27.7
10	7281.0	72.420	12.3
11	7084.2	70.462	12.4
12	6956.8	69.195	12.9
13	6837.0	68.003	13.9
14	6630.2	65.947	13.7
15	5891.7	58.601	20.1
16	5265.4	52.371	22.8
17	5240.9	52.128	23.7
18	3073.4	30.569	14.8
19	3057.4	30.410	435.5
20	3038.3	30.220	1337.8
21	3019.3	30.031	2363.7
22	2999.4	29.833	3048.0
23	2980.3	29.644	2595.9
24	2960.5	29.446	1181.0
25	2941.4	29.257	438.2
26	139.9	1.392	9.0
27	-2.0	-0.020	6.8

Figure S37: ^{1}H- NMR Spectrum of (-)-**6** in acetone-d_6 (600 MHz)

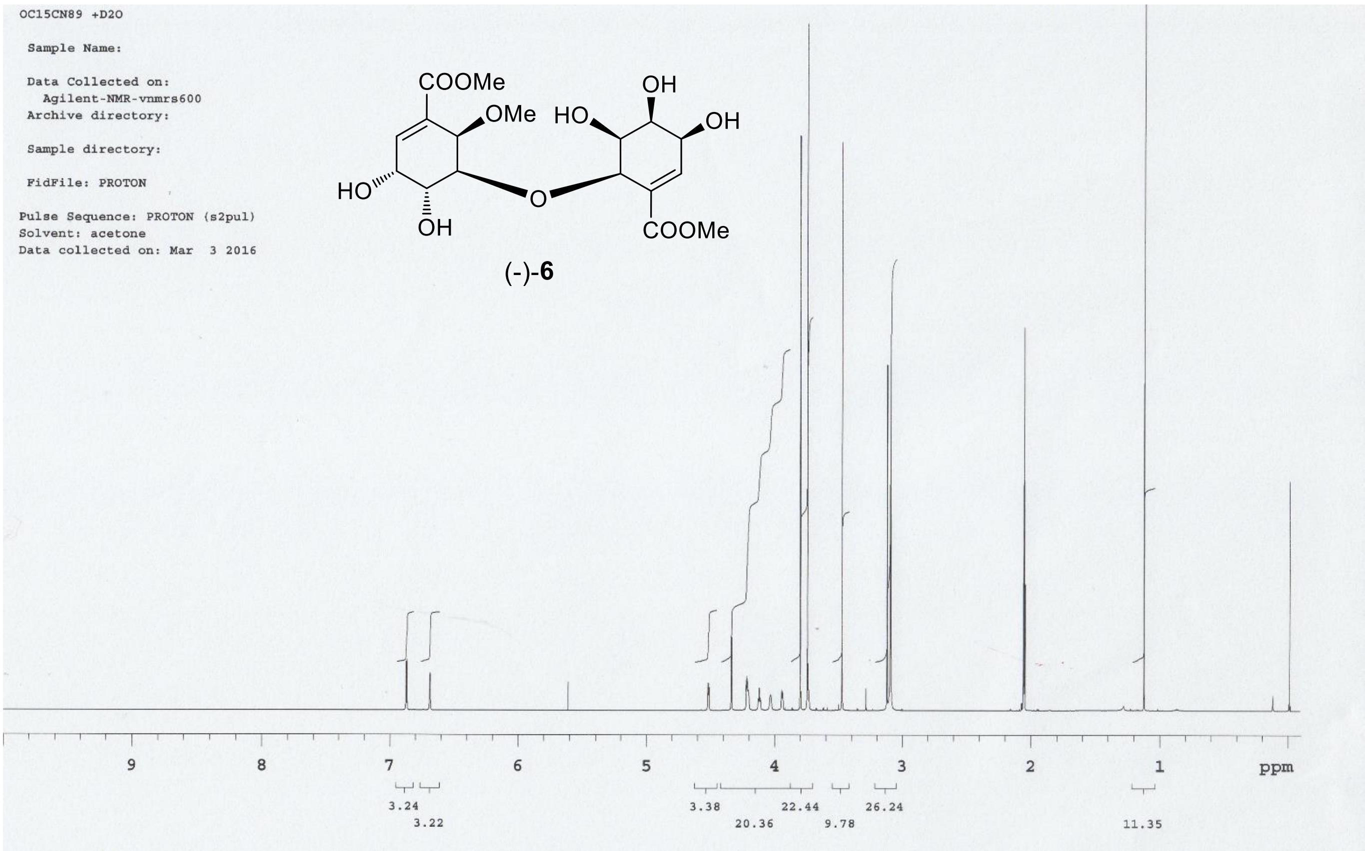

OC15CN89 +D2O

INDEX	FREQUENCY	PPM	HEIGHT
1	4124.3	6.875	85.6
2	4119.6	6.867	89.0
3	4011.9	6.688	64.3
4	4010.7	6.686	65.5
5	3366.6	5.612	50.5
6	2710.2	4.518	48.7
7	2706.1	4.511	49.1
8	2602.5	4.338	117.6
9	2599.3	4.333	129.2
10	2532.1	4.221	45.1
11	2529.8	4.217	60.2
12	2526.8	4.212	56.0
13	2522.4	4.205	37.0
14	2475.5	4.126	21.7
15	2470.5	4.118	40.1
16	2465.8	4.110	23.3
17	2418.3	4.031	28.4
18	2367.2	3.946	33.6
19	2365.4	3.943	35.7
20	2362.2	3.938	33.5
21	2360.4	3.935	29.9
22	2278.3	3.798	1006.7
23	2249.2	3.749	18.2
24	2244.8	3.742	1202.3
25	2239.3	3.733	37.1
26	2083.1	3.472	994.9
27	2077.9	3.464	13.3
28	1971.3	3.286	39.4
29	1873.9	3.124	20.5
30	1872.2	3.121	605.7
31	1869.8	3.117	18.8
32	1856.0	3.094	608.4
33	1243.9	2.074	13.6
34	1241.9	2.070	14.3
35	1239.5	2.066	13.5
36	1236.3	2.061	224.8
37	1233.9	2.057	395.5
38	1231.9	2.053	670.6
39	1229.5	2.050	404.1
40	1227.5	2.046	222.0
41	674.6	1.125	37.0
42	672.9	1.122	2200.2
43	670.5	1.118	27.7
44	70.5	0.117	27.2
45	-4.4	-0.007	12.9
46	-7.6	-0.013	402.2

Figure S38: ^{13}C-NMR Spectrum of (-)-**6** in acetone-d_6 (100 MHz)

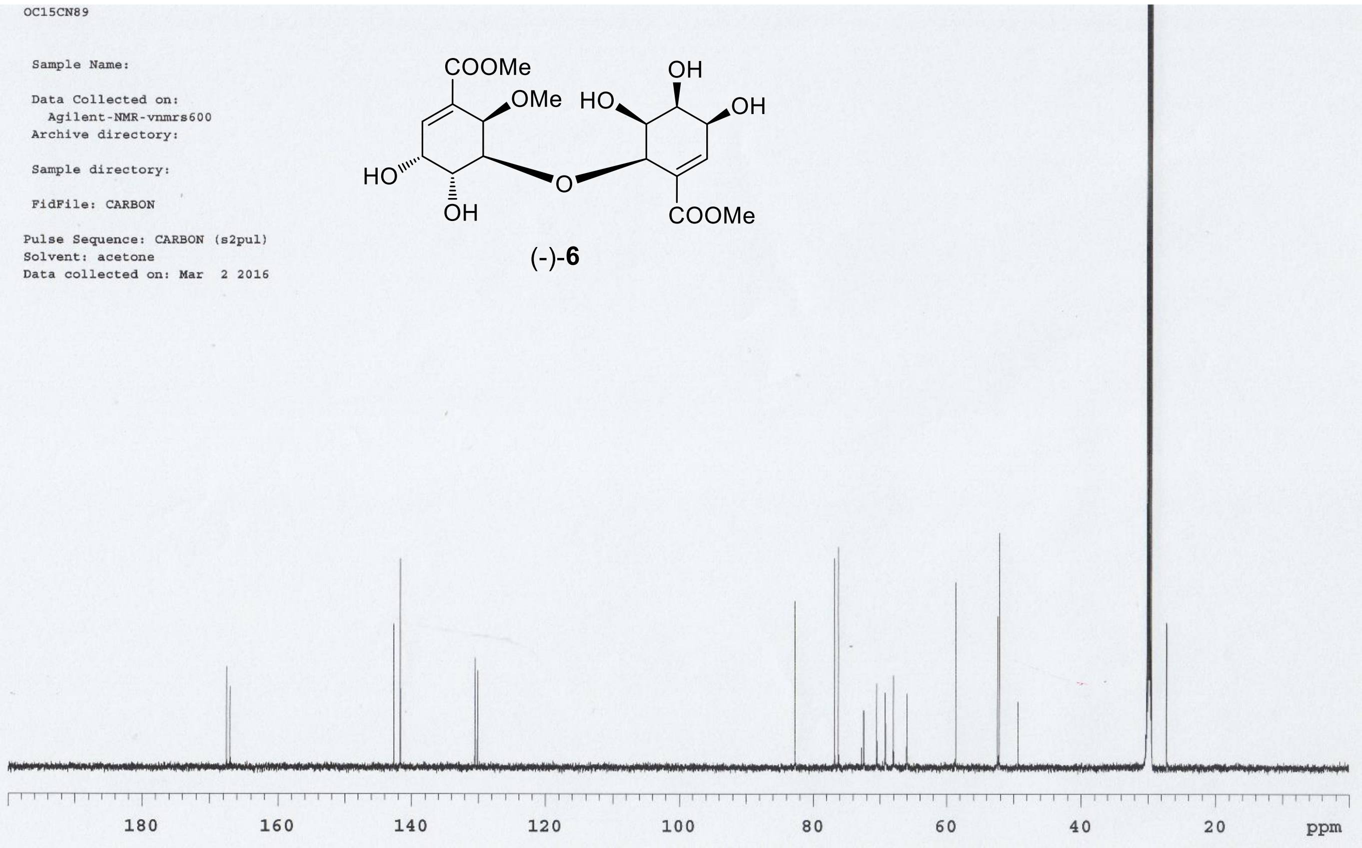

OC15CN89

INDEX	FREQUENCY	PPM	HEIGHT
1	31114.1	206.266	24.2
2	31095.6	206.143	1652.1
3	31074.7	206.005	21.5
4	25264.8	167.489	20.2
5	25187.4	166.976	16.2
6	21517.2	142.645	28.6
7	21372.7	141.687	41.7
8	19682.7	130.483	21.9
9	19626.0	130.108	19.4
10	12477.5	82.718	33.2
11	11580.5	76.771	41.7
12	11493.8	76.196	44.0
13	10980.5	72.794	4.0
14	10925.1	72.426	11.6
15	10631.4	70.479	16.9
16	10438.4	69.200	15.0
17	10259.2	68.012	18.5
18	9952.9	65.981	14.8
19	8842.0	58.617	37.0
20	7901.0	52.379	30.2
21	7864.0	52.133	46.7
22	7439.8	49.321	13.2
23	4576.5	30.339	6.7
24	4559.1	30.224	188.9
25	4539.5	30.094	639.3
26	4519.8	29.964	1166.1
27	4500.2	29.833	1027.0
28	4481.7	29.711	1153.5
29	4462.0	29.580	639.9
30	4442.4	29.450	190.8
31	4110.6	27.251	29.0
32	-1.2	-0.008	6.8

Figure S39: ^{1}H-NMR Spectrum of (+)-**14** in acetone-d_6 (600 MHz)

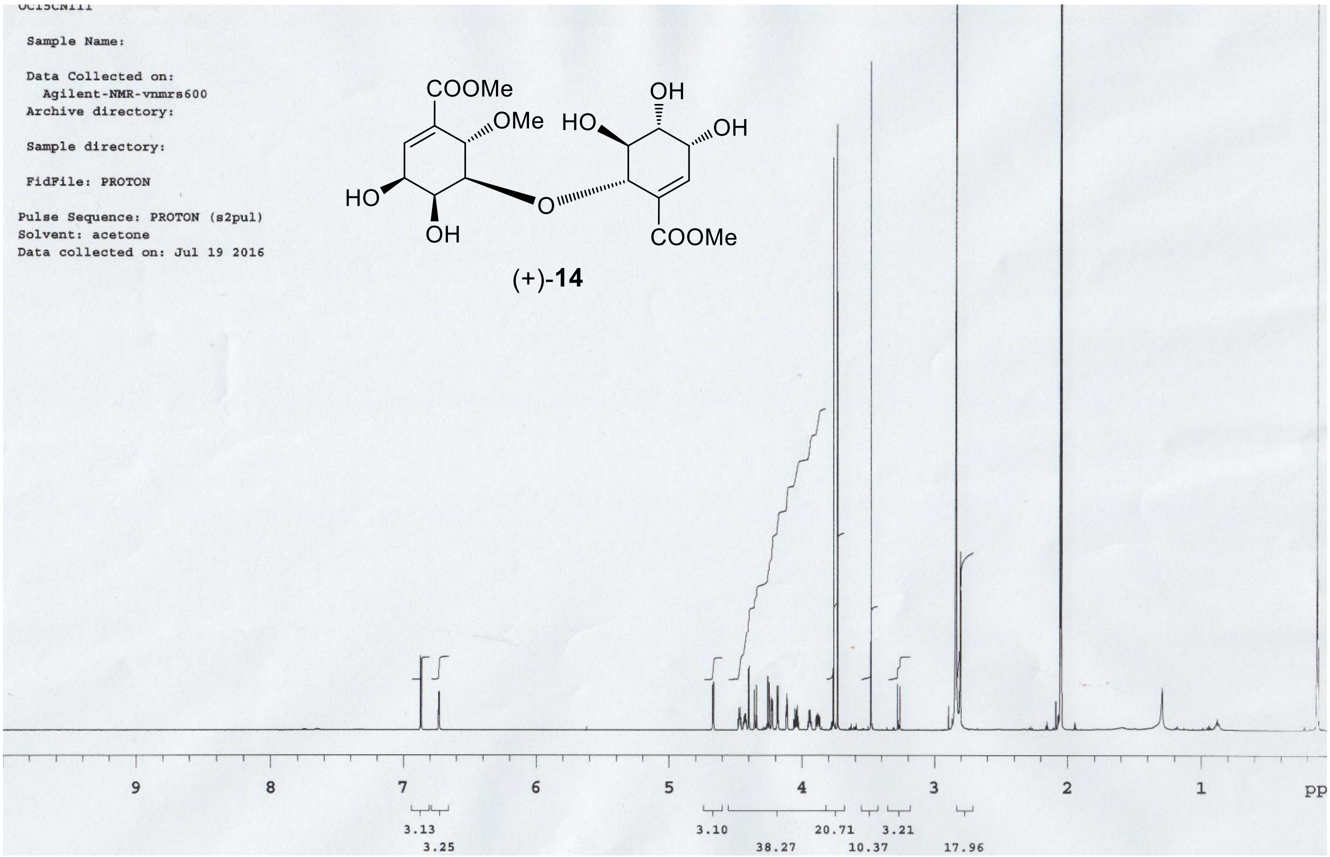

OC15CN111

INDEX	FREQUENCY	PPM	HEIGHT	INDEX	FREQUENCY	PPM	HEIGHT	INDEX	FREQUENCY	PPM	HEIGHT
1	4123.2	6.873	92.2	51	2321.2	3.869	17.4	101	74.3	0.124	189.5
2	4118.5	6.865	88.4	52	2318.8	3.865	16.2	102	73.5	0.122	165.9
3	4040.4	6.735	47.2	53	2265.4	3.776	9.7	103	72.3	0.120	103.9
4	4038.4	6.732	35.0	54	2263.4	3.773	11.2	104	70.5	0.118	12.2
5	4037.5	6.730	48.0	55	2262.5	3.771	7.7	105	69.6	0.116	10.3
6	2801.8	4.671	56.2	56	2260.5	3.768	13.2	106	68.5	0.114	12.7
7	2798.0	4.664	60.5	57	2256.3	3.761	709.0	107	1.9	0.003	21.8
8	2686.8	4.479	14.3	58	2249.3	3.749	9.3	108	-1.4	-0.002	810.7
9	2683.3	4.473	27.6	59	2238.1	3.731	751.1	109	-4.9	-0.008	24.3
10	2681.2	4.469	20.2	60	2174.8	3.625	8.1				
11	2679.5	4.467	18.9	61	2153.6	3.590	8.4				
12	2677.7	4.464	29.3	62	2087.3	3.479	828.8				
13	2673.9	4.457	15.6	63	2081.2	3.469	8.0				
14	2663.6	4.440	9.5	64	1968.2	3.281	57.3				
15	2660.4	4.435	16.7	65	1957.3	3.263	54.3				
16	2656.6	4.428	20.6	66	1735.2	2.892	30.2				
17	2653.1	4.423	20.7	67	1719.1	2.866	15.0				
18	2649.3	4.416	12.2	68	1717.0	2.862	7.7				
19	2640.7	4.402	76.4	69	1702.9	2.839	14021.9				
20	2637.5	4.397	79.7	70	1689.7	2.817	21.6				
21	2614.0	4.357	50.4	71	1688.0	2.814	23.3				
22	2612.0	4.354	12.2	72	1683.0	2.805	222.0				
23	2604.7	4.342	55.8	73	1292.7	2.155	11.3				
24	2602.3	4.338	12.7	74	1252.5	2.088	36.5				
25	2559.2	4.266	7.9	75	1251.9	2.087	13.3				
26	2553.6	4.257	67.3	76	1251.3	2.086	12.8				
27	2551.2	4.253	7.8	77	1250.7	2.085	10.1				
28	2546.3	4.244	59.3	78	1242.8	2.072	17.8				
29	2535.7	4.227	40.1	79	1240.8	2.068	20.8				
30	2532.8	4.222	38.2	80	1238.4	2.064	18.2				
31	2511.6	4.187	54.8	81	1235.2	2.059	660.4				
32	2506.9	4.179	55.6	82	1232.8	2.055	1139.5				
33	2470.3	4.118	31.9	83	1230.8	2.052	1977.5				
34	2467.6	4.113	45.0	84	1228.4	2.048	1140.6				
35	2464.7	4.108	31.8	85	1226.4	2.044	658.3				
36	2436.2	4.061	13.7	86	1223.1	2.039	9.9				
37	2431.2	4.053	27.3	87	1168.9	1.948	7.9				
38	2425.9	4.044	25.0	88	1166.8	1.945	10.4				
39	2420.7	4.035	31.3	89	1164.5	1.941	7.8				
40	2415.7	4.027	17.3	90	782.1	1.304	17.2				
41	2371.7	3.953	10.9	91	773.9	1.290	53.6				
42	2367.0	3.946	25.6	92	563.8	0.940	7.0				
43	2361.7	3.937	25.3	93	535.6	0.893	7.8				
44	2357.3	3.929	10.8	94	533.9	0.890	7.5				
45	2335.9	3.894	18.2	95	530.0	0.884	9.1				
46	2333.5	3.890	18.1	96	528.9	0.882	10.6				
47	2330.9	3.885	16.7	97	526.8	0.878	14.7				
48	2328.5	3.882	21.0	98	523.6	0.873	9.9				
49	2326.2	3.878	19.2	99	520.4	0.867	8.5				
50	2324.1	3.874	18.6	100	76.1	0.127	1132.5				

Figure S40: ^{13}C-NMR Spectrum of (+)-**14** in acetone-d_6 (100 MHz)

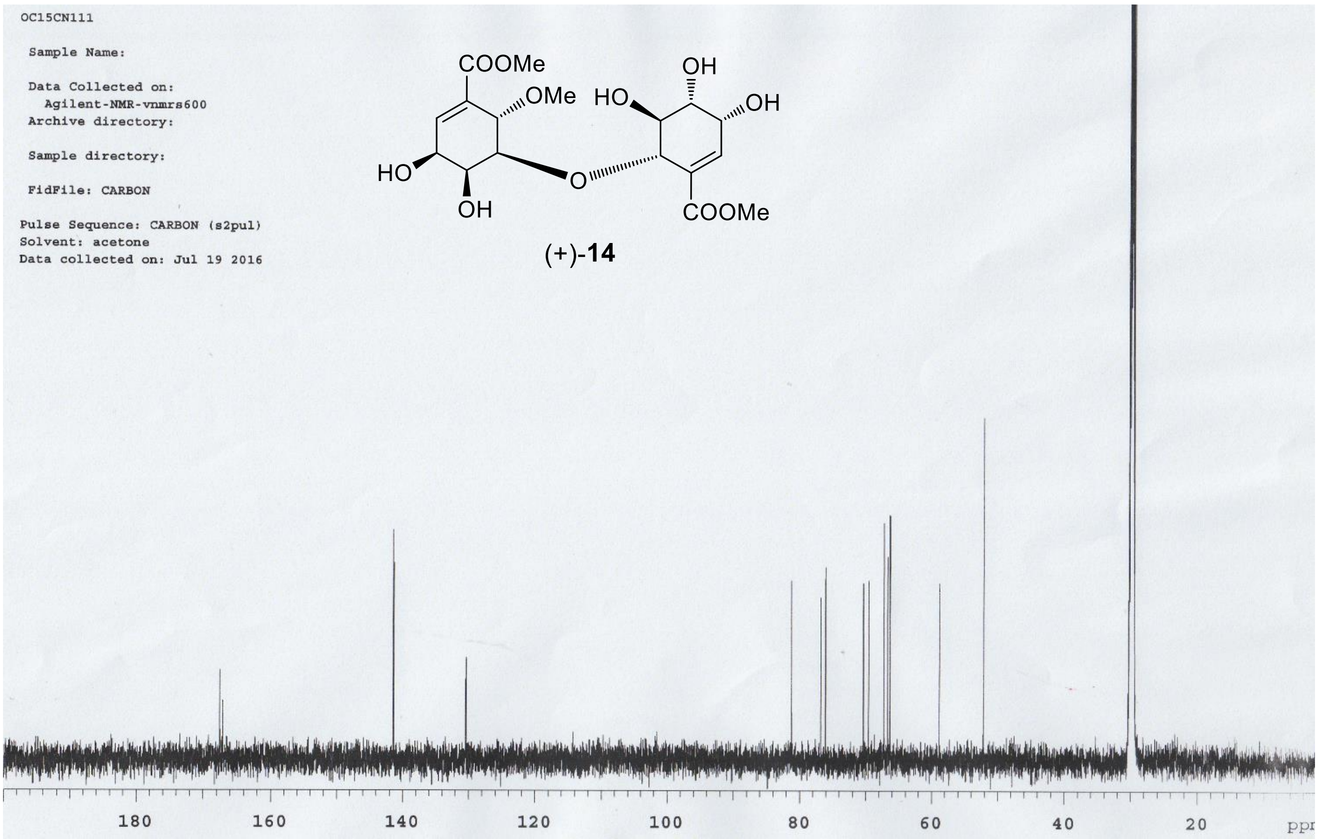

OC15CN111

INDEX	FREQUENCY	PPM	HEIGHT
1	31164.9	206.603	12.2
2	31121.0	206.312	149.3
3	31101.3	206.181	11222.3
4	31080.5	206.044	148.2
5	25263.7	167.482	19.2
6	25197.8	167.045	13.0
7	21339.2	141.465	47.4
8	21320.7	141.342	40.8
9	19682.7	130.483	17.3
10	19665.3	130.368	21.7
11	12251.0	81.216	37.3
12	11584.0	76.794	33.9
13	11469.5	76.035	40.0
14	10608.3	70.326	36.8
15	10491.6	69.552	37.4
16	10143.6	67.246	49.0
17	10055.8	66.663	42.1
18	10004.9	66.326	50.5
19	8867.4	58.785	36.7
20	7864.1	52.134	70.2
21	4577.6	30.347	33.5
22	4559.1	30.224	938.6
23	4540.6	30.101	3422.1
24	4521.0	29.971	7618.6
25	4501.3	29.841	8161.9
26	4481.7	29.711	5330.2
27	4463.2	29.588	3519.6
28	4443.5	29.458	1282.5
29	4421.6	29.312	14.5
30	212.7	1.410	141.5

Figure S41: ^{1}H- NMR Spectrum of (-)-**14** in acetone-d_6 (600 MHz)

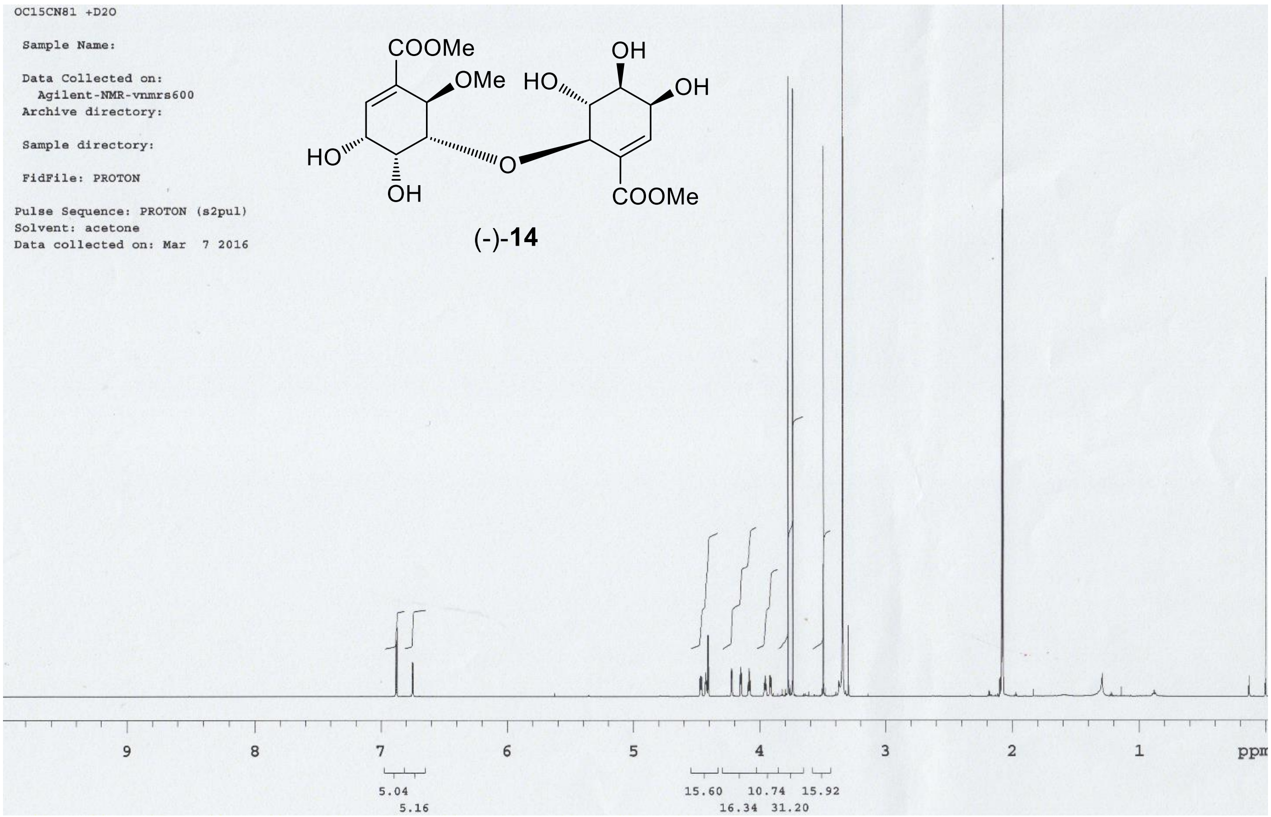

OC15CN81 +D2O

INDEX	FREQUENCY	PPM	HEIGHT	INDEX	FREQUENCY	PPM	HEIGHT
1	4128.7	6.882	98.7	51	1241.8	2.070	458.2
2	4124.0	6.874	107.0	52	1099.2	1.832	11.3
3	4050.6	6.752	53.7	53	773.5	1.289	36.6
4	4048.0	6.748	52.6	54	684.0	1.140	15.4
5	2683.2	4.473	28.6	55	78.6	0.131	32.7
6	2680.0	4.467	32.6	56	3.5	0.006	15.0
7	2677.6	4.463	32.1	57	-0.0	-0.000	649.5
8	2674.4	4.458	30.9	58	-3.2	-0.005	20.2
9	2658.0	4.431	23.1				
10	2654.7	4.425	37.8				
11	2651.8	4.420	23.0				
12	2645.9	4.411	95.2				
13	2642.7	4.405	95.5				
14	2533.8	4.224	44.3				
15	2530.9	4.219	42.5				
16	2491.6	4.153	37.2				
17	2488.9	4.149	46.6				
18	2486.0	4.144	35.9				
19	2454.9	4.092	23.3				
20	2449.9	4.084	44.8				
21	2445.2	4.076	25.0				
22	2378.3	3.965	21.4				
23	2373.6	3.957	32.5				
24	2368.6	3.948	19.5				
25	2352.8	3.922	33.5				
26	2350.4	3.918	33.3				
27	2347.8	3.914	32.0				
28	2345.5	3.910	28.9				
29	2292.6	3.822	11.1				
30	2278.8	3.799	11.6				
31	2264.5	3.775	959.5				
32	2262.4	3.771	26.5				
33	2254.8	3.759	18.0				
34	2245.4	3.743	11.2				
35	2241.9	3.737	940.7				
36	2238.1	3.731	14.7				
37	2104.0	3.507	12.4				
38	2096.9	3.495	852.2				
39	2089.0	3.482	15.2				
40	2024.7	3.375	25.1				
41	2022.1	3.371	23.1				
42	2005.9	3.344	2908.0				
43	1978.9	3.299	110.1				
44	1258.3	2.097	27.6				
45	1256.2	2.094	30.4				
46	1253.9	2.090	28.9				
47	1250.6	2.085	461.1				
48	1248.6	2.081	754.4				
49	1246.2	2.077	1378.9				
50	1243.9	2.073	776.1				

Figure S42: ^{13}C-NMR Spectrum of (-)-**14** in acetone-d_6 (150 MHz)

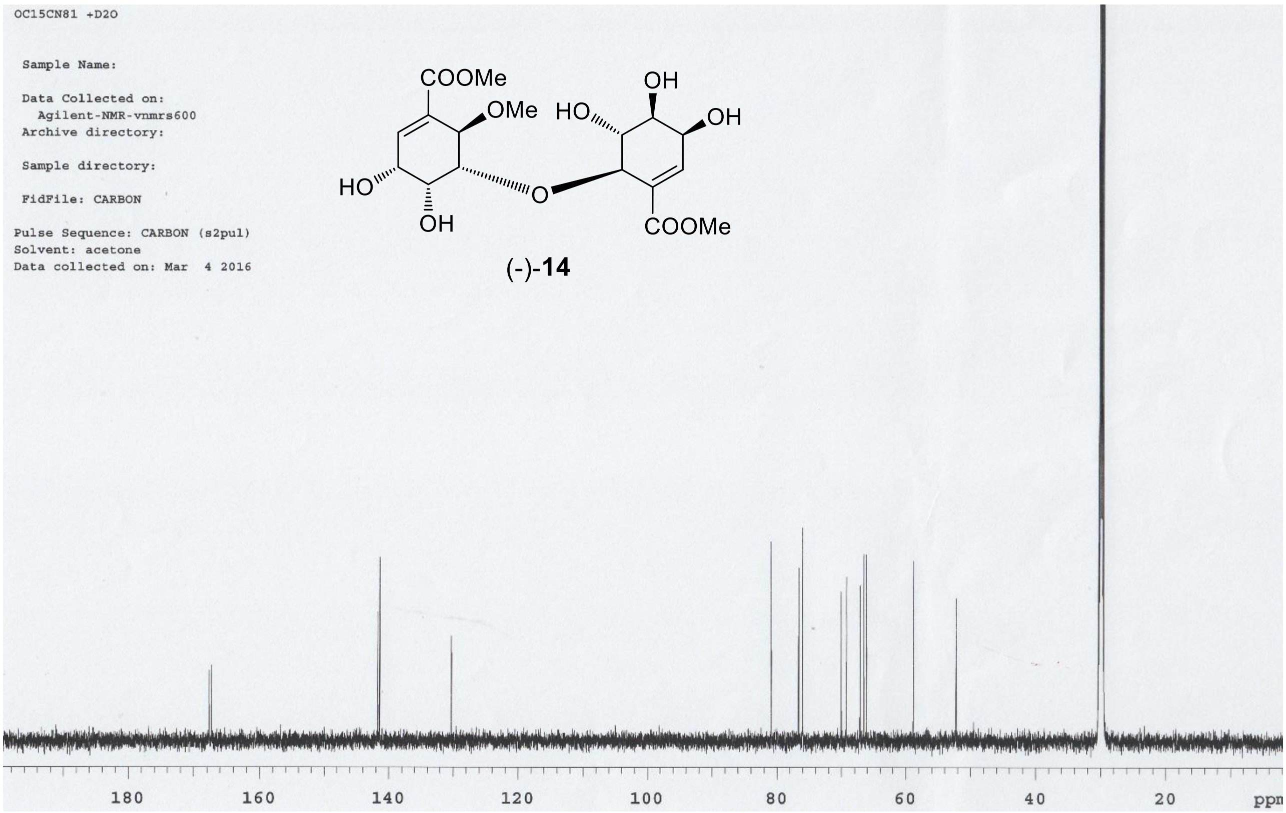

OC15CN81 +D2O

INDEX	FREQUENCY	PPM	HEIGHT
1	31305.9	207.538	57.1
2	31286.3	207.408	4208.6
3	31265.5	207.270	58.3
4	25282.2	167.604	14.8
5	25227.9	167.244	15.9
6	21373.9	141.695	26.9
7	21333.4	141.426	38.2
8	19663.0	130.353	21.9
9	19652.6	130.284	18.5
10	12201.3	80.886	41.4
11	11556.2	76.610	36.0
12	11463.7	75.997	44.3
13	10563.2	70.027	31.1
14	10444.2	69.238	34.3
15	10127.4	67.138	32.3
16	10035.0	66.525	38.9
17	9977.2	66.142	38.8
18	8872.1	58.816	37.4
19	7882.6	52.256	28.9
20	7874.5	52.202	29.7
21	4585.7	30.400	16.3
22	4568.4	30.285	479.1
23	4548.7	30.155	1609.1
24	4529.1	30.025	3033.9
25	4509.4	29.895	2862.9
26	4490.9	29.772	2780.6
27	4471.3	29.642	1606.1
28	4451.6	29.511	518.8
29	0.0	0.000	14.3

Figure S43: ^{1}H- NMR Spectrum of (+)-**7** in acetone-d$_6$ (400 MHz)

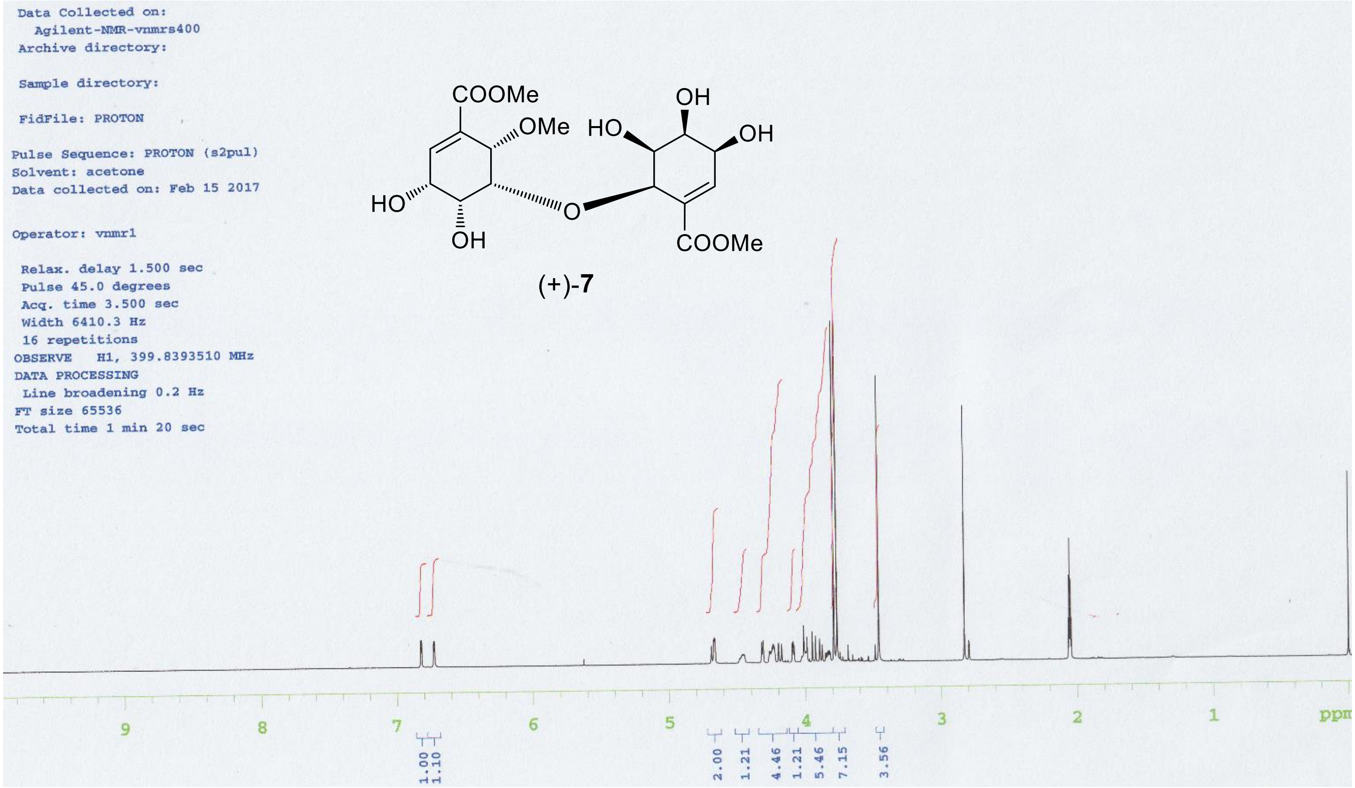

INDEX	FREQUENCY	PPM	HEIGHT	INDEX	FREQUENCY	PPM	HEIGHT	INDEX	FREQUENCY	PPM	HEIGHT
1	2730.5	6.829	33.2	36	1639.9	4.101	25.3	71	1385.6	3.465	393.1
2	2729.2	6.826	37.1	37	1637.6	4.096	27.2	72	1132.3	2.832	349.8
3	2728.0	6.823	37.2	38	1636.0	4.092	26.1	73	1119.8	2.801	21.9
4	2726.6	6.819	33.3	39	1633.9	4.086	23.1	74	1118.8	2.798	27.5
5	2693.0	6.735	32.2	40	1611.4	4.030	8.1	75	1117.8	2.796	22.0
6	2691.6	6.732	34.9	41	1610.2	4.027	8.1	76	825.5	2.065	57.0
7	2690.4	6.729	36.0	42	1606.1	4.017	50.9	77	823.4	2.059	115.1
8	2689.3	6.726	32.4	43	1601.8	4.006	14.3	78	821.2	2.054	166.1
9	2250.3	5.628	8.4	44	1600.6	4.003	13.4	79	819.1	2.049	108.6
10	1875.5	4.691	24.4	45	1599.4	4.000	14.4	80	816.7	2.043	57.3
11	1869.2	4.675	33.5	46	1598.5	3.998	14.7	81	3.1	0.008	8.2
12	1867.2	4.670	35.4	47	1596.3	3.992	35.0	82	-0.0	-0.000	253.4
13	1865.1	4.665	34.8	48	1580.8	3.954	42.4	83	-3.3	-0.008	7.6
14	1791.5	4.481	7.2	49	1571.1	3.929	35.9				
15	1790.2	4.477	7.5	50	1559.1	3.899	32.0				
16	1786.5	4.468	11.5	51	1551.7	3.881	24.2				
17	1785.1	4.464	12.2	52	1542.1	3.857	11.4				
18	1782.7	4.459	11.8	53	1540.0	3.851	11.5				
19	1781.6	4.456	11.9	54	1538.0	3.847	12.2				
20	1780.2	4.452	12.1	55	1535.7	3.841	13.8				
21	1728.4	4.323	27.5	56	1533.9	3.836	15.0				
22	1724.4	4.313	30.0	57	1531.7	3.831	14.5				
23	1707.0	4.269	13.0	58	1529.8	3.826	14.8				
24	1705.9	4.266	14.3	59	1527.6	3.821	11.9				
25	1704.3	4.262	15.7	60	1516.9	3.794	469.0				
26	1702.7	4.259	15.4	61	1507.9	3.771	469.7				
27	1701.4	4.255	15.7	62	1499.3	3.750	13.3				
28	1699.8	4.251	16.4	63	1498.7	3.748	11.7				
29	1698.6	4.248	20.1	64	1490.9	3.729	6.8				
30	1697.2	4.245	22.4	65	1475.2	3.690	23.6				
31	1696.1	4.242	24.0	66	1461.7	3.656	8.8				
32	1694.5	4.238	25.4	67	1436.5	3.593	6.1				
33	1691.6	4.231	21.4	68	1416.7	3.543	7.8				
34	1679.2	4.200	27.4	69	1398.1	3.497	6.3				
35	1670.4	4.178	23.7	70	1396.0	3.491	23.4				

Figure S44: ^{13}C-NMR Spectrum of (+)-**7** in acetone-d_6 (100 MHz)

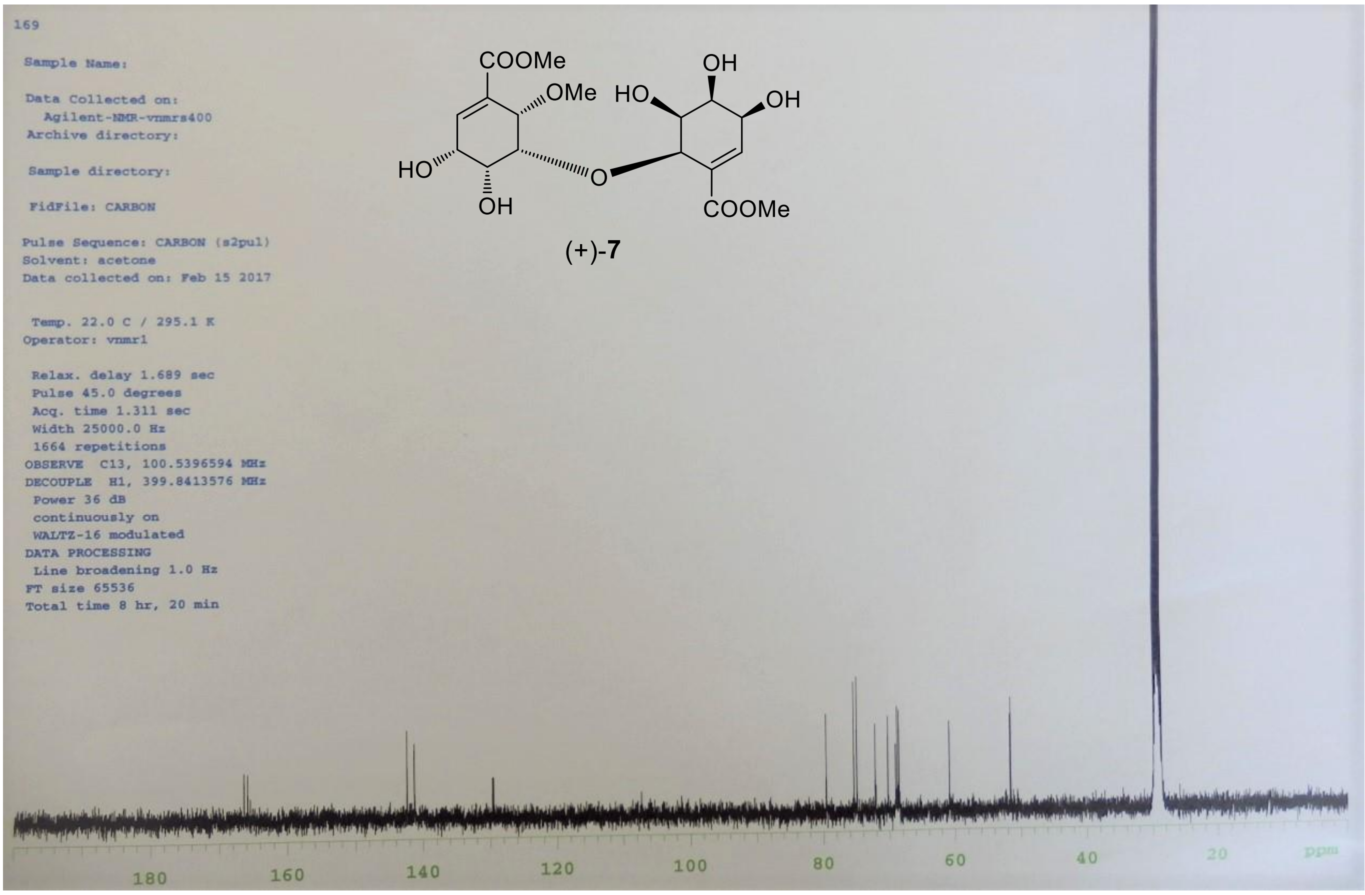

INDEX	FREQUENCY	PPM	HEIGHT
1	16730.8	166.410	8.8
2	16679.7	165.901	8.2
3	14319.9	142.430	16.5
4	14210.0	141.338	13.8
5	13037.4	129.674	6.9
6	13022.1	129.522	7.1
7	8003.5	79.606	18.5
8	7584.7	75.440	25.3
9	7538.1	74.977	26.0
10	7259.7	72.207	16.4
11	7065.1	70.272	17.9
12	6948.4	69.111	12.1
13	6924.0	68.868	20.0
14	6897.3	68.602	19.1
15	6124.4	60.915	17.1
16	5198.2	51.703	17.7
17	5186.8	51.589	21.6
18	2993.3	29.772	208.6
19	2973.5	29.575	576.8
20	2954.4	29.385	1273.6
21	2935.3	29.196	1383.2
22	2915.5	28.998	1196.6
23	2896.4	28.809	635.1
24	2877.3	28.619	186.1

Figure S45: ^{1}H- NMR Spectrum of (-)-**7** in acetone-d_6 (400 MHz)

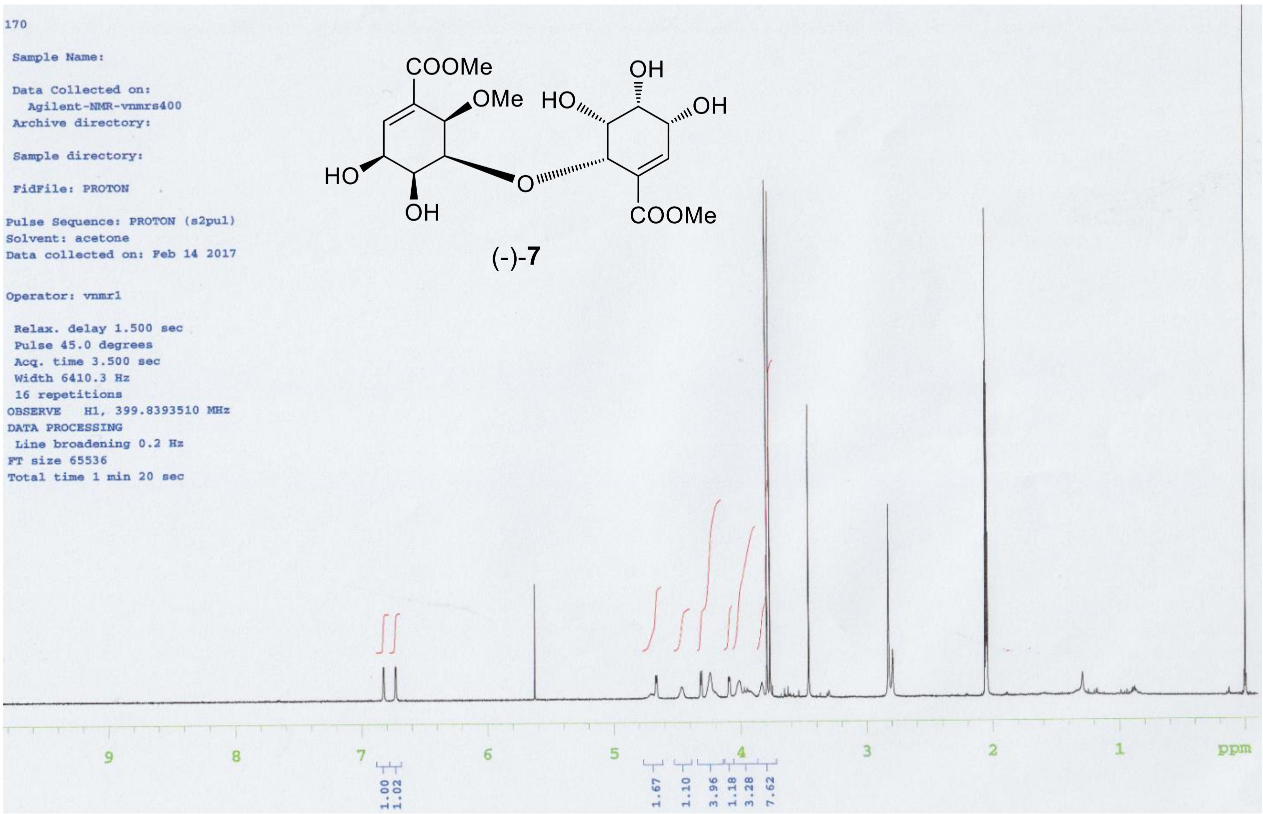

OC18-MK-69

INDEX	FREQUENCY	PPM	HEIGHT	INDEX	FREQUENCY	PPM	HEIGHT	INDEX	FREQUENCY	PPM	HEIGHT	INDEX	FREQUENCY	PPM	HEIGHT
1	4033.3	6.723	27.8	51	2436.7	4.062	22.7	101	1240.7	2.068	41.5	151	528.2	0.880	16.8
2	4032.4	6.722	26.9	52	2429.4	4.050	6.7	102	1239.8	2.067	10.7	152	525.0	0.875	12.8
3	4029.2	6.716	27.9	53	2369.5	3.950	31.7	103	1237.1	2.062	603.4	153	521.1	0.869	10.2
4	4028.3	6.715	26.4	54	2368.9	3.949	34.1	104	1235.1	2.059	1095.3	154	518.5	0.864	7.4
5	4021.0	6.703	28.6	55	2361.0	3.936	30.9	105	1232.7	2.055	1817.9	155	513.2	0.856	4.0
6	4019.8	6.701	29.7	56	2358.4	3.931	30.2	106	1230.7	2.051	1083.7	156	77.5	0.129	87.4
7	4018.3	6.698	29.4	57	2355.4	3.926	5.3	107	1228.3	2.048	606.1	157	75.7	0.126	11.3
8	4017.2	6.696	28.9	58	2351.9	3.921	33.8	108	1225.1	2.042	8.4	158	75.1	0.125	9.8
9	3462.3	5.771	3.5	59	2348.7	3.915	4.9	109	1220.7	2.035	3.3	159	73.7	0.123	8.9
10	3374.0	5.624	578.8	60	2333.1	3.889	3.2	110	1218.6	2.031	3.2	160	69.8	0.116	3.6
11	3282.1	5.471	3.1	61	2312.9	3.855	3.6	111	1216.3	2.028	3.4	161	-0.0	-0.000	58.1
12	2717.8	4.530	22.1	62	2268.0	3.781	10.7	112	1180.5	1.968	30.5	162	-3.2	-0.005	3.3
13	2714.0	4.524	22.9	63	2264.5	3.775	687.3	113	1173.2	1.956	3.8				
14	2642.1	4.404	26.4	64	2260.9	3.769	616.6	114	1170.8	1.952	6.4				
15	2637.4	4.396	29.3	65	2257.4	3.763	5.2	115	1168.8	1.948	10.4				
16	2626.3	4.378	46.8	66	2246.6	3.745	11.1	116	1166.4	1.944	6.3				
17	2622.5	4.372	53.6	67	2238.3	3.731	45.1	117	1164.4	1.941	3.6				
18	2618.9	4.366	7.5	68	2228.1	3.714	42.4	118	995.6	1.660	3.5				
19	2614.8	4.359	13.7	69	2225.1	3.709	9.8	119	994.5	1.658	3.3				
20	2611.3	4.353	13.7	70	2222.2	3.704	14.0	120	968.6	1.615	6.3				
21	2607.8	4.347	16.4	71	2217.8	3.697	13.7	121	963.9	1.607	5.8				
22	2603.7	4.340	8.8	72	2215.2	3.693	9.1	122	961.0	1.602	4.5				
23	2543.5	4.240	9.9	73	2210.8	3.685	8.5	123	955.7	1.593	5.9				
24	2539.1	4.233	12.5	74	2189.3	3.650	3.3	124	848.0	1.414	4.4				
25	2535.9	4.227	14.1	75	2176.7	3.628	12.2	125	822.5	1.371	5.9				
26	2531.5	4.220	13.9	76	2137.4	3.563	480.1	126	815.5	1.359	5.8				
27	2527.4	4.213	9.6	77	2104.5	3.508	6.3	127	808.7	1.348	7.6				
28	2511.0	4.186	6.8	78	1989.8	3.317	9.8	128	800.2	1.334	8.3				
29	2506.3	4.178	9.4	79	1984.5	3.308	10.9	129	783.8	1.307	24.8				
30	2505.1	4.176	9.6	80	1745.4	2.909	9.7	130	775.6	1.293	64.1				
31	2499.5	4.167	10.0	81	1730.1	2.884	4.6	131	760.9	1.268	5.1				
32	2491.9	4.154	35.4	82	1712.5	2.855	4409.9	132	727.4	1.213	12.6				
33	2484.8	4.142	32.1	83	1692.8	2.822	106.7	133	720.4	1.201	19.8				
34	2483.4	4.140	15.7	84	1677.0	2.795	3.5	134	713.1	1.189	9.2				
35	2481.9	4.137	10.6	85	1395.6	2.326	3.7	135	711.9	1.187	3.6				
36	2480.4	4.135	9.6	86	1373.0	2.289	3.5	136	706.9	1.178	5.5				
37	2479.3	4.133	10.5	87	1365.7	2.276	5.5	137	707.5	1.179	5.6				
38	2477.5	4.130	7.2	88	1358.0	2.264	3.1	138	677.0	1.128	6.1				
39	2475.7	4.127	6.9	89	1299.1	2.165	3.6	139	592.7	0.988	5.0				
40	2474.9	4.125	6.8	90	1297.0	2.162	6.6	140	572.8	0.955	3.6				
41	2472.8	4.122	11.0	91	1294.6	2.158	10.0	141	565.5	0.943	6.2				
42	2471.6	4.120	11.4	92	1292.6	2.155	6.6	142	557.8	0.930	3.6				
43	2469.9	4.117	10.9	93	1290.2	2.151	3.7	143	550.5	0.918	3.7				
44	2468.7	4.115	13.2	94	1265.6	2.110	3.5	144	548.4	0.914	5.2				
45	2467.2	4.113	9.3	95	1254.4	2.091	39.6	145	543.4	0.906	4.3				
46	2465.5	4.110	8.9	96	1253.9	2.090	26.0	146	537.3	0.896	9.7				
47	2464.3	4.108	8.3	97	1245.9	2.077	12.5	147	535.2	0.892	8.8				
48	2442.0	4.071	17.4	98	1245.1	2.075	40.4	148	531.7	0.886	12.3				
49	2439.9	4.067	18.9	99	1242.7	2.072	49.0	149	530.5	0.884	14.7				
50	2438.2	4.064	19.4	100	1241.8	2.070	13.5	150	529.4	0.882	15.4				

Figure S46: ^{13}C-NMR Spectrum of (-)-**7** in acetone-d_6 (100 MHz)

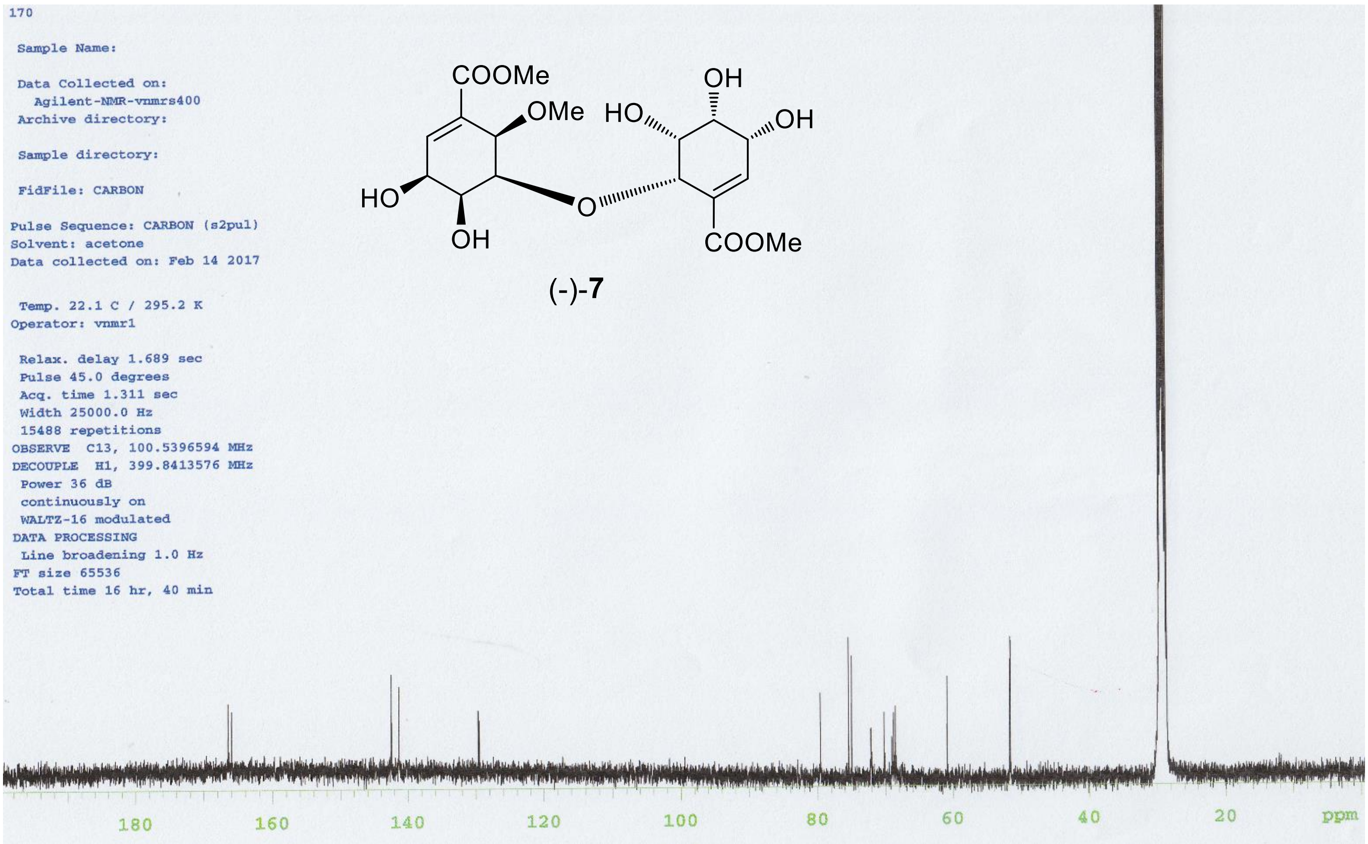

INDEX	FREQUENCY	PPM	HEIGHT
1	16731.2	166.414	18.4
2	16680.9	165.913	16.8
3	14321.1	142.442	25.4
4	14212.0	141.357	22.6
5	13037.8	129.679	16.6
6	13022.6	129.527	14.0
7	8004.7	79.618	20.5
8	7582.8	75.421	34.0
9	7538.6	74.981	29.5
10	7257.0	72.181	11.8
11	7062.5	70.246	15.7
12	6947.3	69.100	9.8
13	6923.6	68.865	15.7
14	6896.9	68.599	17.4
15	6124.8	60.920	24.6
16	5198.6	51.707	28.7
17	5187.2	51.593	34.2
18	3009.0	29.928	28.8
19	2993.7	29.777	744.9
20	2973.9	29.579	1932.1
21	2954.8	29.390	4394.5
22	2935.8	29.200	4971.1
23	2915.9	29.003	4026.8
24	2896.8	28.813	2216.8
25	2877.8	28.623	680.2

Figure S47: ^{1}H- NMR Spectrum of (-)-**18** in acetone-d_6 (400 MHz)

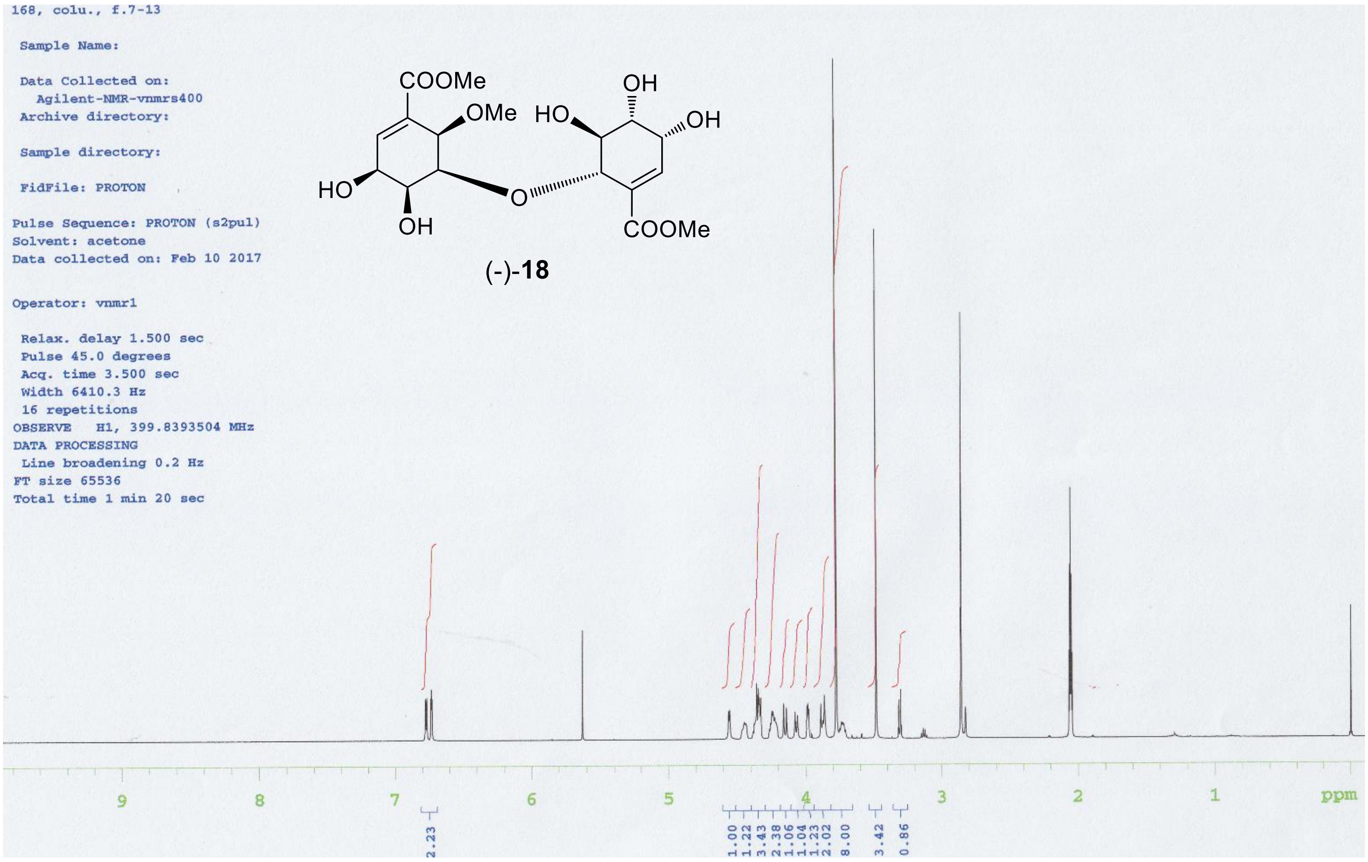

INDEX	FREQUENCY	PPM	HEIGHT	INDEX	FREQUENCY	PPM	HEIGHT
1	2710.4	6.779	8.2	36	1545.6	3.866	8.4
2	2707.1	6.770	8.2	37	1510.8	3.779	132.8
3	2695.1	6.741	8.3	38	1510.0	3.777	121.4
4	2693.8	6.737	10.0	39	1500.4	3.753	2.1
5	2692.8	6.735	9.8	40	1494.4	3.737	3.3
6	2691.4	6.731	8.1	41	1489.7	3.726	3.2
7	2250.9	5.629	21.4	42	1482.8	3.709	1.7
8	1825.0	4.564	5.4	43	1393.4	3.485	99.5
9	1821.7	4.556	5.7	44	1327.3	3.320	7.4
10	1780.4	4.453	3.0	45	1322.0	3.306	9.6
11	1779.0	4.449	3.3	46	1255.5	3.140	1.8
12	1777.3	4.445	3.1	47	1250.2	3.127	1.6
13	1774.5	4.438	3.1	48	1143.2	2.859	83.2
14	1754.2	4.387	1.5	49	1129.9	2.826	6.1
15	1749.9	4.376	3.2	50	826.1	2.066	17.1
16	1746.9	4.369	3.7	51	824.0	2.061	34.0
17	1743.0	4.359	10.7	52	821.8	2.055	48.9
18	1738.1	4.347	9.7	53	819.7	2.050	32.1
19	1735.2	4.340	8.2	54	817.3	2.044	16.7
20	1731.3	4.330	7.7	55	0.0	0.000	25.7
21	1706.4	4.268	2.0				
22	1702.5	4.258	3.2				
23	1698.6	4.248	5.5				
24	1695.5	4.240	5.3				
25	1690.2	4.227	4.2				
26	1664.4	4.163	6.9				
27	1656.4	4.143	6.1				
28	1631.5	4.080	5.1				
29	1624.5	4.063	4.6				
30	1596.9	3.994	6.3				
31	1594.7	3.988	6.8				
32	1593.2	3.985	6.7				
33	1591.0	3.979	5.9				
34	1555.8	3.891	6.7				
35	1550.5	3.878	3.6				

Figure S48: ^{13}C-NMR Spectrum of (-)-**18** in acetone-d$_6$ (400 MHz)

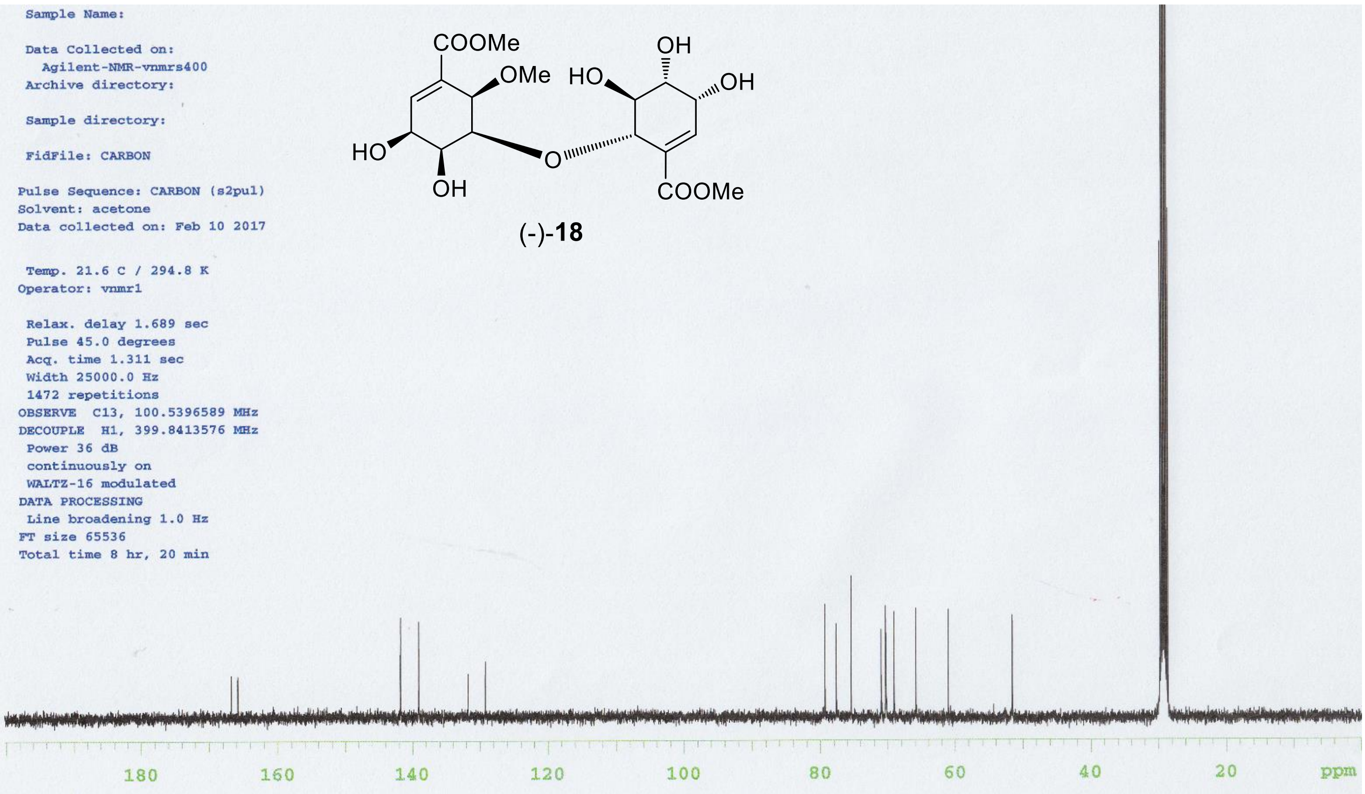

]

INDEX	FREQUENCY	PPM	HEIGHT
1	20684.1	205.731	21.0
2	20665.8	205.548	869.3
3	20645.2	205.343	11.0
4	16768.7	166.787	7.9
5	16672.5	165.830	7.6
6	14259.4	141.828	19.2
7	13988.5	139.134	18.4
8	13249.2	131.781	8.2
9	12993.6	129.239	10.5
10	7978.8	79.360	21.6
11	7809.5	77.676	17.8
12	7587.5	75.467	27.2
13	7141.1	71.028	16.7
14	7076.3	70.383	21.3
15	7071.7	70.338	15.9
16	6951.2	69.139	20.1
17	6628.4	65.929	20.8
18	6139.4	61.064	20.5
19	5188.8	51.609	19.5
20	5183.4	51.556	18.2
21	2993.8	29.777	92.5
22	2974.7	29.588	312.5
23	2955.7	29.398	592.4
24	2935.8	29.201	666.0
25	2916.7	29.011	621.5
26	2897.7	28.821	283.4
27	2877.8	28.624	99.0

Figure S49: ^{1}H- NMR Spectrum of (+)-**18** in acetone-d_6 (400 MHz)

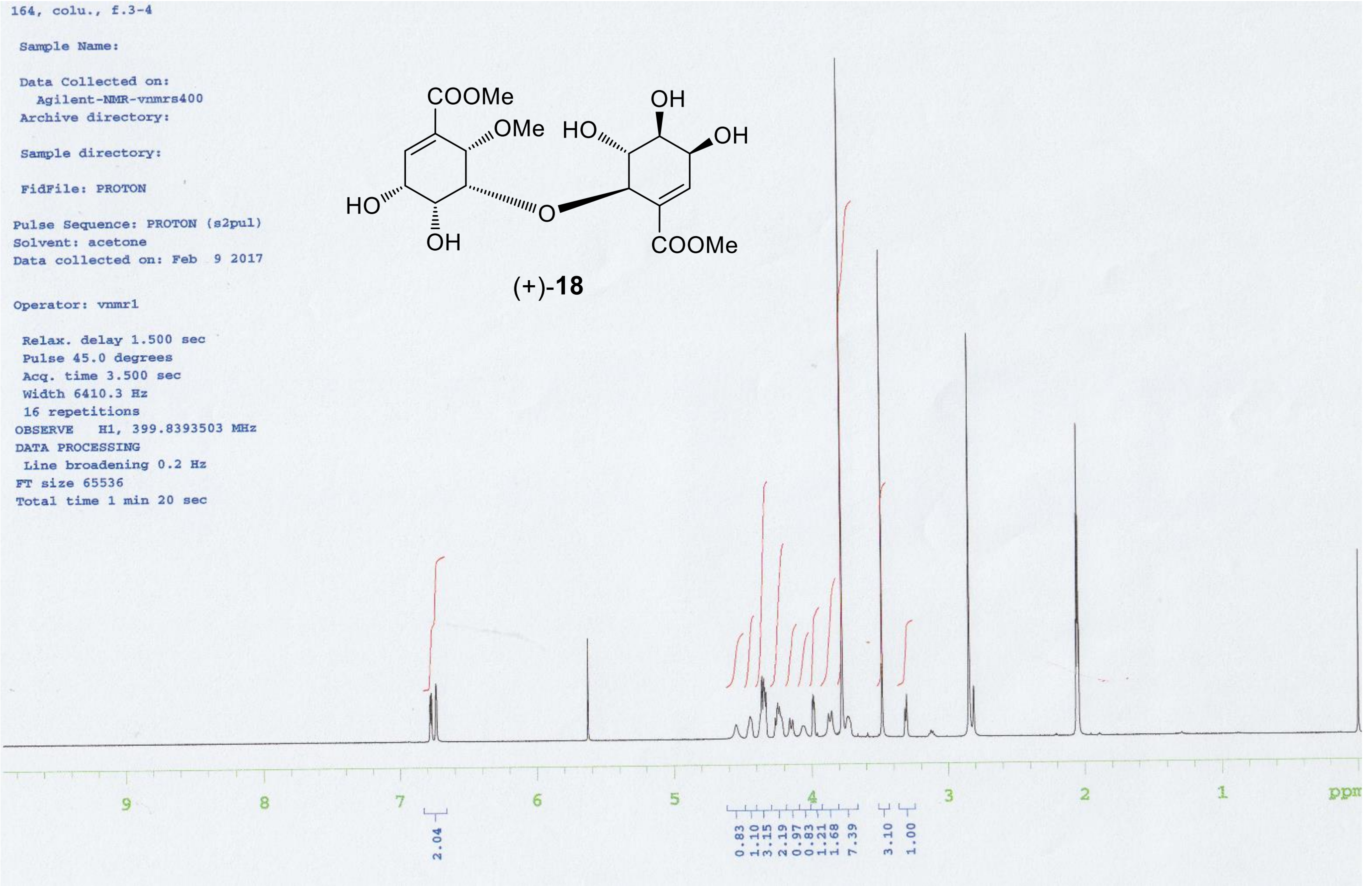

INDEX	FREQUENCY	PPM	HEIGHT	INDEX	FREQUENCY	PPM	HEIGHT
1	2710.4	6.779	32.4	36	823.8	2.060	153.2
2	2707.1	6.770	33.8	37	821.6	2.055	214.8
3	2694.7	6.740	31.8	38	819.3	2.049	151.6
4	2693.4	6.736	40.4	39	817.1	2.044	78.1
5	2692.6	6.734	39.4	40	-0.0	-0.000	126.2
6	2691.2	6.731	30.9				
7	2250.3	5.628	70.4				
8	1819.3	4.550	10.1				
9	1778.2	4.447	15.6				
10	1743.0	4.359	42.9				
11	1738.1	4.347	41.7				
12	1735.4	4.340	35.3				
13	1731.5	4.330	32.1				
14	1704.5	4.263	14.4				
15	1697.1	4.244	24.4				
16	1692.2	4.232	22.2				
17	1662.6	4.158	14.0				
18	1654.4	4.138	12.8				
19	1625.5	4.065	8.9				
20	1596.7	3.993	27.1				
21	1594.7	3.988	29.7				
22	1593.0	3.984	29.1				
23	1591.0	3.979	25.0				
24	1550.1	3.877	16.8				
25	1540.6	3.853	18.8				
26	1510.6	3.778	467.7				
27	1509.8	3.776	434.9				
28	1494.6	3.738	14.9				
29	1492.6	3.733	15.1				
30	1393.6	3.485	335.5				
31	1327.1	3.319	19.4				
32	1322.0	3.306	28.9				
33	1137.8	2.846	277.5				
34	1124.5	2.812	34.7				
35	825.9	2.066	80.1				

Figure S50: ^{13}C-NMR Spectrum of (+)-**18** in acetone-d$_6$ (100 MHz)

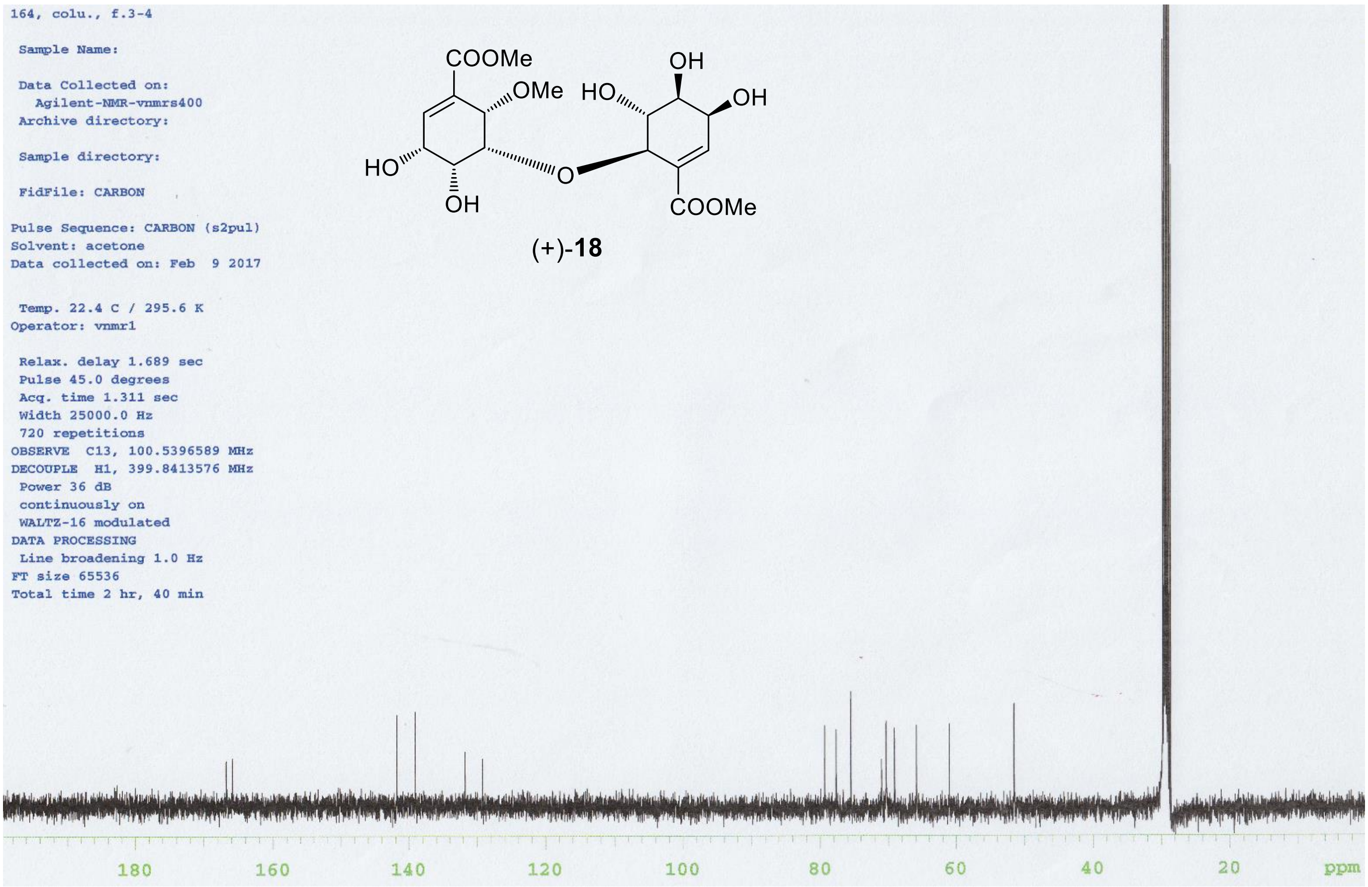

INDEX	FREQUENCY	PPM	HEIGHT
1	16768.7	166.787	11.5
2	16672.6	165.831	12.0
3	14258.6	141.821	22.1
4	13989.3	139.142	22.8
5	13247.7	131.766	13.5
6	12994.4	129.247	11.9
7	7978.1	79.353	19.5
8	7808.0	77.661	18.6
9	7588.3	75.475	27.4
10	7140.4	71.021	11.7
11	7075.6	70.376	20.5
12	6951.2	69.139	18.9
13	6628.5	65.929	19.6
14	6138.7	61.057	19.8
15	5188.8	51.610	24.3
16	5183.5	51.556	24.5
17	2993.8	29.778	177.8
18	2974.0	29.580	447.3
19	2954.9	29.391	1019.5
20	2935.8	29.201	1144.7
21	2916.0	29.004	927.7
22	2896.9	28.814	506.1
23	2877.9	28.624	148.9

Figure S51: ^{1}H- NMR Spectrum of (-)-**8** in acetone-d_6 (600 MHz)

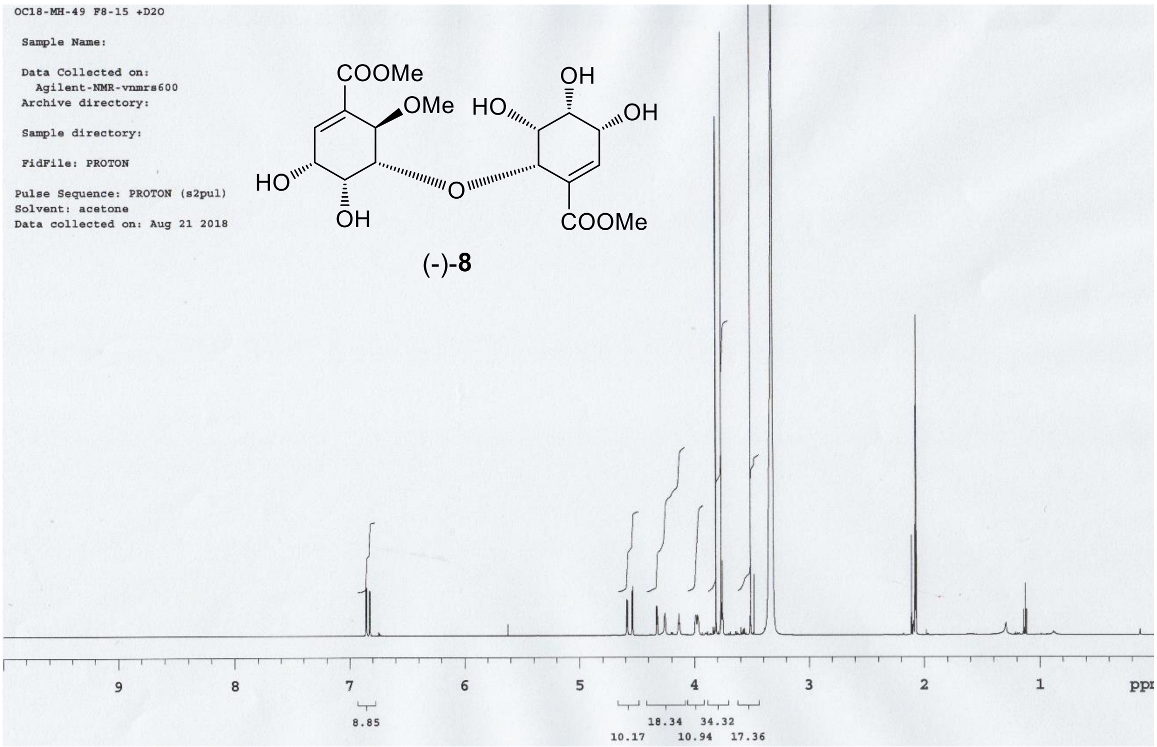

OC18-MH-49 F8-15 +D2O

INDEX	FREQUENCY	PPM	HEIGHT	INDEX	FREQUENCY	PPM	HEIGHT
1	4114.9	6.859	93.7	51	1250.0	2.084	209.8
2	4110.8	6.852	91.8	52	1247.7	2.080	434.3
3	4096.4	6.828	81.0	53	1245.6	2.076	606.8
4	4095.2	6.827	86.3	54	1243.3	2.073	437.2
5	4094.1	6.825	82.9	55	1241.2	2.069	197.5
6	4092.6	6.822	83.6	56	775.3	1.292	24.4
7	3374.0	5.624	21.4	57	683.4	1.139	48.1
8	2752.5	4.588	68.8	58	680.8	1.135	14.9
9	2748.3	4.581	67.1	59	676.4	1.127	98.9
10	2724.6	4.542	85.5	60	669.3	1.116	50.0
11	2720.5	4.535	93.5				
12	2598.4	4.331	55.8				
13	2596.6	4.328	57.0				
14	2594.3	4.325	55.4				
15	2592.2	4.321	50.8				
16	2556.7	4.262	34.2				
17	2553.8	4.257	42.7				
18	2485.1	4.143	22.8				
19	2481.0	4.136	41.1				
20	2476.9	4.129	24.5				
21	2395.6	3.993	37.3				
22	2393.6	3.990	39.2				
23	2391.5	3.987	38.1				
24	2384.5	3.975	39.2				
25	2382.7	3.972	37.8				
26	2380.1	3.967	33.2				
27	2303.5	3.840	16.2				
28	2297.3	3.830	15.9				
29	2287.6	3.813	983.7				
30	2266.5	3.778	17.9				
31	2263.9	3.774	46.6				
32	2260.4	3.768	1145.0				
33	2256.5	3.762	55.8				
34	2254.8	3.759	142.4				
35	2252.1	3.754	42.0				
36	2250.1	3.751	34.0				
37	2155.9	3.594	15.3				
38	2107.5	3.513	1271.3				
39	2104.8	3.509	17.8				
40	2104.0	3.507	20.6				
41	2088.7	3.482	115.6				
42	1993.9	3.324	1488.6				
43	1267.1	2.112	189.7				
44	1266.5	2.111	62.8				
45	1258.9	2.098	20.6				
46	1257.7	2.096	28.3				
47	1256.8	2.095	20.9				
48	1255.6	2.093	26.7				
49	1254.4	2.091	20.0				
50	1253.3	2.089	18.6				

Figure S52: ^{13}C-NMR Spectrum of (-)-**8** in acetone-d_6 (150 MHz)

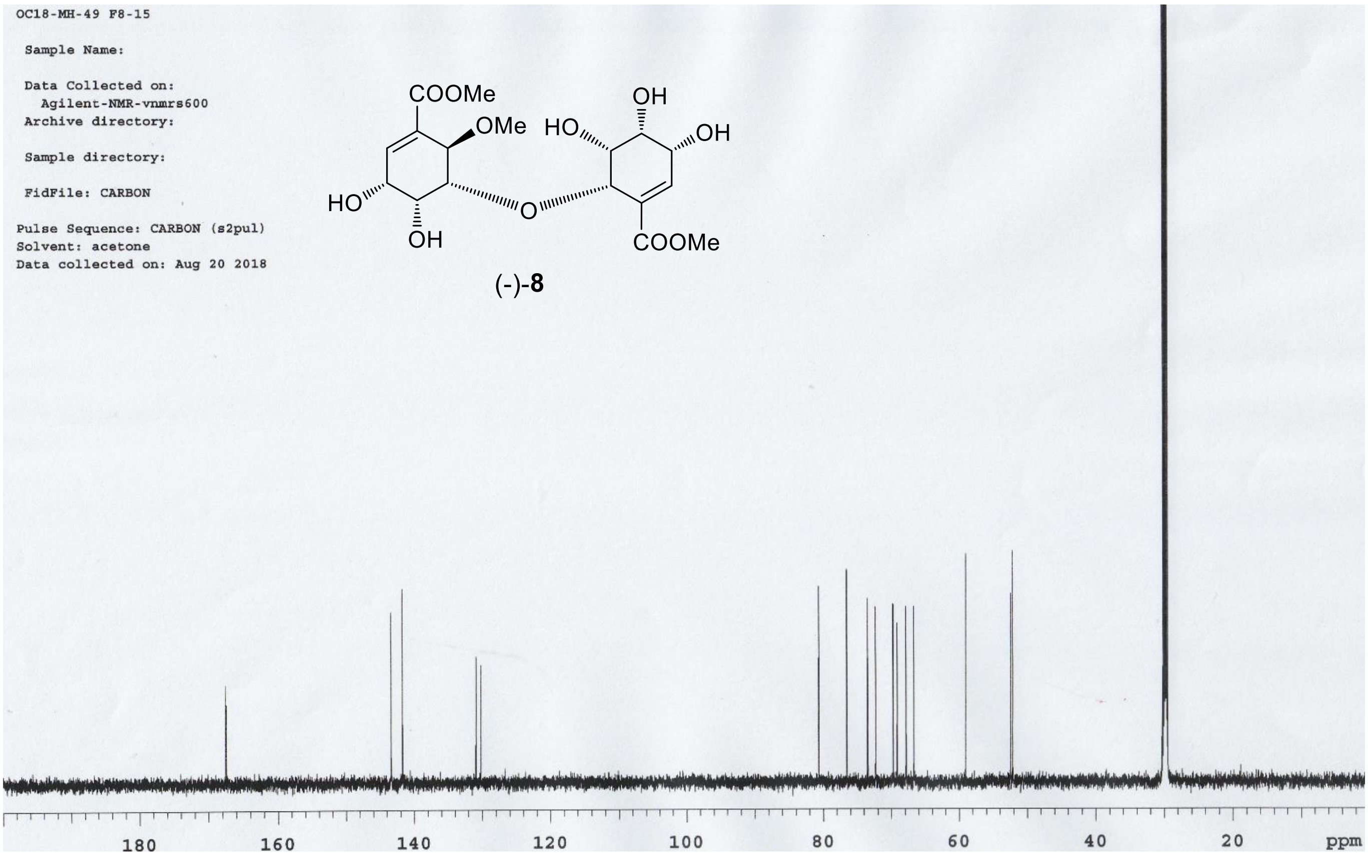

OC18-MH-49 F8-15

NDEX	FREQUENCY	PPM	HEIGHT
1	25268.3	167.512	19.2
2	25248.7	167.382	15.5
3	21622.4	143.342	33.4
4	21372.7	141.687	38.1
5	19740.5	130.866	24.8
6	19630.7	130.138	23.1
7	12159.6	80.610	38.4
8	11537.7	76.487	41.7
9	11077.6	73.437	36.0
10	10901.9	72.273	34.3
11	10512.4	69.690	34.9
12	10425.7	69.115	31.1
13	10228.0	67.805	34.4
14	10059.2	66.686	34.4
15	8899.8	59.000	44.6
16	7912.6	52.455	36.9
17	7864.0	52.133	45.2
18	4577.6	30.347	8.8
19	4559.1	30.224	181.8
20	4539.5	30.094	421.6
21	4521.0	29.971	1085.9
22	4501.3	29.841	1387.4
23	4481.7	29.711	1077.3
24	4463.2	29.588	428.3
25	4443.5	29.458	184.5

Figure S53: ^{1}H- NMR Spectrum of (+)-**8** in acetone-d_6 (400 MHz)

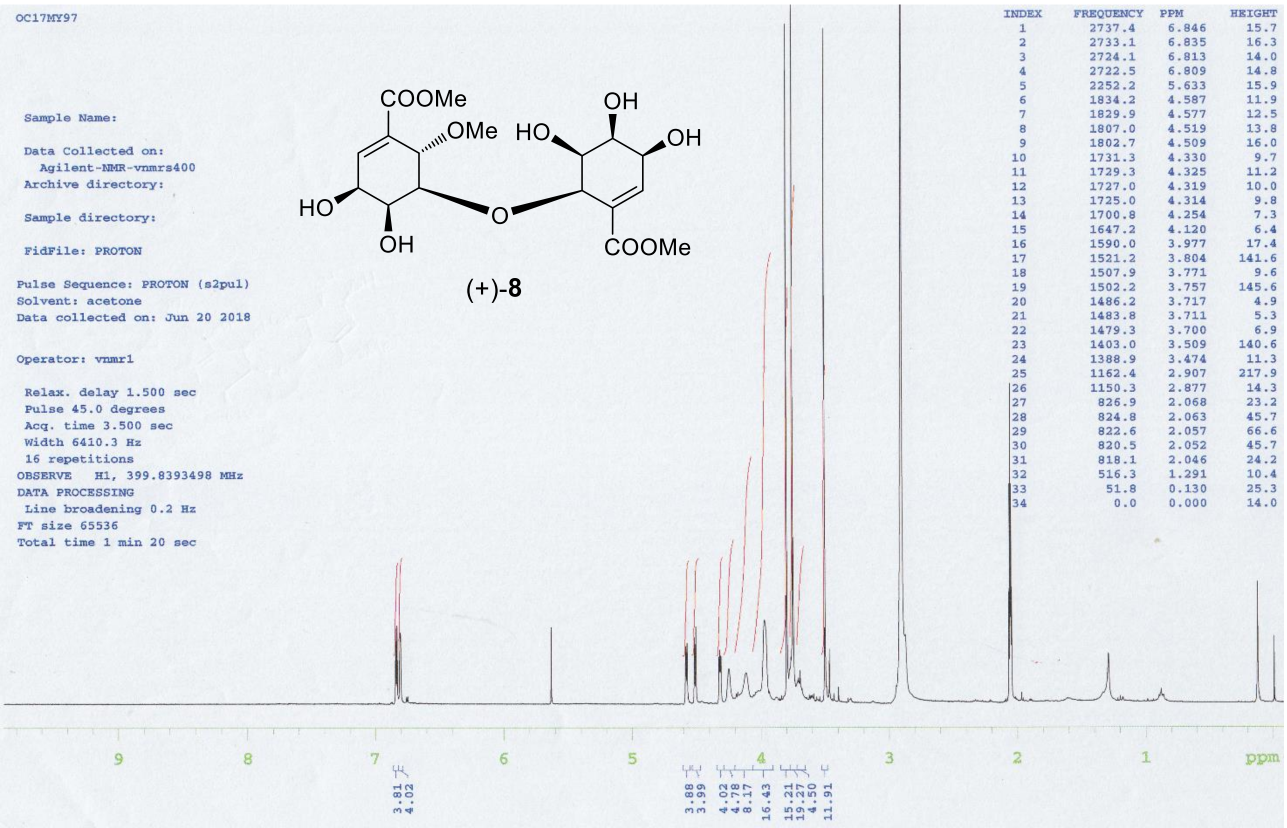

Figure S54: ^{13}C-NMR Spectrum of (+)-**8** in acetone-d_6 (100 MHz)

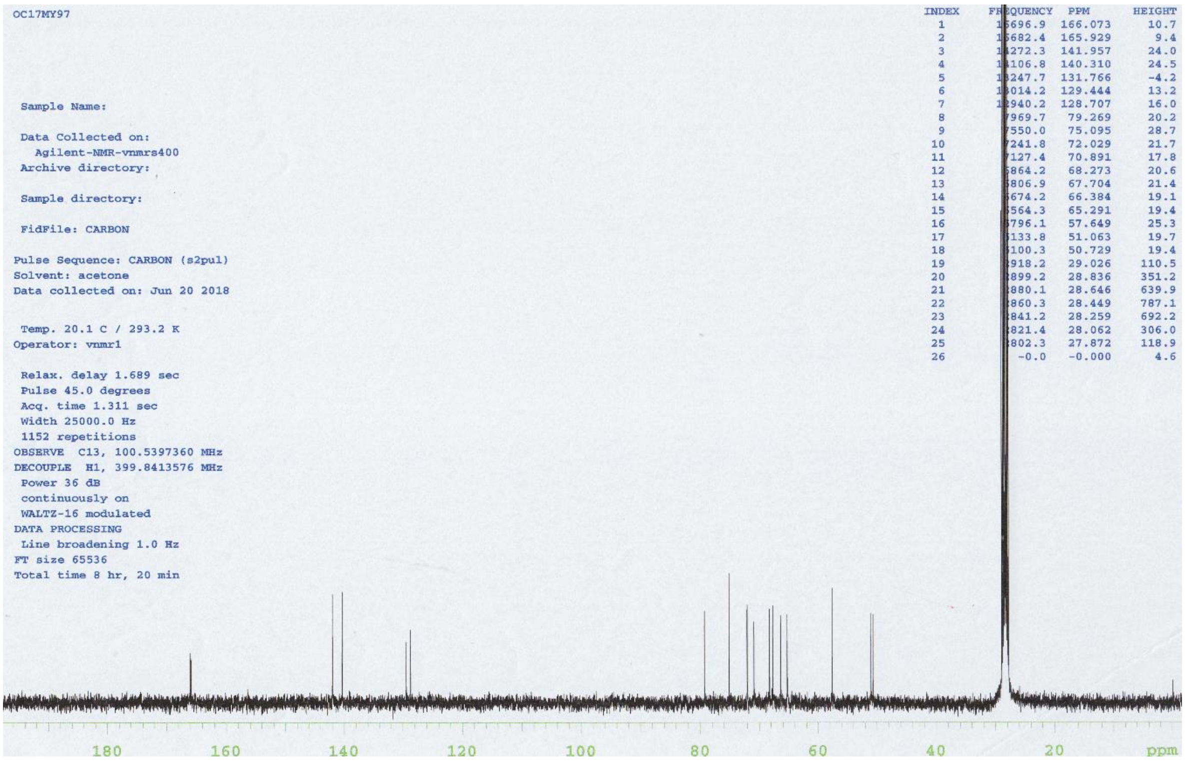

Figure S55: ^{1}H- NMR Spectrum of (-)-**21** in acetone-d_6 (600 MHz)

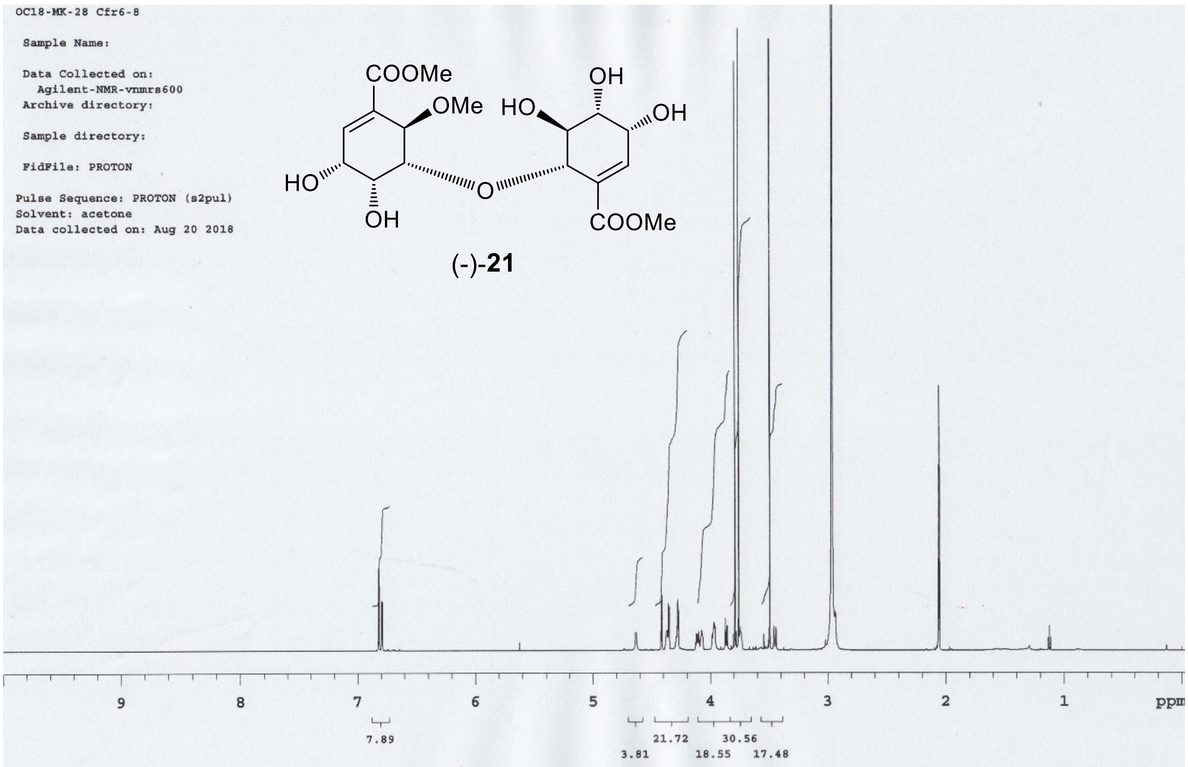

INDEX	FREQUENCY	PPM	HEIGHT	INDEX	FREQUENCY	PPM	HEIGHT
1	4094.1	6.825	103.9	51	1779.7	2.967	4328.6
2	4089.9	6.818	106.7	52	1761.8	2.937	49.4
3	4077.9	6.798	63.0	53	1239.8	2.067	115.4
4	4074.4	6.792	61.8	54	1237.4	2.063	233.9
5	3374.0	5.624	10.5	55	1235.4	2.059	333.8
6	2780.0	4.634	23.3	56	1233.0	2.055	231.1
7	2777.7	4.630	23.4	57	1231.0	2.052	111.5
8	2650.6	4.418	63.4	58	680.8	1.135	16.0
9	2646.8	4.412	70.1	59	674.0	1.124	31.8
10	2620.4	4.368	25.7	60	667.0	1.112	16.7
11	2613.7	4.357	59.6	61	78.1	0.130	7.4
12	2609.3	4.349	55.9				
13	2572.9	4.289	18.1				
14	2568.2	4.281	61.7				
15	2566.1	4.278	64.8				
16	2564.1	4.274	55.5				
17	2562.0	4.271	50.0				
18	2475.2	4.126	11.1				
19	2470.8	4.119	21.7				
20	2465.5	4.110	20.2				
21	2460.5	4.101	24.7				
22	2456.1	4.094	14.2				
23	2444.3	4.075	26.2				
24	2392.4	3.988	17.8				
25	2390.3	3.985	20.0				
26	2387.7	3.980	22.0				
27	2385.4	3.976	26.9				
28	2383.6	3.973	35.4				
29	2381.5	3.970	36.3				
30	2378.9	3.966	34.0				
31	2376.8	3.962	31.4				
32	2327.3	3.879	8.9				
33	2323.7	3.874	40.8				
34	2314.6	3.858	32.1				
35	2283.2	3.806	21.3				
36	2275.0	3.792	743.3				
37	2271.5	3.786	11.1				
38	2268.0	3.781	24.2				
39	2257.7	3.763	40.2				
40	2255.4	3.760	785.1				
41	2251.3	3.753	18.3				
42	2247.2	3.746	30.3				
43	2244.8	3.742	23.4				
44	2242.2	3.738	23.7				
45	2126.5	3.545	21.0				
46	2104.5	3.508	13.9				
47	2097.5	3.496	771.7				
48	2075.5	3.460	30.8				
49	2065.2	3.443	29.4				
50	1812.0	3.020	13.1				

Figure S56: ^{13}C-NMR Spectrum of (-)-**21** in acetone-d_6 (150 MHz)

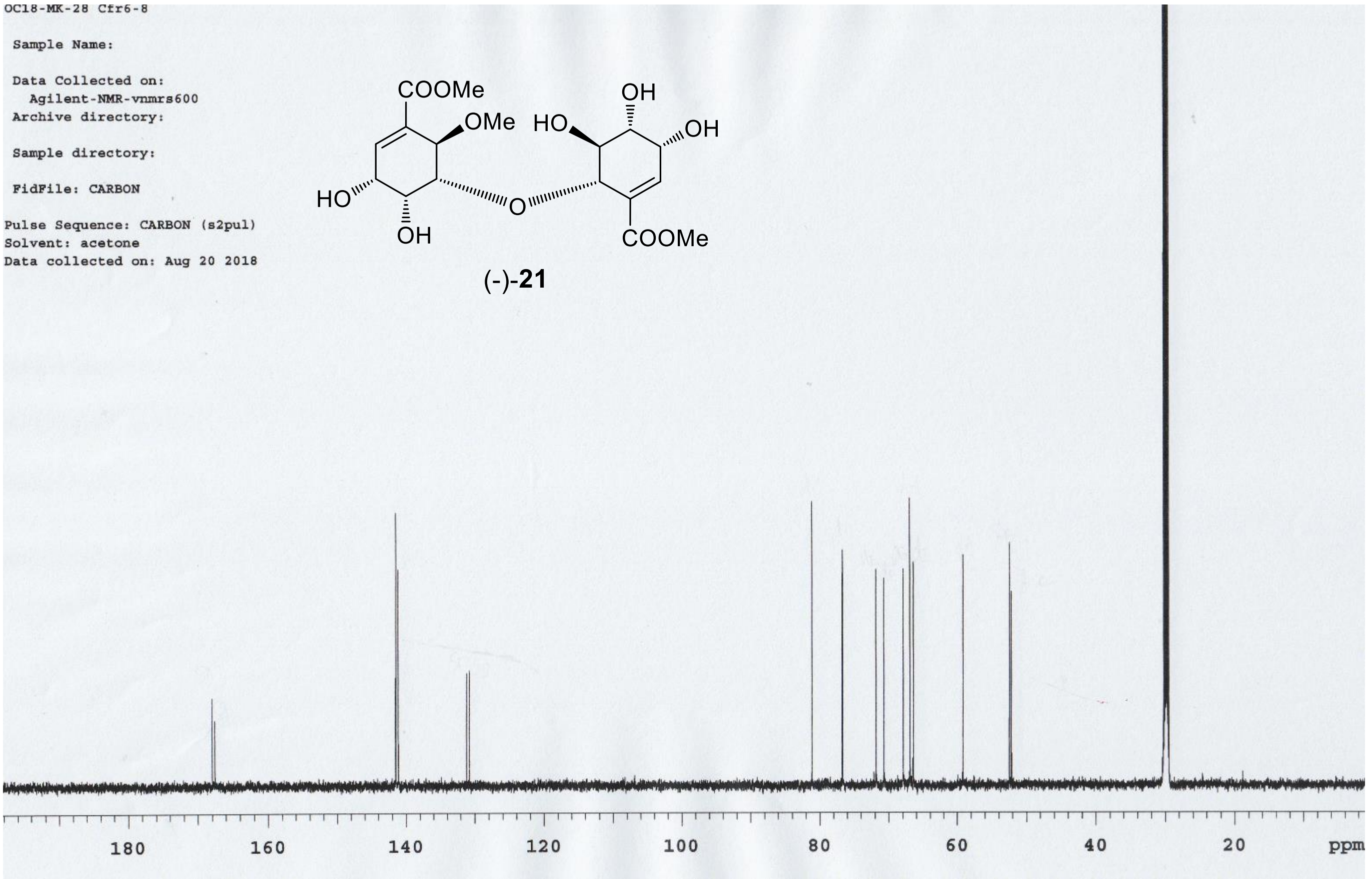

OC18-MK-28 Cfr6-8

INDEX	FREQUENCY	PPM	HEIGHT
1	25333.1	167.942	17.2
2	25274.1	167.551	12.9
3	21340.4	141.473	52.8
4	21286.0	141.112	41.9
5	19784.4	131.158	21.8
6	19723.2	130.752	22.5
7	12232.5	81.093	54.9
8	11569.0	76.695	41.0
9	11563.2	76.656	45.5
10	10832.6	71.813	41.8
11	10660.4	70.671	40.0
12	10240.7	67.889	41.8
13	10093.9	66.916	55.5
14	10021.1	66.433	43.2
15	8926.4	59.176	44.5
16	7913.8	52.463	46.8
17	7868.7	52.164	37.4
18	4577.7	30.347	8.1
19	4559.2	30.224	159.0
20	4540.7	30.102	429.3
21	4521.0	29.971	1051.0
22	4501.4	29.841	1267.3
23	4481.7	29.711	929.8
24	4463.2	29.588	433.4
25	4443.6	29.458	176.6

Figure S57: ^{1}H- NMR Spectrum of (+)-**21** in acetone-d$_6$ (400 MHz)

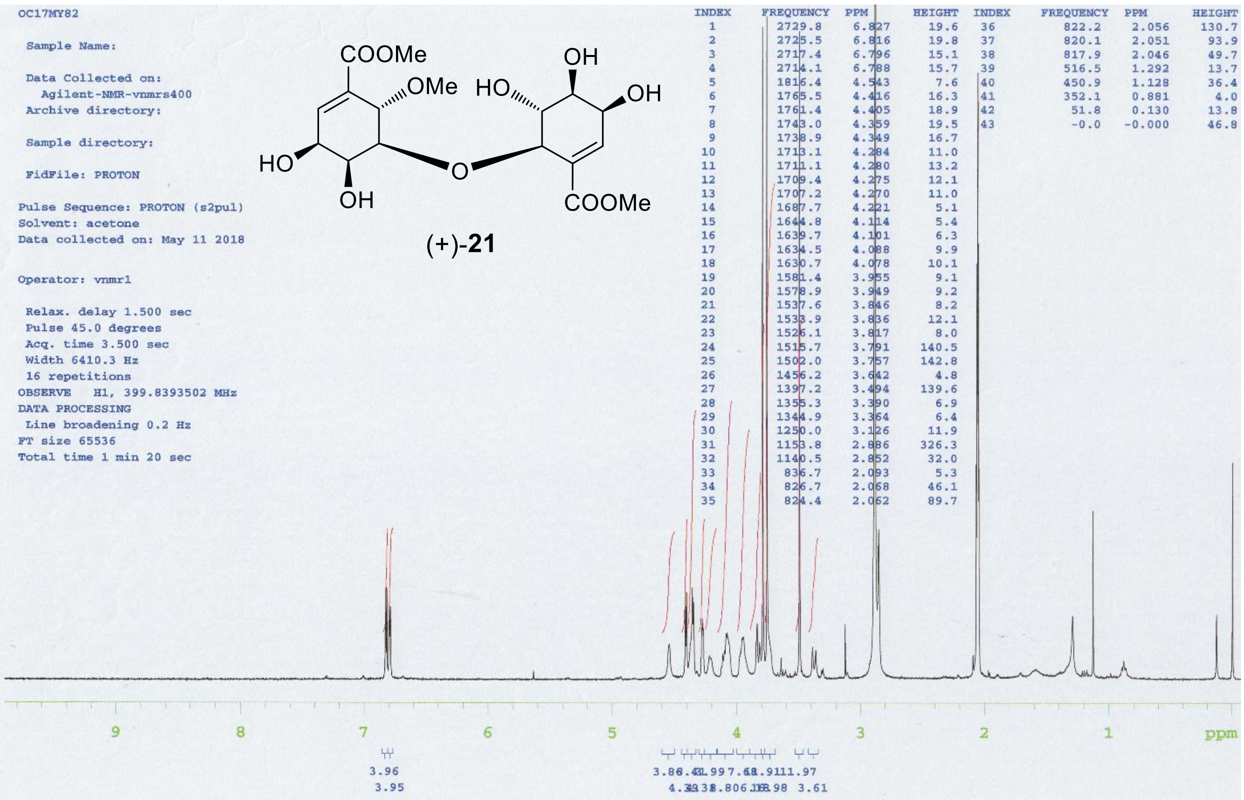

Figure S58: ^{13}C-NMR Spectrum of (+)-**21** in acetone-d$_6$ (100 MHz)

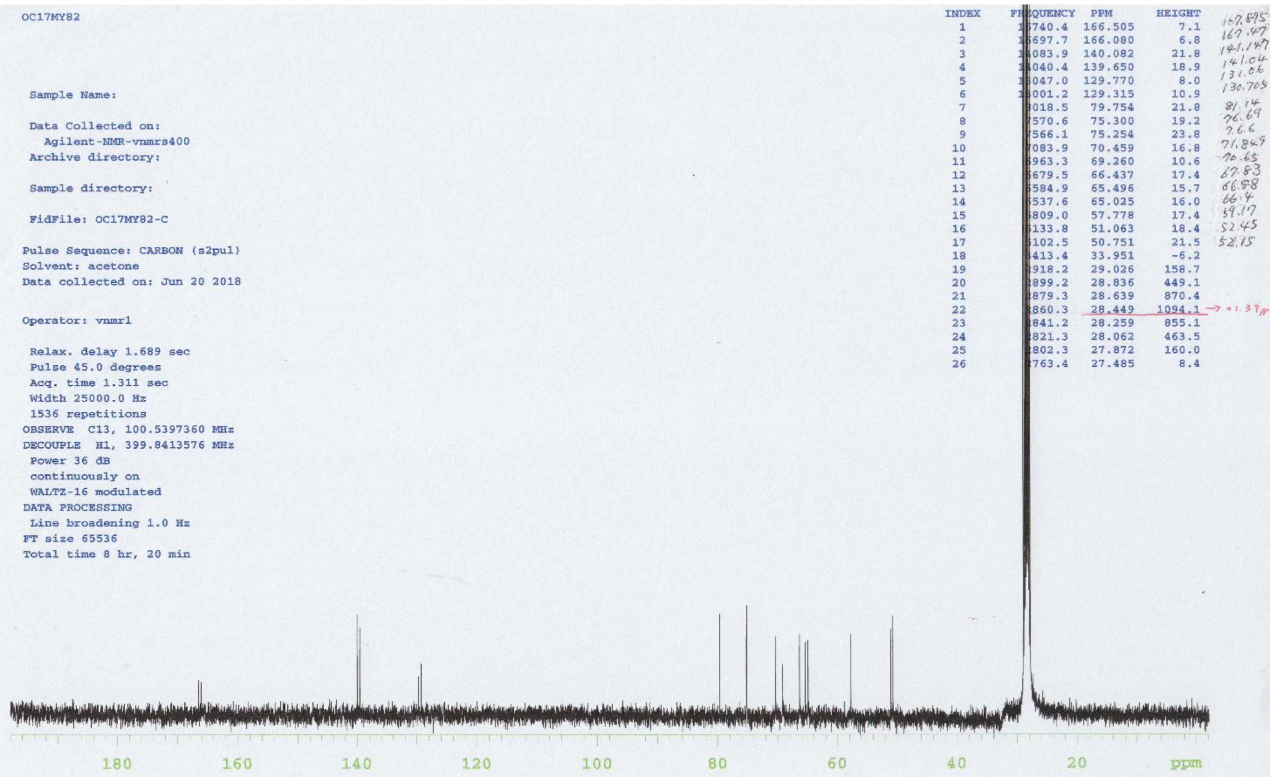

Figure S59: ^{1}H- NMR Spectrum of (-)-**24** in acetone-d_6 (600 MHz)

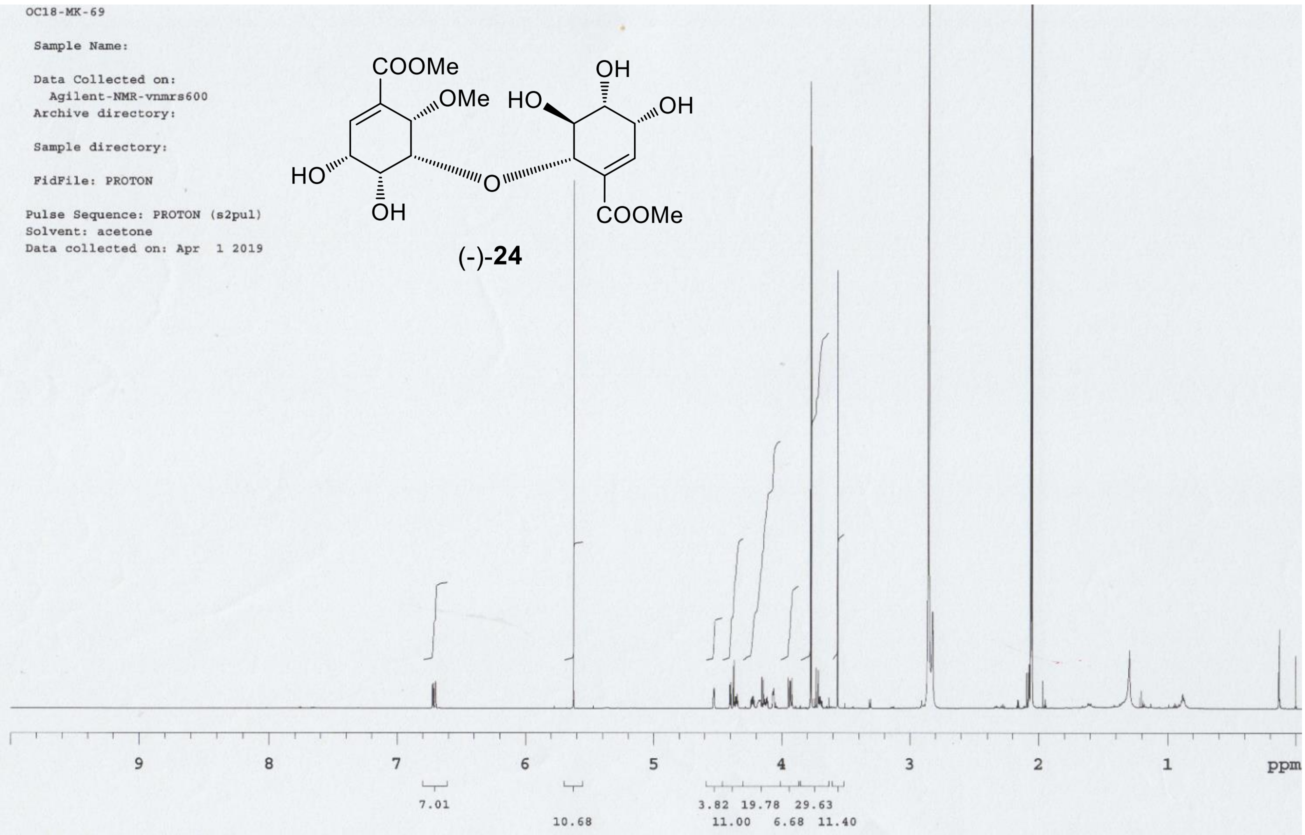

INDEX	FREQUENCY	PPM	HEIGHT	INDEX	FREQUENCY	PPM	HEIGHT
1	2729.8	6.827	25.1	36	835.7	2.090	2.6
2	2727.8	6.822	25.5	37	830.0	2.076	2.4
3	2692.2	6.733	25.0	38	825.5	2.065	122.0
4	2690.0	6.728	24.7	39	823.4	2.059	253.4
5	2250.5	5.628	87.4	40	821.2	2.054	369.1
6	1880.0	4.702	3.4	41	819.1	2.049	236.8
7	1868.6	4.673	17.6	42	816.7	2.043	124.1
8	1864.9	4.664	17.7	43	757.5	1.894	2.9
9	1787.2	4.470	8.6	44	534.3	1.336	4.0
10	1727.6	4.321	19.4	45	516.6	1.292	17.6
11	1723.9	4.311	20.9	46	506.3	1.266	3.0
12	1696.3	4.242	19.4	47	498.3	1.246	3.9
13	1680.2	4.202	4.9	48	479.9	1.200	3.2
14	1638.2	4.097	16.4	49	473.0	1.183	3.4
15	1636.0	4.092	15.8	50	471.1	1.178	4.8
16	1604.7	4.013	13.0	51	394.8	0.987	3.7
17	1589.5	3.975	7.3	52	384.0	0.960	2.7
18	1580.5	3.953	7.0	53	376.4	0.941	4.2
19	1533.9	3.836	11.6	54	369.1	0.923	2.6
20	1516.9	3.794	391.5	55	361.9	0.905	2.9
21	1507.9	3.771	383.1	56	359.0	0.898	4.9
22	1504.8	3.763	11.2	57	355.3	0.888	5.6
23	1499.3	3.750	9.3	58	352.1	0.881	6.6
24	1461.5	3.655	7.1	59	348.4	0.871	4.6
25	1450.4	3.627	8.5	60	345.7	0.865	3.5
26	1449.2	3.624	2.7	61	51.4	0.129	5.4
27	1443.1	3.609	2.9	62	4.3	0.011	3.2
28	1433.2	3.584	2.6	63	3.1	0.008	18.9
29	1416.7	3.543	4.9	64	2.3	0.006	7.5
30	1385.6	3.465	221.4	65	-0.0	-0.000	594.1
31	1348.6	3.373	3.3	66	-2.2	-0.005	6.5
32	1326.9	3.319	3.3	67	-3.5	-0.009	15.3
33	1321.6	3.305	5.1				
34	1131.9	2.831	145.7				
35	1118.4	2.797	35.7				

Figure S60: ^{13}C-NMR Spectrum of (-)-**24** in acetone-d_6 (150 MHz)

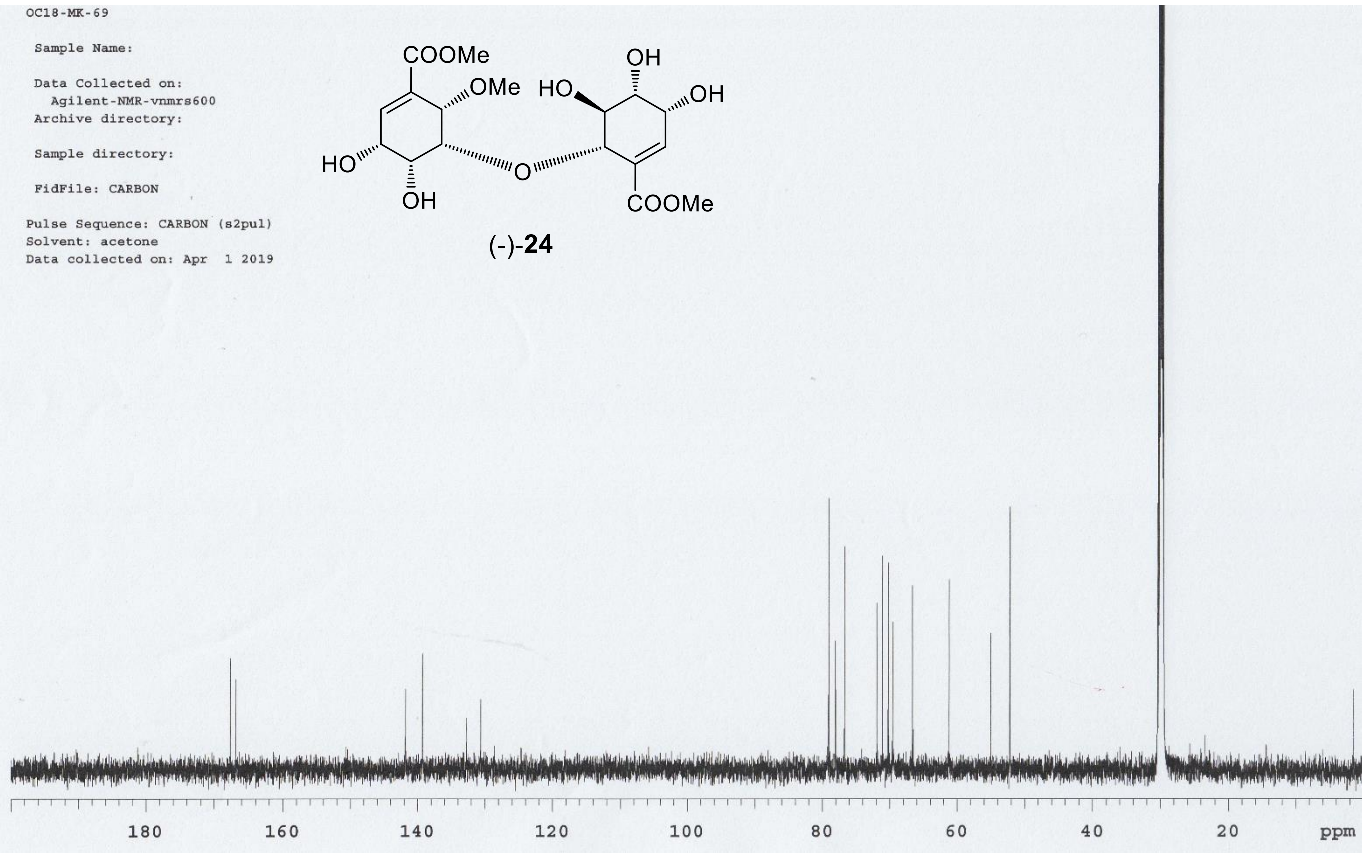

OC18-MK-69

INDEX	FREQUENCY	PPM	HEIGHT
1	25288.0	167.643	22.6
2	25160.8	166.800	18.6
3	21386.6	141.779	16.6
4	21008.6	139.273	23.6
5	20022.5	132.736	10.8
6	19698.9	130.590	14.6
7	19391.4	128.552	5.3
8	15953.5	105.761	5.2
9	11952.7	79.239	5.2
10	11920.3	79.024	54.2
11	11770.1	78.028	26.1
12	11564.3	76.664	44.8
13	10843.0	71.882	33.5
14	10720.4	71.070	42.8
15	10588.7	70.196	41.5
16	10574.8	70.104	7.3
17	10490.4	69.545	29.8
18	10048.8	66.617	37.0
19	10032.6	66.510	8.6
20	9222.3	61.138	38.2
21	8290.6	54.961	27.5
22	7869.8	52.172	51.3
23	7862.9	52.126	52.5
24	4583.4	30.385	24.0
25	4577.6	30.347	53.3
26	4559.1	30.224	1098.4
27	4539.5	30.094	2601.0
28	4521.0	29.971	5916.5
29	4501.3	29.841	7991.3
30	4481.7	29.711	6443.9
31	4462.0	29.580	2507.9
32	4443.5	29.458	1013.5
33	3865.6	25.626	5.8
34	3518.8	23.327	7.5
35	2165.1	14.353	5.5
36	211.5	1.402	16.4

Figure 61: ^{1}H- NMR Spectrum of (+)-**24** in acetone-d_6 (400 MHz)

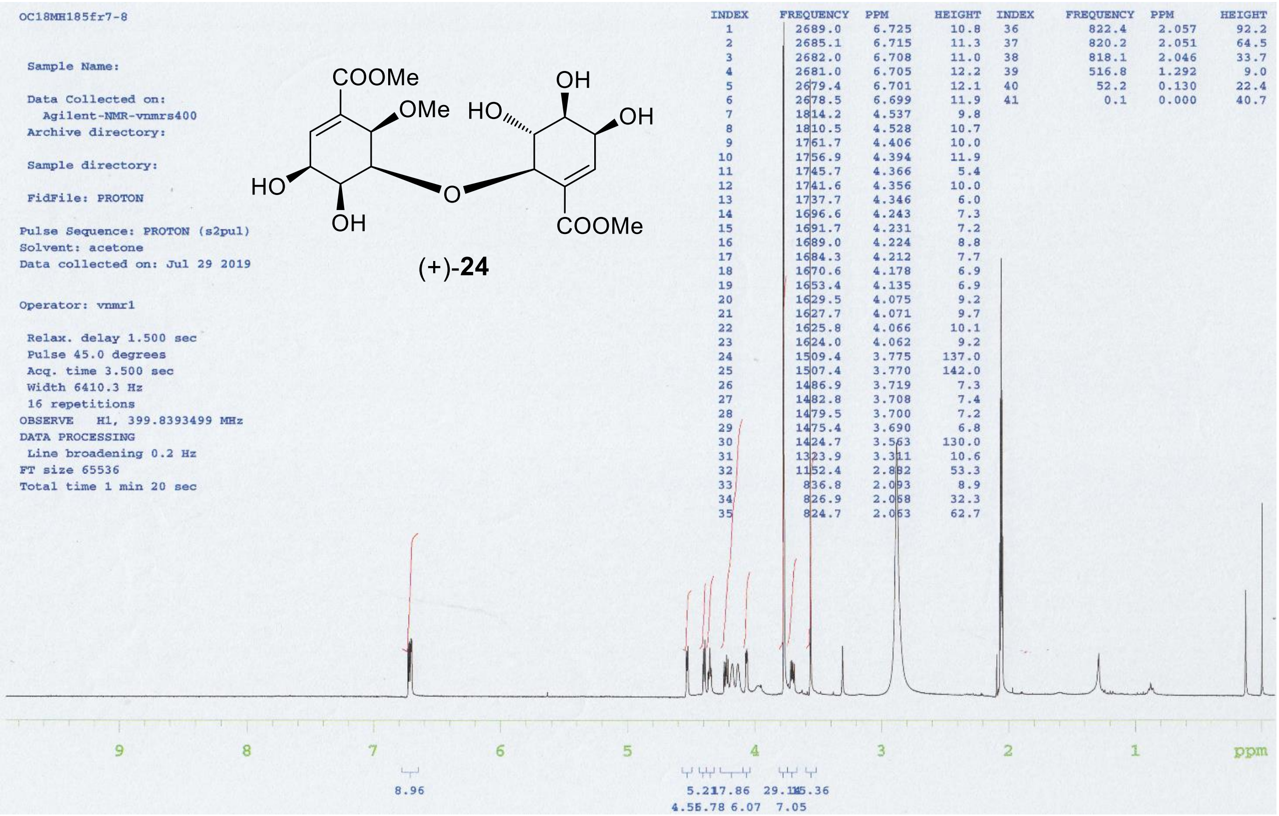

Figure S62: ^{13}C-NMR Spectrum of (+)-**24** in acetone-d_6 (100 MHz)

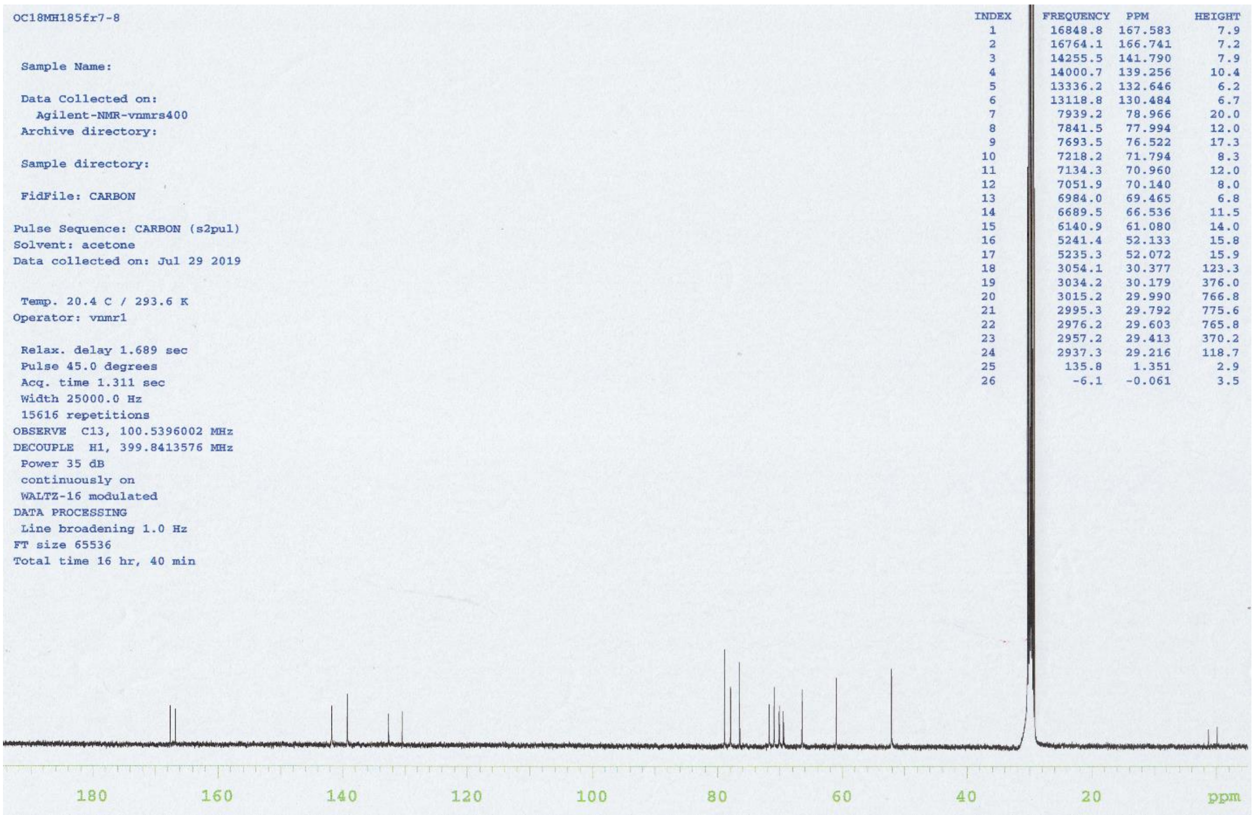

Figure S63: ^{1}H- NMR Spectrum of (-)-**9** in $CDCl_3$ (600 MHz)

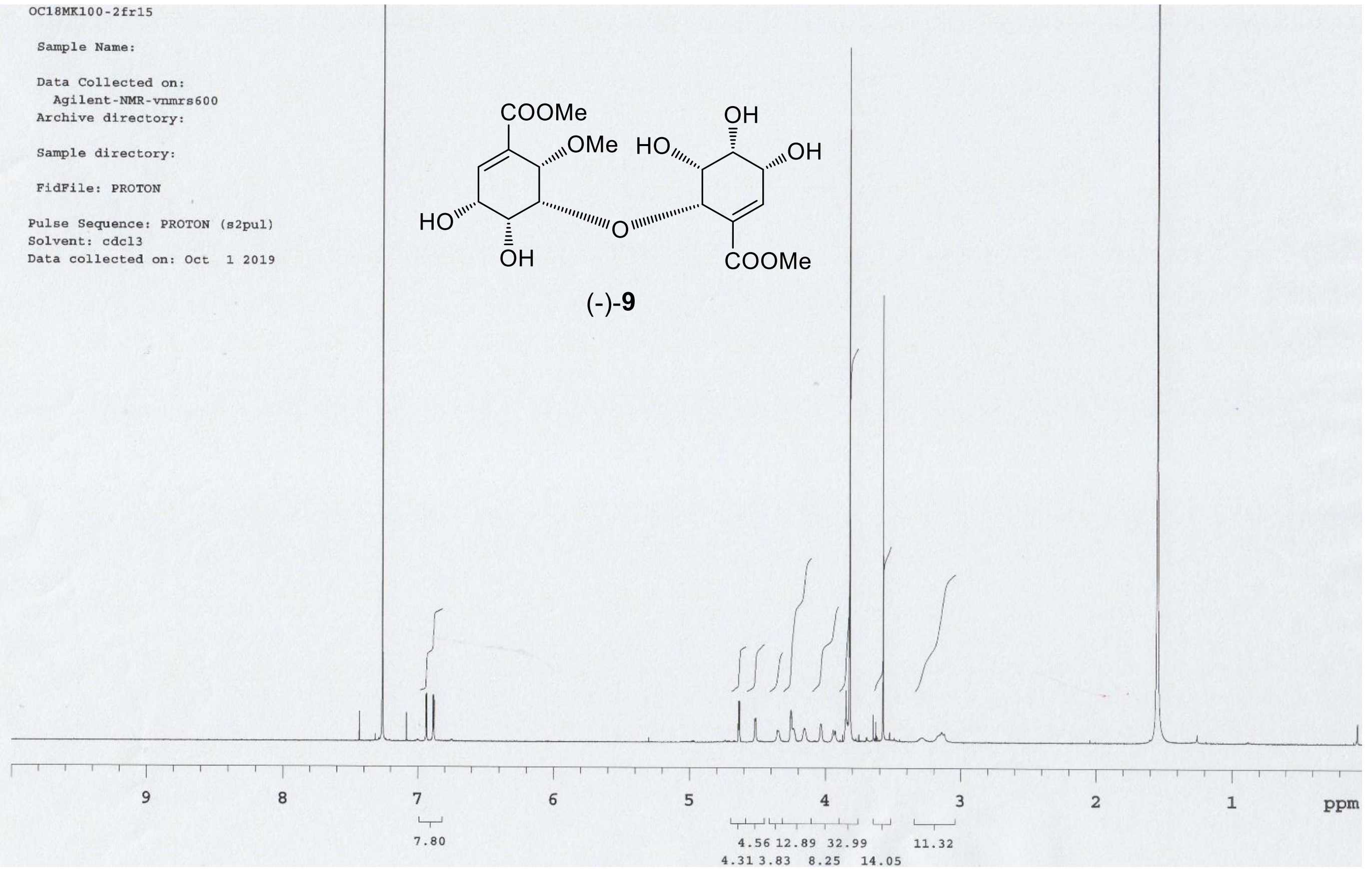

OC18MK100-2fr15 +D2O

INDEX	FREQUENCY	PPM	HEIGHT
1	4460.3	7.435	31.7
2	4357.3	7.263	5175.1
3	4251.0	7.086	34.5
4	4159.5	6.934	46.9
5	4156.6	6.929	47.0
6	4157.1	6.930	47.4
7	4119.6	6.867	41.1
8	4115.8	6.861	42.6
9	2851.3	4.753	906.1
10	2778.0	4.631	41.3
11	2773.6	4.623	40.3
12	2692.3	4.488	29.0
13	2689.4	4.483	28.7
14	2563.5	4.273	28.8
15	2538.2	4.231	20.5
16	2534.4	4.225	30.0
17	2530.9	4.219	20.0
18	2486.3	4.145	22.4
19	2481.6	4.137	30.7
20	2477.5	4.130	21.3
21	2423.5	4.040	24.1
22	2294.7	3.825	545.7
23	2291.8	3.820	545.0
24	2275.9	3.794	31.5
25	2175.8	3.627	10.6
26	2146.8	3.579	290.7
27	2107.8	3.514	10.0
28	939.9	1.567	46.9
29	752.4	1.254	11.8
30	41.7	0.069	24.7
31	3.2	0.005	29.1
32	-0.0	-0.000	968.9
33	-3.2	-0.005	26.1

Figure S64: ^{13}C-NMR Spectrum of (-)-**9** in acetone-d_6 (150 MHz)

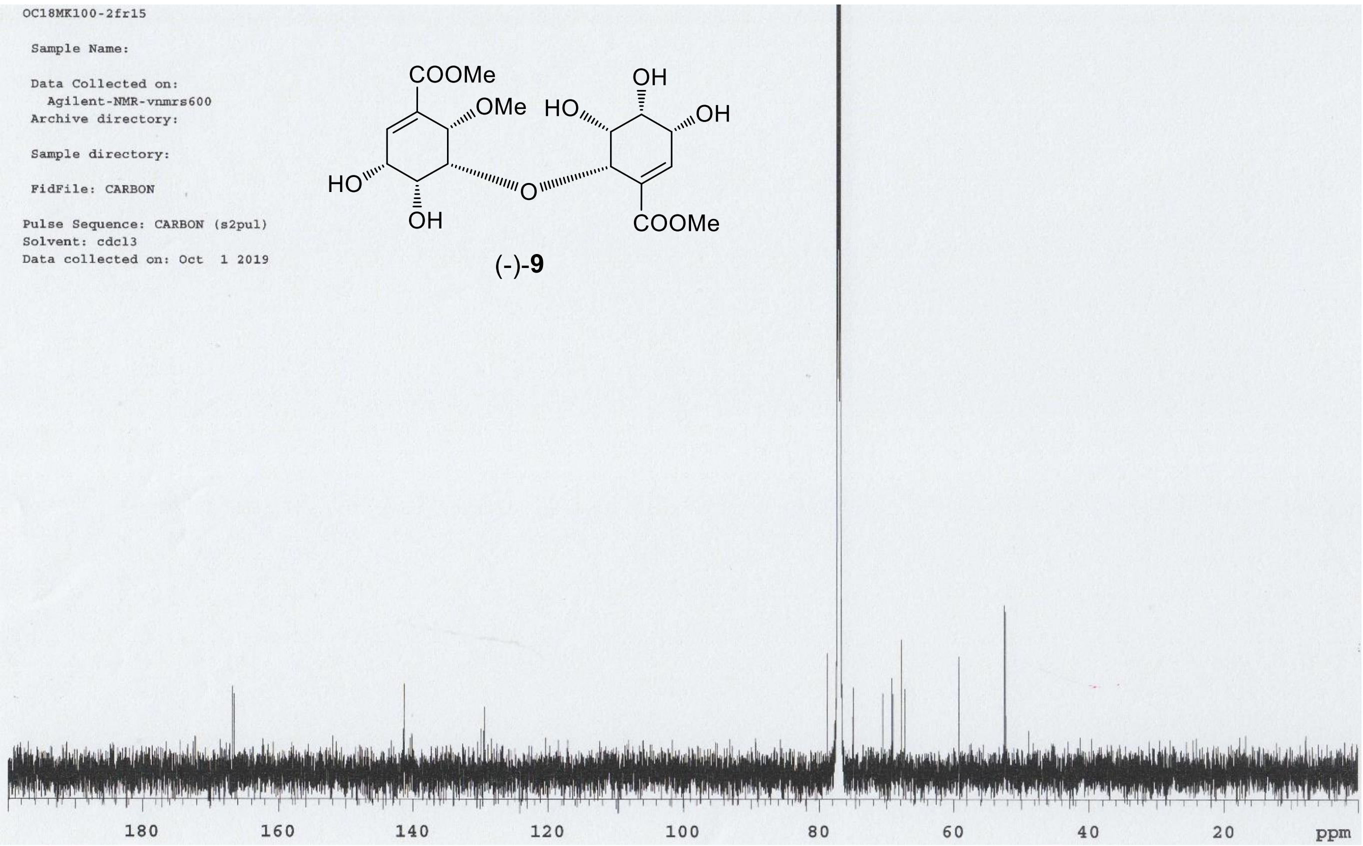

OC18MK100-2fr15

INDEX	FREQUENCY	PPM	HEIGHT
1	25150.2	166.730	17.2
2	25106.3	166.439	15.8
3	21320.5	141.341	17.7
4	19592.3	129.885	8.8
5	19520.7	129.410	13.1
6	11878.5	78.747	23.8
7	11646.2	77.207	5404.4
8	11615.0	77.000	6019.3
9	11582.6	76.785	5865.7
10	11293.6	74.870	17.0
11	10641.7	70.547	15.9
12	10440.5	69.214	18.9
13	10413.9	69.038	15.7
14	10229.0	67.812	26.5
15	10156.1	67.329	16.8
16	8929.7	59.198	23.1
17	7920.5	52.508	33.3
18	7888.1	52.293	32.0
19	7376.0	48.899	8.5

Figure S65: ^{1}H- NMR Spectrum of (+)-**9** in acetone-d_6 (400 MHz)

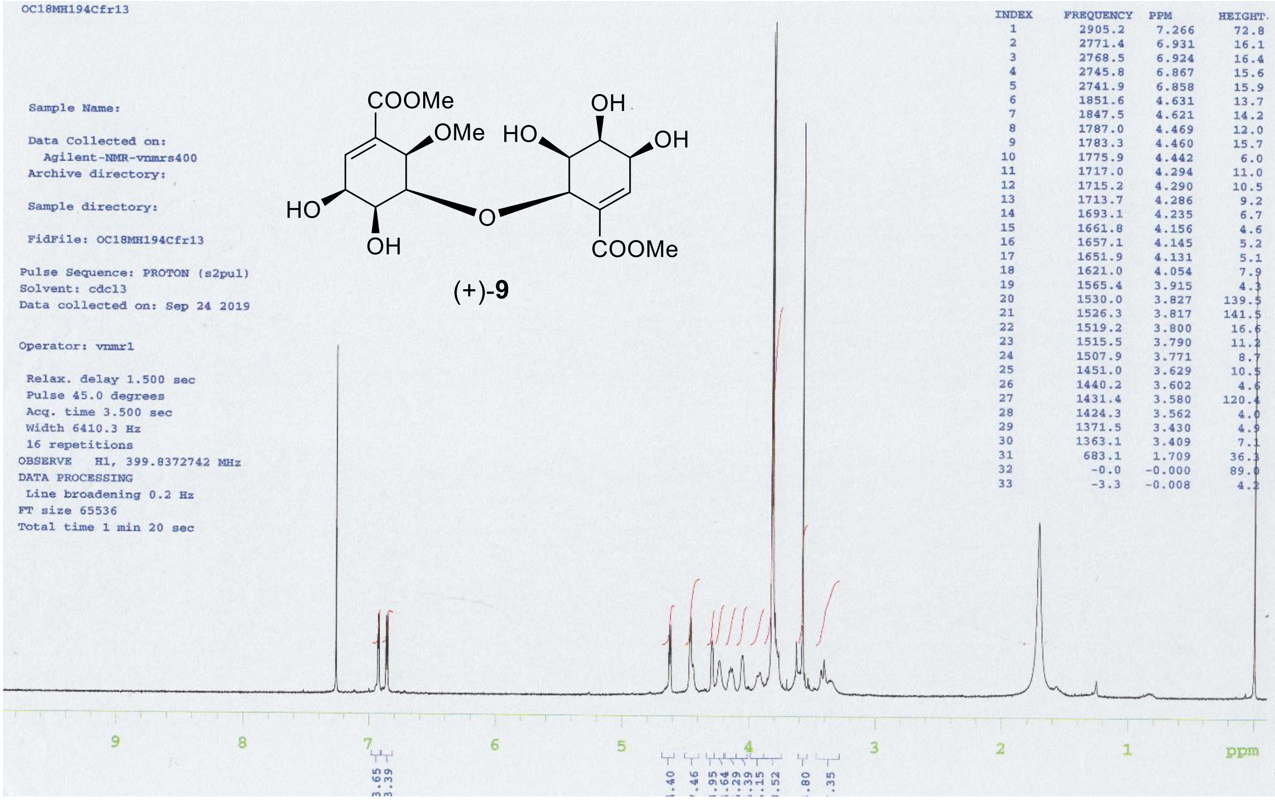

Figure S66: ^{13}C-NMR Spectrum of (+)-**9** in acetone-d_6 (100 MHz)

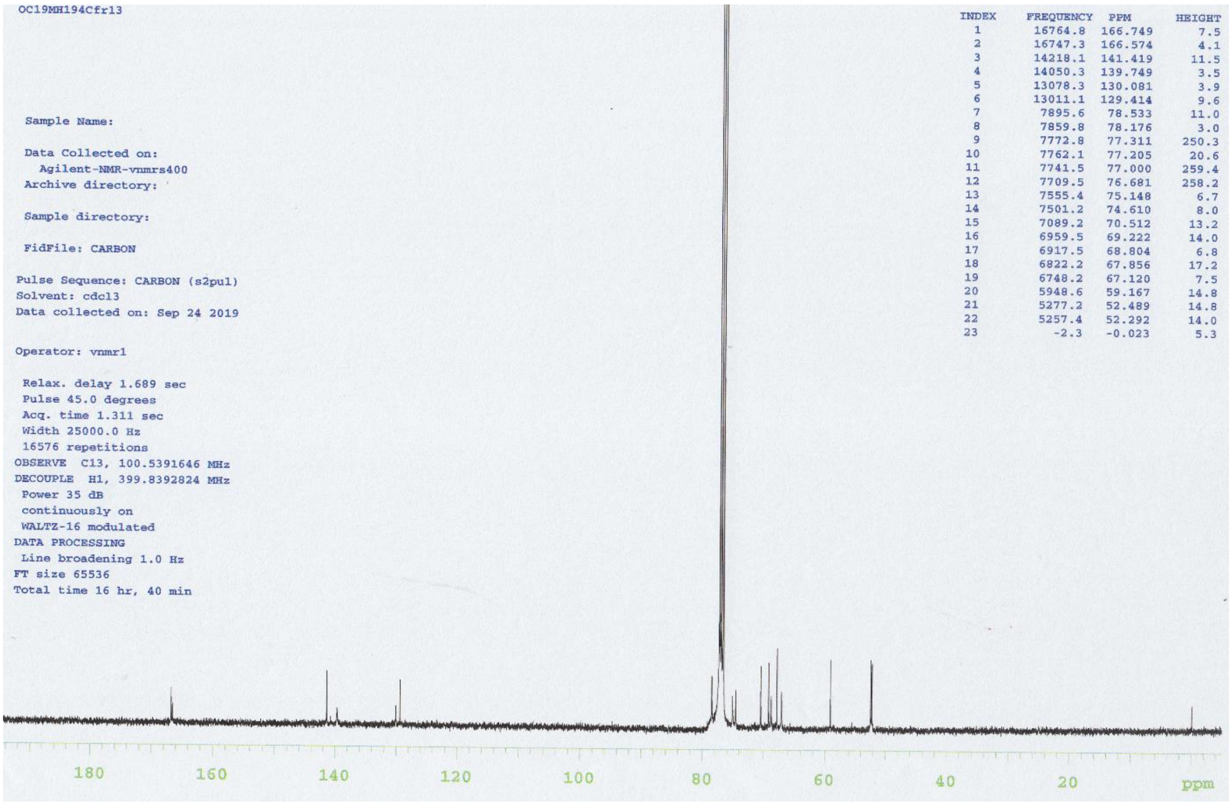

Supplement: Supplementary file 1 [file marinedrugs-18-00221-s001.pdf]
